# Air pollution and fetal brain morphological development: a prospective cohort study

**DOI:** 10.1016/S2542-5196(25)00093-2

**Published:** 2025-06-11

**Authors:** Laura Gómez-Herrera, Yu Zhao, Ioar Rivas, Elisenda Eixarch, Carla Domínguez-Gallardo, Toni Galmes, Marta Muniesa, Maria Julia Zanini, Alan Domínguez, Marta Cirach, Mark Nieuwenhuijsen, Xavier Basagaña, Xavier Querol, Maria Foraster, Mariona Bustamante, Jesus Pujol, Mireia Gascon, Elisa Llurba, María Dolores Gómez-Roig, Payam Dadvand, Jordi Sunyer

**Affiliations:** a Barcelona Institute for Global Health, ISGlobal, Barcelona, Spain; b Universitat Pompeu Fabra, Barcelona, Spain; c Facultat de Medicina i Ciències de la Salut, Universitat de Barcelona, Barcelona, Spain; d Spanish Consortium for Research on Epidemiology and Public Health, Madrid, Spain; e BCNatal, Barcelona Center for Maternal Fetal and Neonatal Medicine, Hospital Sant Joan de Déu and Hospital Clínic, University of Barcelona, Barcelona, Spain; f Institut d’Investigacions Biomediques August Pi i Sunyer and Centre for Biomedical Research on Rare Diseases, Barcelona, Spain; g Department of Surgery and Medical-Surgical Specialities, School of Medicine and Health Sciences, Universitat de Barcelona, Barcelona, Spain; h Department of Obstetrics, Institut de Recerca and Hospital Santa Creu i Sant Pau, Barcelona, Spain; i Primary Care Interventions to Prevent Maternal and Child Chronic Diseases of Perinatal and Developmental Origin Network, RD21/0012/0001, Instituto de Salud Carlos III, Madrid, Spain; j Primary Care Interventions to Prevent Maternal and Child Chronic Diseases of Perinatal and Developmental Origin Network, RD21/0012/0003, Instituto de Salud Carlos III, Madrid, Spain; k Institut de Recerca Sant Joan de Deu, Barcelona, Spain; l Institute of Environmental Assessment and Water Research, Spanish Council for Scientific Research, Barcelona, Spain; m PHAGEX Research Group, Blanquerna School of Health Science, Universitat Ramon Llull, Barcelona, Spain; n MRI Research Unit, Department of Radiology, Hospital del Mar, Barcelona, Spain; o Unitat de Suport a la Recerca de la Catalunya Central, Fundació Institut Universitari per a la Recerca a l’Atenció Primària de Salut Jordi Gol i Gurina, Manresa, Spain; p IMIM, Hospital del Mar Medical Research Institute, Barcelona, Spain

## Abstract

**Background:**

There is a scarcity of evidence of the influence of exposure to air pollution during pregnancy on the human fetal brain characterised prenatally. We aimed to evaluate the association of exposure to air pollution with fetal brain morphology.

**Methods:**

In this prospective cohort study, we used data from the Barcelona Life Study Cohort, Spain, which recruited 1080 pregnant women at 8–14 weeks of gestation between Oct 16, 2018, and April 14, 2021, from three major university hospitals in Barcelona. Eligible participants were aged 18–45 years, had a singleton pregnancy, and had a fetus without major congenital anomalies. Third-trimester transvaginal neurosonography was applied to evaluate fetal brain morphological development. We integrated comprehensive data on time–activity patterns with land use regression, dispersion, and hybrid models to estimate exposure to NO_2_, PM_2·5_, and black carbon at home, workplace, and commuting routes during pregnancy until the neurosonography date. Single-pollutant linear mixed regression models and multipollutant ridge regression models were applied to estimate the associations between air pollutants and fetal brain outcomes, controlled for confounders. Distributed lag linear models were used to identify the vulnerable windows.

**Findings:**

Among 1080 participants recruited at baseline, 954 attended the follow-up for the neurosonographic examination, 754 of whom were included in this study. In single-pollutant models, we found that prenatal exposure to NO_2_, PM_2·5_, and black carbon was associated with a wider anterior horn of lateral ventricles, wider cisterna magna, and larger cerebellar vermis. We also observed that higher exposure to black carbon was related to a shallower Sylvian fissure. No clear pattern or associations were observed between air pollution and other structures of brain morphology. Multipollutant models showed that these associations with black carbon remained significant, whereas associations with PM_2·5_ and NO_2_ lost significance for some indicators. A potential vulnerability window in mid-to-late pregnancy was identified for these associations.

**Interpretation:**

Exposure to air pollution might affect brain morphological development as early as the fetal stage. Our findings could have important policy implications as they highlight the need to mitigate exposure of pregnant individuals to air pollution in urban areas to protect fetal brain development.

**Funding:**

European Research Council.

## Introduction

The developmental potential of the brain requires several essential stages that should be accomplished sequentially, principally during fetal and early life. During these periods, the brain undergoes rapid development to form the foundational structure and circuity.^1^ As such, these periods are considered windows of particular vulnerability to the influence of environmental factors on brain development due to its immature state.^2^ According to the developmental origins of health and disease concept, environmental stressors could result in brain structural and functional changes in the developing fetus, which might have profound effects on brain development. Such changes, among others, could increase the risk of neurobehavioural conditions and cognitive disorders later in life. Notably, the earlier these disruptions occur, the more severe their potential effect on later developmental stages might be.^3^ Thus, understanding the potential effects of environmental stressors on the fetal brain is pivotal for promoting brain and neurological health.

Air pollution has various adverse effects on human health, including impairing fetal growth and development. The placenta, serving as the interface between maternal and fetal circulation, plays a vital role in supporting and regulating fetal development. Air pollutants might either directly penetrate the placenta into fetal circulation and the brain,^4^ or indirectly disrupt the placental function and overactivate the maternal immune system.^5^, ^6^ In turn, these effects could lead to fetal neuroinflammation, oxidative stress, and epigenetic modifications, potentially affecting fetal brain development. Previous evidence strongly suggests that exposure to air pollution is adversely associated with brain development and a series of neurological consequences in both human epidemiological and animal experiment studies.^7^, ^8^ However, the potential effect of prenatal exposure to air pollution on the morphological development of the fetal brain has not been studied. Animal models showed prenatal exposure to air pollution could increase the cortical volume of embryonic brains, which switched to a decreased volume during the postnatal period,^9^ and could produce ventriculomegaly in the postnatal brain in mice.^10^ To date, only one study in humans has investigated how prenatal air pollution exposure can affect neonatal brain morphometry, reporting a significant association between higher exposure levels and larger relative volumes of the ventricles and cerebellum.^11^ Other studies have investigated how exposure to air pollution during pregnancy or childhood is associated with brain morphological alterations among children and adolescents and have reported inconsistent results.^12^, ^13^, ^14^ Additionally, the vast majority of these studies that evaluated exposure to air pollution relied on the estimated ambient levels of air pollution at the maternal residential address, overlooking the difference between ambient and indoor levels of air pollutants and also that personal exposure could occur in other microenvironments such as the workplace and commuting routes.

**Research in context**

**Evidence before this study**

Exposure to air pollution has been associated with abnormal brain and neural development but, to our knowledge, all the previous studies estimated the association of air pollution with postnatal brain development. We searched PubMed and Web of Science from database inception to May 10, 2024, without language restrictions, using a combination of keywords: air pollution (“air pollut*” OR “air contaminate*” OR “particulate matter” OR “PM” OR “nitrogen oxides” OR “NO” OR “black carbon”) and brain morphology (“brain development” OR “brain structure” OR “brain morphology” OR “brain change” OR “neuroimage*” OR “brain imag*” OR “MRI” OR “magnetic resonance imaging” OR “neurosonography” OR “brain ultrasound” OR “brain anatomy” OR “brain sonography”). We found that the evidence on the association between exposure to air pollution and brain structure and function has been accumulating in recent years. However, there is no direct evidence assessing the association between air pollutants and the human fetal brain. One animal study reported that diesel exhaust particle exposure during pregnancy might affect the morphological development of the mouse embryonic brain. In humans, most of these studies have been conducted in children, whereas a substantial part of brain development occurs during the fetal period, making it an important window of exposure. We identified only one recent, relevant epidemiological study from the UK that found exposure to particulate matter during pregnancy was associated with structural changes in the neonatal brain. This study reinforced the need for further investigation into the effect of prenatal exposure to air pollution on early neurodevelopment. Additionally, a majority of studies that assessed the influence of exposure to air pollution during pregnancy on brain health relied on ambient levels of air pollutants at home, overlooking the contribution routes to personal exposure of other microenvironments such as the workplace and commuting.

**Added value of this study**

This study focused on the associations of prenatal exposure to air pollution with fetal brain morphological development. We evaluated fetal brain cortical folding (including depth of the insula, Sylvian fissures, and parieto–occipital, cingulate, and calcarine sulci), cerebrospinal fluid (CSF) spaces (including the width of anterior and posterior lateral ventricles, third ventricle, and cisterna magna), corpus callosum length, cerebellar transverse diameter, and vermis height using neurosonography. We applied three modelling approaches to comprehensively assess exposure to NO_2_, PM_2·5_, and black carbon at the home, workplace, and commuting routes. We identified that prenatal exposure to air pollutants was associated with a reduction in the cortical maturation of the fetal brain and a complementary increase in CSF space and a larger cerebellum volume. Our results suggest that air pollution is associated with different brain morphological development as early as the fetal stage.

**Implications of all the available evidence**

An emerging body of evidence has raised concerns regarding the adverse effects of air pollution on brain development. We found that prenatal exposure to air pollution could influence the brain morphology of the developing fetus. Combining our findings with those of previous studies suggests that air pollution might damage the brain in the fetus, with consequences throughout the life course. Given the considerable burden of neurodevelopmental and mental health conditions, these findings highlight the need for policies and interventions to protect the next generations of people from the adverse effects of air pollution.

We aimed to evaluate the association of prenatal exposure to air pollution during pregnancy with fetal brain morphological development.

## Methods

### Study design and participants

This prospective cohort study used data from the Barcelona Life Study Cohort (BiSC), Spain, which has been described elsewhere.^15^ Briefly, the cohort recruited 1080 pregnant women during their first trimester (weeks 8–14 of gestation) between Oct 16, 2018, and April 14, 2021, from three major university hospitals in Barcelona: Hospital Sant Joan de Déu, Hospital Clínic, and Hospital Santa Creu i Sant Pau. Eligible participants in BiSC were aged 18–45 years, had a singleton pregnancy, lived in the catchment area of recruiting hospitals, were able to communicate in Catalan, Spanish, or English, and had a fetus without major congenital anomalies (ie, malformations incompatible with life, such as anencephaly, and holoprosencephaly). The study was approved by the corresponding ethics committees in all the participating institutions and hospitals (Clinical Research Ethics Committee of the Parc de Salut Mar [2018/8050/I], Medical Research Committee of the Fundació de Gestió Sanitària del Hospital de la Santa Creu i Sant Pau de Barcelona [EC/18/206/5272], and Ethics Committee of the Fundació Sant Joan de Déu [PIC-27-18]). Participants provided written informed consent before being included in the study. Sex data for both pregnant individuals and fetuses were obtained from medical records, and ethnicity data were collected through participant self-report.

### Outcome assessment

Detailed two-dimensional neurosonographical examination of the fetal brain was done by trained obstetricians in the third trimester (222·0 days ± 9·0). To standardise the ultrasound measurements, obstetricians attended an intensive training course to review and practise fetal neurosonography based on the standard operating procedure that we developed for these measurements according to the International Society of Ultrasound in Obstetrics and Gynecology guidelines.^16^, ^17^ For each participant, all the measurements for the neurosonography were carried out by the same obstetrician and using the same ultrasonography equipment in each hospital (appendix p 4). The morphological images of the fetal brain were acquired through transvaginal ultrasonography.

Fetal brain morphological structures were measured by two raters (MM and MJZ) following different plane references as described in the appendix (p 4). Before evaluations, the two raters attended another training session focused on refining the evaluation procedures to ensure consistency. We measured the depth of the insula, Sylvian fissures, parieto–occipital, cingulate, and calcarine sulci to assess the degree of cortical folding and the width of anterior and posterior horns of lateral ventricles, third ventricle, and cisterna magna to evaluate cerebrospinal fluid (CSF) spaces. We also measured corpus callosum length, cerebellar transverse diameter, and vermis height. The reproducibility of these measurements was assessed using the inter-rater intraclass correlation coefficient, showing moderate to good reproducibility for all the measurements (appendix p 5).

### Exposure assessment

We assessed maternal exposure to NO_2_, PM_2·5_, and black carbon using three different types of models: land use regression (LUR) models, dispersion models, and hybrid LUR–dispersion models. The details of these models have been described elsewhere.^18^ Briefly, we conducted four air pollution monitoring campaigns (2021–22) in 34 representative locations across our study region (appendix p 6) following the guidelines established by the European Study of Cohorts for Air Pollution Effects^19^, ^20^ to develop the LUR model. The ADMS-Urban software (Cambridge Environmental Research Consultants, Cambridge, UK), which is based on a Gaussian dispersion model with integrated photochemical reaction models, street canyon models, and a meteorological preprocessing model, was used to develop the dispersion model to estimate hourly air pollution concentrations.^21^ The hybrid model combined dispersion model estimates with the potential predictors from the LUR model together with routine air pollution monitoring data and meteorological data through a random forest algorithm to estimate the levels of each pollutant. The adjusted coefficients of determination (*R*^2^) of these models are shown in the appendix (pp 6–7). The hybrid model with the better performance (higher *R*^2^) of each air pollutant was selected as the main model.

We calculated mean concentrations of air pollutants during pregnancy for home, workplace, and commuting routes between these two (except in the hybrid model) until the neurosonography date. We then developed an index of total ambient personal exposure weighted by the time that each participant spent in each of these microenvironments (ie, home, workplace, and commuting routes). Data on time–activity patterns were collected using a smartphone with a validated geolocation application (ExpoApp, Ateknea Solutions, Barcelona, Spain)^22^ and asking participants to characterise the time spent in each microenvironment (appendix pp 8–10).

### Statistical analysis

Our calculation before the start of the study estimated that a sample size of 300 participants was sufficient to achieve 80% statistical power at a 5% significance level to detect our expected associations.

We first applied multiple imputations by chained equations to impute missing values of confounders in the main model to prevent loss of information (appendix p 11). The raw values of the brain morphological structures were natural log-transformed for use in the subsequent analyses. Linear mixed effects regression models with the rater and hospital as random effects were developed to estimate the associations of exposure levels of each pollutant with each of the neurosonography structures of fetal brain morphology (one at a time). We also used multipollutant models using ridge regression to account for co-exposure to NO_2_, PM_2·5_, and black carbon in association with fetal brain morphological development.^23^ According to the directed acrylic graph developed based on available evidence (appendix p 12), the main models were adjusted for maternal education, children's ethnicity, active and passive smoking during pregnancy (at least once), alcohol consumption during pregnancy (at least once), parity, fetal sex, and gestational age at the time of neurosonographic examination (days). The associations were presented as percentage differences in the brain morphological structures associated with an IQR increase in air pollutants calculated as 100 × [exp(regression coefficient for air pollutant) –1] with its corresponding 95% CI.

We applied distributed lag linear models to identify the vulnerable windows for exposure to air pollution in association with each fetal brain morphological indicator. We included in the model the weekly exposure estimated at weeks 1–30 of pregnancy. We used natural splines for the lagged associations and equidistant knots in the lag space.^24^ We tested spline degrees of freedom ranging from 2 to 6, selecting the one that minimised the Akaike information criterion of the model.^24^ The models in these analyses were adjusted for the same covariates as the main analysis.

We conducted a series of sensitivity analyses to test the robustness of our findings. First, we did a complete case analysis to identify any effect related to missing data. Second, we further adjusted our analyses for: (1) maternal pre-pregnancy thyroid pathology collected by midwives in the first visit;^25^ (2) estimated fetal weight calculated at the time of the neurosonography examination, using Hadlock's formula; (3) maternal BMI based on measurements taken during the first trimester and calculated as kg/m^2^; and (4) maternal age at the time of neurosonographic examination (based on the maternal date of birth). Third, considering that prenatal complications such as intrauterine growth retardation, pre-eclampsia, gestational diabetes, and gestational hypertension might affect the fetal brain morphological development,^26^, ^27^ we further adjusted these factors and did the main analyses again after removing the participants who had been diagnosed with one of the prenatal complications. Fourth, we additionally adjusted the main analysis for the ambient temperature, which might be a potential risk factor for brain development.^28^ Daily mean ambient temperature was assessed by random forest models,^29^ weighted by the time that participants spent at home and in the workplace. Fifth, we repeated the main analyses after correcting the brain morphology structures by biparietal diameter to standardise these structures by fetal head size. Sixth, to disentangle the associations for each microenvironment, we separately investigated the associations of air pollution at home and in the workplace with fetal brain morphological development. Seventh, we applied LUR and dispersion models as alternative methods for exposure assessment and repeated the main analyses using these two sets of exposure. Eighth, we measured the left and right of the anterior horn of the lateral ventricles for each fetus and applied the mean value in the main model. However, for technical reasons, we only measured either the left or right of the posterior horn of the lateral ventricles from the distal hemisphere following the standard plane for each fetus. Considering the asymmetry of the ventricles, we separately evaluated the association of exposure to air pollutants with the left and right anterior and posterior horns of the lateral ventricles. Ninth, to evaluate the potential effect of tobacco smoke exposure on our analyses, we repeated the main models after separately excluding participants with active and passive smoking exposure during pregnancy. Finally, given the relatively large number of comparisons, we adjusted our p values for multiple comparisons using the method developed by Benjamini and Hochberg.^30^ Based on this method, we estimated the adjusted p values for the main analysis: NO_2_, PM_2·5_, and black carbon across total ambient personal exposure assessed by the hybrid model combined with 12 main outcomes, resulting in a total of 36 tests.

We evaluated the modification of our assessed associations by fetal sex. We first tested the statistical significance of the interaction term between fetal sex and air pollution exposure in the main models using the likelihood-ratio test. We then stratified the analyses based on fetal sex.

All the analyses and steps were done in R software (version 4.2.2). We considered a two-tailed p value of less than 0·05 as statistically significant.

### Role of the funding source

The funder of the study had no role in the study design, data collection, data analysis, data interpretation, or writing of the report.

## Results

Among 1080 participants recruited at baseline, 954 attended the follow-up for the neurosonographic examination. After excluding 177 fetuses that were not in cephalic position or whose mothers did not consent to the transvaginal ultrasound and 23 fetuses with poor quality neurosonography data, 754 were included in this study. The socioeconomic, demographic, and lifestyle characteristics of the participants included in our study were similar to those of 1080 BiSC participants at baseline (appendix p 14). The sociodemographic, lifestyle, and clinical characteristics of the included 754 participants are shown in table 1. The characteristics of fetal brain morphology and air pollution exposure levels are shown in table 2. Overall, NO_2_, PM_2·5_, and black carbon assessed by different models correlated moderately to highly with each other (*r*_s_ 0·50–0·94; appendix p 15).

**Table 1:** Socioeconomic, demographic, and lifestyle characteristics

		**All participants (n=754)**
Fetal sex		
	Female	389 (52%)
	Male	365 (48%)
Maternal parity		
	Multiparous	323 (43%)
	Nulliparous	431 (57%)
Maternal educational level		
	With university degree	517 (69%)
	Without university degree	237 (31%)
Fetal ethnicity		
	European White	512 (68%)
	Other ethnicity*	242 (32%)
Active smoking during pregnancy		
	No	668 (89%)
	Yes	59 (8%)
	Missing data	27 (4%)
Passive smoking during pregnancy		
	No	408 (54%)
	Yes	316 (42%)
	Missing data	30 (4%)
Alcohol consumption during pregnancy		
	No	507 (67%)
	Yes	212 (28%)
	Missing data	35 (5%)
Maternal pre-pregnancy thyroid pathology		
	No	688 (91%)
	Yes	61 (8%)
	Missing data	5 (1%)
Intrauterine growth retardation		
	No	676 (90%)
	Yes	26 (3%)
	Missing data	52 (7%)
Pre-eclampsia		
	No	676 (90%)
	Yes	26 (3%)
	Missing data	52 (7%)
Gestational diabetes		
	No	665 (88%)
	Yes	37 (5%)
	Missing data	52 (7%)
Gestational hypertension		
	No	673 (89%)
	Yes	29 (4%)
	Missing data	52 (7%)
Prenatal complications		
	No	671 (89%)
	Yes	83 (11%)
Hospital of follow-up		
	Hospital Clínic	58 (8%)
	Hospital Sant Joan de Déu	325 (43%)
	Hospital Santa Creu i Sant Pau	371 (49%)
Gestational age at ultrasound, days		223·0 (9·0)
Estimated fetal weight, g		1898·0 (1724·0–2078·5)
Maternal BMI, kg/m^2^		23·5 (21·33–26·19)
Maternal age at ultrasound, years		34·0 (31·0–37·0)
Biparietal diameter at ultrasound, mm		80·0 (78·0–83·0)

Data are n (%) or median (IQR).

* Other ethnicities included Arabian (including north Africa and the Middle East), sub-Saharan African, Latin American, south Asian (including Pakistan), Iranian, European Black, European Arabian, European east Asian, European Iranian, European Latin American, European South Asian, and other.

**Table 2:** Fetal neurosonographic structures and the estimated total ambient personal exposure to air pollution

		**Number of samples**	**Median (IQR)**
Brain morphological structures*			
Cortical folding			
	Insula depth, mm	675	25·6 (24·7–26·7)
	Sylvian fissure depth, mm	675	12·9 (11·8–14·5)
	Parieto–occipital sulcus depth, mm	551	12·4 (11·2–13·8)
	Cingulate sulcus depth, mm	572	6·6 (5·7–7·7)
	Calcarine sulcus depth, mm	459	12·1 (10·1–13·3)
Cerebrospinal fluid spaces			
	Anterior lateral ventricles width, mm	473	1·9 (1·5–2·3)
	Posterior lateral ventricles width, mm	659	4·0 (3·1–5·0)
	Third ventricle width, mm	683	1·0 (0·8–1·2)
	Cisterna magna width, mm	614	6·6 (5·4–7·7)
Others			
	Corpus callosum length, mm	674	41·6 (40·2–43·3)
	Cerebellar vermis height, mm	647	19·6 (18·5–20·6)
	Transverse cerebellar diameter, mm	628	40·3 (38·7–42·4)
**Air pollution**			
Land use regression models			
	NO_2_, μg/m^3^	754	38·1 (30·8–47·1)
	PM_2·5_, μg/m^3^	754	17·0 (14·7–19·9)
	Black carbon, μg/m^3^	754	1·4 (1·2–1·8)
Dispersion models			
	NO_2_, μg/m^3^	752	28·7 (24·3–34·7)
	PM_2·5_, μg/m^3^	752	12·4 (10·8–14·5)
	Black carbon, μg/m^3^	752	0·8 (0·6–1·1)
Hybrid models			
	NO_2_, μg/m^3^	752	37·2 (32·7–43·0)
	PM_2·5_, μg/m^3^	752	12·6 (11·9–13·3)
	Black carbon, μg/m^3^	752	1·2 (1·1–1·3)

* The different sample sizes between brain morphological structures are due to measurements for each brain morphological indicator being taken from specific ultrasound sections of the fetal brain in line with clinical guidelines. When these sections could not be obtained due to factors such as fetal position or limitations in maternal tissue transmission during the scan, the sample could not obtain the corresponding value of structures.

**Figure 1:**
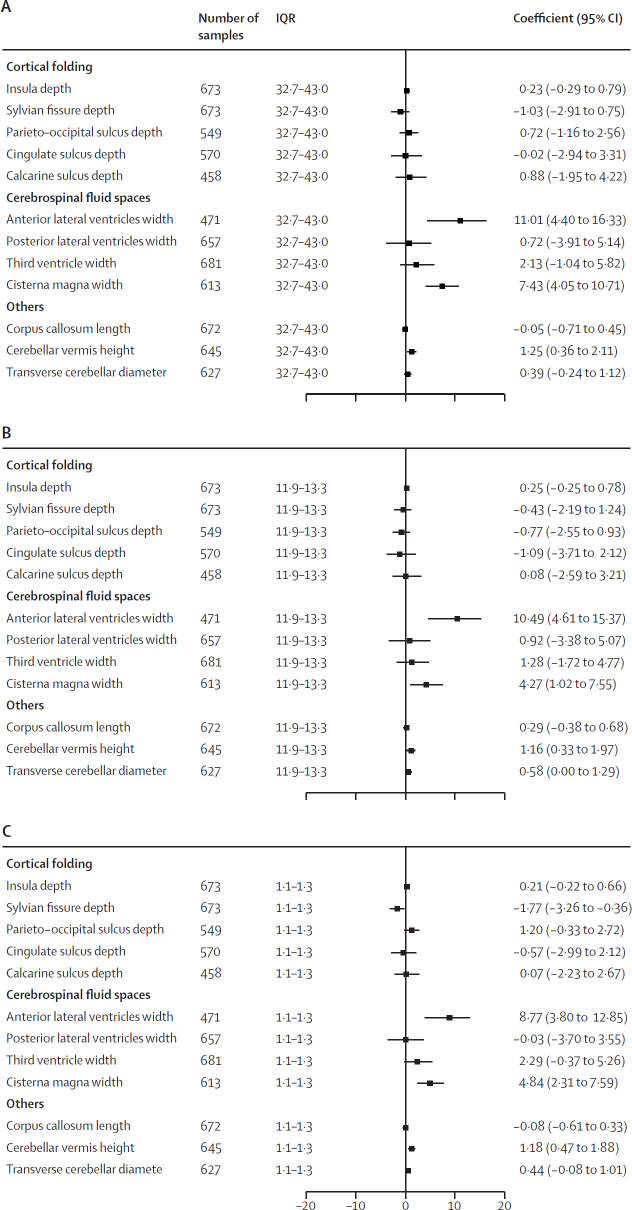
Adjusted percentage difference in brain morphological structures associated with one IQR increase in exposure to ambient pollutants (A) Exposure to NO_2_. (B) Exposure to PM_2·5_. (C) Exposure to black carbon. Percentage differences are adjusted for fetal sex (female *vs* male), parity (multiparous *vs* nulliparous), maternal education (with university degree *vs* without university degree), ethnicity (White European *vs* other), active smoking during pregnancy (no *vs* yes), passive smoking during pregnancy (no *vs* yes), alcohol consumption during pregnancy (no *vs* yes), and gestational age at ultrasound (days), and the rater and hospital as random effects. All outcomes were measured in mm and natural log-transformed for analysis.

With respect to the structures of cortical folding, we found that each IQR increase in black carbon was significantly associated with a 1·77% (95% CI –3·26 to –0·36) decrease in Sylvian fissure depth (figure 1). No statistically significant relationships were detected between exposure to air pollution and the remaining structures of cortical maturity.

With regard to CSF spaces, exposure to higher levels of NO_2_, PM_2·5_, and black carbon was significantly associated with enlargement of the anterior horn of the lateral ventricles and the cisterna magna (figure 1). We did not find any significant association between air pollution exposure and the width of the posterior horn of lateral ventricles and the third ventricle.

We found that higher exposure to NO_2_, PM_2·5_, and black carbon was significantly associated with increased cerebellar vermis height (figure 1). We also observed that higher exposure to PM_2·5_ was significantly associated with larger transverse cerebellar diameter. No significant association was detected between air pollution and length of the corpus callosum.

**Figure 2:**
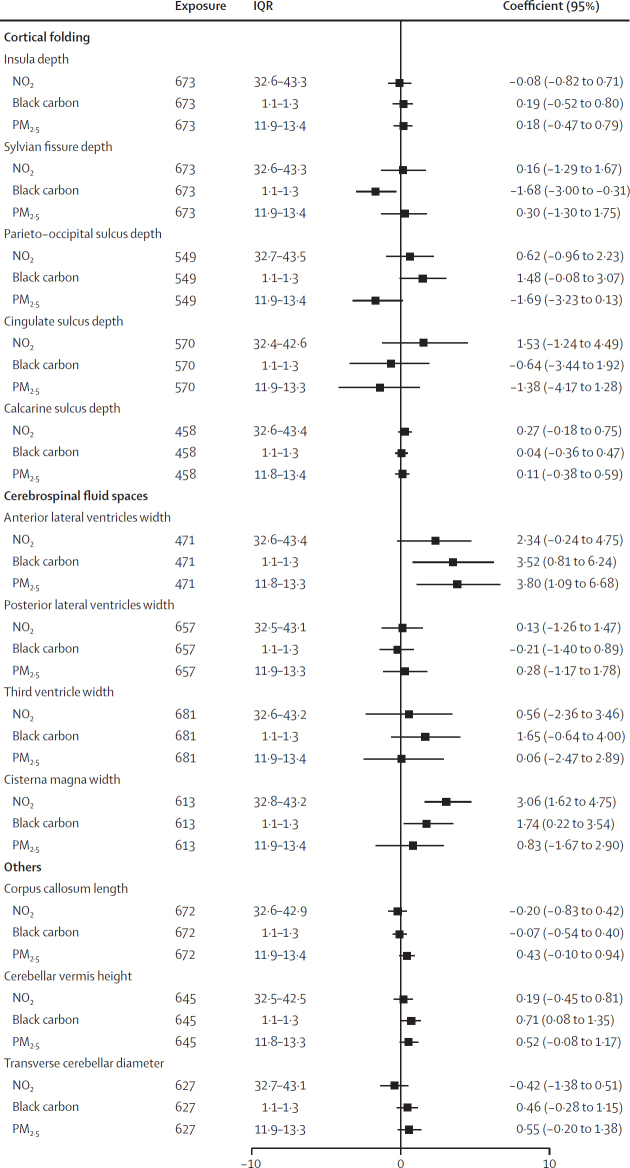
Adjusted percentage difference in brain morphological structures associated with one IQR increase in exposure to NO_2_, PM_2·5_, and black carbon in the multipollutant models Percentage difference are adjusted for fetal sex (female *vs* male), parity (multiparous *vs* nulliparous), maternal education (with university degree *vs* without university degree), ethnicity (White European *vs* other), active smoking during pregnancy (no *vs* yes), passive smoking during pregnancy (no *vs* yes), alcohol consumption during pregnancy (no *vs* yes), and gestational age at ultrasound (days), and the rater and hospital as random effects.

In multipollutant models, we generally observed similar patterns to the single-pollutant models in terms of the direction and strength of associations, especially for black carbon (figure 2). However, the associations of NO_2_ with anterior lateral ventricles width and of PM_2·5_ with cerebellar vermis height and cisterna magna lost their statistical significance.

A potential window of susceptibility was identified for exposure to air pollutants and wider anterior horn of lateral ventricles and higher cerebellar vermis in the second trimester of pregnancy (appendix pp 31–33, 46–48). We also observed significant direct associations of NO_2_ and black carbon with cisterna magna width after weeks 25–30 of pregnancy (appendix pp 40–42). We did not observe any specific evidence to suggest potentially vulnerable windows for associations between air pollution and the remaining structures of brain morphological development.

Sensitivity analyses were generally in line with the results of the main analysis, apart from a few exceptions (appendix pp 52–90). After removing the participants with prenatal complications, for black carbon, the association with transverse cerebellar diameter gained statistical significance, whereas the Sylvian fissure depth lost statistical significance. When we separately conducted the analyses for workplace exposure, we found that exposure to NO_2_ was significantly associated with shallower Sylvian fissure depth and exposure to PM_2·5_ was significantly associated with cingulate sulcus depth, but the associations of exposure to black carbon with Sylvian fissure depth and cisterna magna width lost statistical significance (appendix pp 72–77). Moreover, when we used the LUR and dispersion models as alternative methods to assess air pollution, we observed that NO_2_ and PM_2·5_ were also significantly associated with shallower Sylvian fissure depth, and PM_2·5_ was related to greater insula depth in LUR models (appendix pp 78–81). When participants exposed to passive smoking during pregnancy (n=316 [42%]) were removed, the strength of associations between exposure to PM_2·5_ and both cisterna magna width and transverse cerebellar diameter remained similar to the main analyses; however, these associations were no longer significant (appendix pp 84–87). Finally, after adjustment of the p values of our analyses for multiple comparisons, the association of exposure to black carbon with Sylvian fissure depth lost significance (p=0·065), as did the association of exposure to PM_2·5_ with transverse cerebellar diameter (p=0·22; appendix pp 88–90).

In the sex-stratified analysis, although some associations appeared to be stronger in females than males, we did not observe any significant interactions between fetal sex and exposure to NO_2_, PM_2·5_, or black carbon in the association with fetal brain morphological indicators (all interaction p>0·05; appendix pp 91–96).

## Discussion

This study investigated the association of prenatal exposure to air pollution with fetal brain morphology. Our findings suggest that prenatal exposure to air pollution was associated with different fetal brain morphological development patterns, particularly in mid-to-late pregnancy. Overall, these associations could be summarised as a reduction in the degree of fetal brain cortical folding and an increase in non-parenchymatous volumes (CSF space) and particular brain structures (cerebellum).

Cortical folding allows the smooth surface of the brain to evolve into the sulci and gyri system, which is closely related to brain function and cognition.^31^ Our results suggest a shallower Sylvian fissure and greater insula depth in association with increased exposure to air pollution. The opposite associations of air pollution with Sylvian fissure and insula depth might be partly because the development of the Sylvian fissure and insular cortex are highly interdependent, with the expansion of the telencephalon gradually burying the insula beneath the Sylvian fissure as cortex development proceeds. Our findings are in line with previous studies on fetal brain morphology in cases of intrauterine growth restriction and pre-eclampsia, which also observed a reduction in Sylvian fissure depth accompanied by an increase in insula depth, with no significant associations found for other cortical development measures.^26^, ^27^ We conducted a sensitivity analysis adjusting for and removing the effects of these prenatal complications and the results remained consistent with our main findings, suggesting that the observed associations between air pollution and outcomes of fetal brain morphology were independent of these complications.

Regarding CSF space, one of its basic functions is to dynamically equilibrate the pressure fluctuations caused by brain tissue, blood, and CSF. We found that higher exposure to air pollution during pregnancy was significantly associated with a wider anterior horn of lateral ventricles and cisterna magna. In line with our findings, a study, published in 2023, done in neonates showed that higher exposure to PM_10_ was related to larger relative CSF volume.^11^ Another animal study conducted in mice found that prenatal exposure to air pollution was related to neonate ventriculomegaly.^10^ Additionally, fetuses with enlarged cisterna magna have been shown to be more likely to have lower adapting and gross motor ability in childhood.^32^

We found that prenatal exposure to air pollution was associated with larger transverse cerebellar diameter and cerebellar vermis. The results of previous epidemiological studies evaluating the association between air pollution exposure and cerebellum are heterogeneous. Consistent with our findings, Bos and colleagues^11^ and Guxens and colleagues^13^ found prenatal exposure to higher air pollution concentration was associated with a larger relative size of the cerebellum in neonates and children, respectively. Contrary to our results, a longitudinal study observed that exposure to air pollution during childhood was related to smaller volumes of cerebellum and cerebellar vermis in children aged 12 years.^14^ Another study reported that exposure to particulate air pollution during pregnancy was associated with larger cerebellum volume in their single-pollutant model, whereas in their multipollutant model, higher exposure to PM_2·5_ was associated with smaller cerebellum volume.^13^ Given that the cerebellum and cerebellar vermis develop rapidly during the first 2 years of life and begin to decrease in volume in the pre-adolescent period,^33^ the effects of exposures on them might have specific sensitivity windows.^13^ Thus, the inconsistent results might be, in part, due to differences in periods of exposure measured and in the populations assessed in these studies.

The multipollutant models can provide more insight into real-world associations between exposures and outcomes. Our results showed that the associations between exposure to black carbon and fetal brain morphology remained robust after adjustment for PM_2·5_ and NO_2_, suggesting that black carbon might influence fetal brain development independently of these co-pollutants. Conversely, the loss of statistical significance in the associations of NO_2_ and PM_2·5_ with specific structures of brain morphology might indicate shared sources, such as fossil fuel combustion, or overlapping pathways of biological effect. Similarly, a Canadian study on prenatal air pollution exposure found the association between PM_2·5_ and autism spectrum disorder lost its statistical significance in multipollution models, whereas the association between NO_2_ and autism spectrum disorder remained significant.^34^ With respect to the potential windows of susceptibility, previous studies in children found that exposure to air pollution in mid-to-late pregnancy was associated with poorer neurodevelopment.^35^, ^36^ These findings are consistent with our findings indicating two windows of susceptibility, one in the second trimester and one between weeks 25 and 30 of pregnancy. During mid-to-late pregnancy, the brain undergoes structural maturation, such as rapid neuronal growth, migration, synapse formation, and myelination,^37^ which might make it highly sensitive to environmental turbulence.

Prenatal smoking exposure, a well established risk factor for impairing brain and neurodevelopment,^38^ might have influenced our analyses. Nevertheless, the exclusion of both participants with active and passive smoking during pregnancy, in general, did not considerably change our results. In our study, we did not find any significant interaction between fetal sex and prenatal exposure to air pollutants in association with fetal brain morphological development. In line with our results, two previous studies conducted in neonates and children also did not find evidence of a sex-specific association between air pollution and brain morphology.^11^, ^13^

Although the potential pathways underlying the association of prenatal exposure to air pollution with brain development are yet to be established, previous animal models and experiment studies support several plausible mechanisms. Exposure to air pollution can induce placental damage, which, in turn, can result in reduced placental perfusion and inadequate availability of nutrients and oxygen to the fetus,^5^ which might disrupt the regular trajectory of fetal brain development. Moreover, ambient particles could directly penetrate the placenta and distribute to the fetal brain, which might produce oxidative stress and neuroinflammation.^4^ Air pollutants can also trigger maternal immune activation (eg, antigen-specific T-cell and B-cell responses), which could induce maternal excessive inflammatory responses thereby affecting fetal brain development.^39^ Furthermore, an animal study showed that maternal exposure to particulate matter could dysregulate the pathway related to neuronal survival and apoptosis in the fetal brain.^40^

A major strength of our study is its reliance on a large amount of prospectively collected data in a birth cohort with detailed neurosonography taken during pregnancy. A further strength is its comprehensive exposure assessment that included exposure to all relevant microenvironments for pregnant women. Moreover, in addition to the commonly used LUR model in previous epidemiological studies on this topic, we also applied dispersion models and hybrid models to assess exposure to air pollution.

Our study also has limitations. One limitation is the uncertainty of the assessment of exposure to air pollution. Our reliance on estimates of spatiotemporal models as surrogates of true personal exposure to air pollutants might have led to some degree of exposure measurement error, given that the model performance (*R*^2^) for NO_2_ and PM_2·5_ was not optimal. Moreover, the temporal adjustment of our LUR model estimates assumed that temporal variability in one station was representative of the variability across all the study areas. Such an assumption could have introduced measurement errors in our exposure estimates. These exposure measurement errors could have influenced our association estimates; however, the consistency of findings across three different modelling frameworks could provide some degree of confidence in our findings. Second, a slightly higher proportion of the BiSC participants (69%) had a university degree compared with the general population of women (aged 20–44 years) residing in Barcelona (64·2% in 2019).^41^ This difference could have influenced the generalisability of our findings. Third, intra-rater reliability for accurate manual tracing of each brain morphological indicator was not established by either rater; moreover, we randomly selected only a subsample of 30 participants to evaluate reproducibility. Although this method is consistent with common practice and designed to balance feasibility with statistical robustness, the small sample size might have limited the generalisability of our results to the entire cohort. Additionally, errors in outcome measurement might have occurred in this study due to non-optimal reliability and validity of our measurements. However, this error was likely to be of the classic and non-differential type, which tends to attenuate associations towards null rather than introduce bias.^42^, ^43^ Although this limitation could have diluted our associations, our rigorous training process and incorporation of rater as a random effect in our main modelling might have minimised such an influence. Fourth, although we did not find any strong evidence for a confounding role of self-reported active or passive smoking in our evaluated associations, the lack of a biological marker such as cotinine meant that we could not completely rule out the potential residual confounding by smoking. Fifth, the data on active smoking, passive smoking, and alcohol consumption were collected by a self-administered questionnaire and only collected the categorical answer (yes or no) rather than the dose, which could have resulted in the misclassification of these variables. Finally, our study included a large number of analyses. Instead of adjusting for multiple comparisons,^44^ our approach emphasised the consistency of results across different association estimates for the same pollutant and also across different pollutants.

In conclusion, our findings suggest that prenatal exposure to NO_2_, PM_2·5_, and black carbon might alter fetal brain morphology, including a reduced degree of cortical folding, an increase in CSF space, and a larger cerebellum volume, especially in mid-to-late pregnancy. The alteration of fetal brain morphology could have a lasting influence on subsequent brain development and hence could potentially increase susceptibility to neurodevelopmental disorders later in life. Therefore, neurodevelopmental follow-up of the children born from BiSC is needed to investigate whether the observed changes in fetal brain morphology affect later neurodevelopment. If confirmed by BiSC follow-up and other future studies, our results could have important implications for policy makers, highlighting the need to develop policies and implement effective interventions to protect pregnant individuals and future generations from the adverse effects of air pollution. Future epidemiological and experimental studies with more accurate exposure and outcome measurements are needed to confirm our findings in other settings and to explore possible mechanisms underlying these associations.

### Contributors

### Data sharing

Authors will share the data after the approval of the request by the BiSC steering committee and signing the data transfer agreement with the requesting institution. The request forms are available on the cohort website (https://projectebisc.org/en/home/).

## Declaration of interests

We declare no competing interests.
