# Supplementary material for: Air pollution and fetal brain morphological development: a prospective cohort study
Source: Lancet Planet Health. 2025 Jun 11;9(6):e480–90. doi: 10.1016/S2542-5196(25)00093-2 (PMC12167762; doi:10.1016/S2542-5196(25)00093-2)
Supplement: Supplementary appendix [file mmc1.pdf]

THE LANCET
Planetary Health

Supplementary appendix

This appendix formed part of the original submission and has been peer reviewed. We post it as supplied by the authors.

Supplement to: Gómez-Herrera L, Zhao Y, Rivas I, et al. Air pollution and fetal brain morphological development: a prospective cohort study. *Lancet Planet Health* 2025; **9:** e480–90.

Supplemental Materials

Air pollution and foetal brain morphological development: a prospective study

Supplementary Methods

Appendix 1: The Methodology of Neurosonographical Measurements..4

Appendix 2: Details of Air Pollution Assessment 6

Appendix 3: The data on time-activity patterns..8

Appendix 4: Description of Multiple Imputation 11

Appendix 5: Directed Acrylic Graph...12

Tables and Figures

Table S5: Description of socioeconomic, demographic, and lifestyle characteristics of the recruited BiSC participants (N = 1080) and those with valid data included in this study (N = 754). 14

Figure S1: Spearman's correlation coefficients among NO_2, $PM_{2\cdot5}$, and BC assessed by different models. 15

Figure S2: Association of per IQR increases exposure to NO_2, $PM_{2\cdot5}$, and BC with the insula depth during each week of pregnancy (weeks 1 to 30). [a] (A: NO_2, B: $PM_{2\cdot5}$, C: BC) 16

Figure S3: Association of per IQR increases exposure to NO_2, $PM_{2\cdot5}$, and BC with the Sylvian fissure depth during each week of pregnancy (weeks 1 to 30). [a] (A: NO_2, B: $PM_{2\cdot5}$, C: BC) 19

Figure S4: Association of per IQR increases exposure to NO_2, $PM_{2\cdot5}$, and BC with the Parieto-occipital sulcus depth during each week of pregnancy (weeks 1 to 30). [a] (A: NO_2, B: $PM_{2\cdot5}$, C: BC) 22

Figure S5: Association of per IQR increases exposure to NO_2, $PM_{2\cdot5}$, and BC with the Cingulate sulcus depth during each week of pregnancy (weeks 1 to 30). [a] (A: NO_2, B: $PM_{2\cdot5}$, C: BC) 25

Figure S6: Association of per IQR increases exposure to NO_2, $PM_{2\cdot5}$, and BC with the Calcarine sulcus depth during each week of pregnancy (weeks 1 to 30). [a] (A: NO_2, B: $PM_{2\cdot5}$, C: BC) 28

Figure S7: Association of per IQR increases exposure to NO_2, $PM_{2\cdot5}$, and BC with the anterior lateral ventricle width during each week of pregnancy (weeks 1 to 30). [a] (A: NO_2, B: $PM_{2\cdot5}$, C: BC) 31

Figure S8: Association of per IQR increases exposure to NO_2, $PM_{2\cdot5}$, and BC with the posterior lateral ventricle width during each week of pregnancy (weeks 1 to 30). [a] (A: NO_2, B: $PM_{2\cdot5}$, C: BC) 34

Figure S9: Association of per IQR increases exposure to NO_2, $PM_{2\cdot5}$, and BC with the third ventricle width during each week of pregnancy (weeks 1 to 30). [a] (A: NO_2, B: $PM_{2\cdot5}$, C: BC) 37

Figure S10: Association of per IQR increases exposure to NO_2, $PM_{2\cdot5}$, and BC with the cisterna magna width during each week of pregnancy (weeks 1 to 30). [a] (A: NO_2, B: $PM_{2\cdot5}$, C: BC) 40

Figure S11: Association of per IQR increases exposure to NO_2, $PM_{2\cdot5}$, and BC with the corpus callosum length during each week of pregnancy (weeks 1 to 30). [a] (A: NO_2, B: $PM_{2\cdot5}$, C: BC) 43

Figure S12: Association of per IQR increases exposure to NO_2, $PM_{2\cdot5}$, and BC with the cerebellar vermis height during each week of pregnancy (weeks 1 to 30). [a] (A: NO_2, B: $PM_{2\cdot5}$, C: BC) 46

Figure S14: Association of per IQR increases exposure to NO_2, $PM_{2\cdot5}$, and BC with the percent difference in brain morphological structures (%) using all complete cases. [a]..52

Table S6: Association of per IQR increases exposure to NO_2, $PM_{2\cdot5}$, and BC with the percent difference in brain morphological structures (%) using all complete cases. [a]...53

Figure S15: Association of per IQR increases exposure to NO_2, $PM_{2\cdot5}$, and BC with the percent difference in brain morphological structures (%) further adjusting possible covariates. [a]..........................54

Table S7: Association of per IQR increases exposure to NO_2, $PM_{2·5}$, and BC with the percent difference in brain morphological structures (%) further adjusting possible covariates. [a] 55

Figure S16: Association of per IQR increases exposure to NO_2, $PM_{2·5}$, and BC with the percent difference in brain morphological structures (%) further adjusting prenatal complications. [a] (A: intrauterine growth retardation (IUGR); B: preeclampsia; C: gestational diabetes; D: gestational hypertension; E: all prenatal complications) 56

Table S8: Association of per IQR increases exposure to NO_2, $PM_{2·5}$, and BC with the percent difference in brain morphological structures (%) further adjusting prenatal complications. [a] (A: intrauterine growth retardation (IUGR); B: preeclampsia; C: gestational diabetes; D: gestational hypertension; E: all prenatal complications) 61

Figure S17: Association of per IQR increases exposure to NO_2, $PM_{2·5}$, and BC with the percent difference in brain morphological structures (%) after removing the participants diagnosed with one of the prenatal complications (i.e., intrauterine growth retardation (IUGR), preeclampsia, gestational diabetes, and gestational hypertension).[a] 66

Table S9: Association of per IQR increases exposure to NO_2, $PM_{2·5}$, and BC with the percent difference in brain morphological structures (%) after removing the participants diagnosed with one of the prenatal complications (i.e., intrauterine growth retardation (IUGR), preeclampsia, gestational diabetes, and gestational hypertension) 67

Figure S18: Association of per IQR increases exposure to NO_2, $PM_{2·5}$, and BC with the percent difference in brain morphological structures (%) further adjusted for the ambient temperature.[a] 68

Table S10: Association of per IQR increases exposure to NO_2, $PM_{2·5}$, and BC with the percent difference in brain morphological structures (%) further adjusted for the ambient temperature.[a] 69

Figure S19: Association of per IQR increases exposure to NO_2, $PM_{2·5}$, and BC with the percent difference in brain morphological structures (%) corrected by biparietal diameter. [a] 70

Table S11: Association of per IQR increases exposure to NO_2, $PM_{2·5}$, and BC with the percent difference in brain morphological structures (%) corrected by biparietal diameter. [a] 71

Figure S20: Association of per IQR increases exposure to NO2, PM2·5, and BC in each microenvironment with the percent difference in brain morphological structures (%). a (A: NO2; B: PM2·5; C: BC) 72

Table S12: Association of per IQR increases exposure to NO_2, $PM_{2·5}$, and BC in each microenvironment with the percent difference in brain morphological structures (%). [a] (A: NO_2; B: $PM_{2·5}$; C: BC) 75

Figure S21: Association of per IQR increases exposure to NO_2, $PM_{2·5}$, and BC with the percent difference in brain morphological structures (%).[a] (A: Land use regression model, B: Dispersion models) 78

Table S13: Association of per IQR increases exposure to NO_2, $PM_{2·5}$, and BC with the percent difference in brain morphological structures (%).[a] (A: Land use regression model, B: Dispersion models) 80

Figure S22: Association of per IQR increases exposure to NO_2, $PM_{2·5}$, and BC with the percent difference in anterior and posterior horn lateral ventricles for both left and right (%). [a] (A: Anterior horn lateral ventricles; B: Posterior horn lateral ventricles) 82

Table S14: Association of per IQR increases exposure to NO_2, $PM_{2·5}$, and BC with the percent difference in anterior and posterior horn lateral ventricles for both left and right (%). [a] 83

Figure S23: Association of per IQR increases exposure to NO_2, $PM_{2·5}$, and BC with the percent difference in brain morphological structures after removing the participants with active and passive smoking exposure during pregnancy (%).[a] (A: Removing Active Smoking Exposure Participants; B: Removing Passive Smoking Exposure Participants) 84

Table S15: Association of per IQR increases exposure to NO_2, $PM_{2·5}$, and BC with the percent difference in brain morphological structures after removing the participants with active and passive smoking exposure during pregnancy (%).[a] (A: Removing Active Smoking Exposure Participants; B: Removing Passive Smoking Exposure Participants) 86

Figure S24: Association of per IQR increases exposure to NO_2, $PM_{2·5}$, and BC with the percent difference in brain morphological structures adjusting the p-value for multiple comparisons (%).[a] (A: NO_2; B: $PM_{2·5}$; C: BC) 88

Figure S25: Association of per IQR increases exposure to NO_2, $PM_{2·5}$, and BC with the percent difference in brain morphological structures (%) stratified by foetal sex. [a] (A: NO_2; B: $PM_{2·5}$; C: BC) 91

Table S16: Association of per IQR increases exposure to NO_2, $PM_{2·5}$, and BC with the percent difference in brain morphological structures (%) stratified by foetal sex. [a] (A: NO_2; B: $PM_{2·5}$; C: BC) 94

Supplementary Methods:

Appendix 1: The Methodology of Neurosonographical Measurements

Equipment used: GE Voluson 10 Expert scanner (GE Healthcare, Illinous, USA) equipped with a 4-8 MHz linear array (RAB4-8-D probe and 4C-D probe) was applied in Hospital Clínic – La Maternitat and Hospital Sant Joan de Déu, and EPIQ 7W and Affiniti 70G (Philips Healthcare, Andover) ultrasound systems equipped with a probe at 9-MHz (C9-2, Philips Medical Systems, Andover, MA, USA) was used in Hospital de Sant Pau i la Santa Creu, Hospital Clínic. Obstetricians previously attended an intensive course to learn fetal neurosonography from experts and participated in clinical rotations in neurosonography units of each hospital.

Measurement: Depth of brain sulci and fissures was measured in millimetres and following different anatomical references as described in previous studies.[1,2] The insula depth was measured in the transthalamic axial plane (as a perpendicular line from the midline behind the cave of the septum pellucidum to the cortex external border). Sylvian fissure was measured in the same plane as the insula (extending the line of the insula depth, starting at the cortex external border and finishing at the cranium internal border). Parieto-occipital sulcus depth wasin a plane superior to the transventricular plane (base starting from the midline as symmetric as possible but excluding the cortex). Cingulate sulcus depth was assessed in a coronal view of the transthalamic plane (following a perpendicular line from the midline to the apex of the sulcus excluding the cortex). Calcarine sulcus depth was measured in a coronal view of the transcerebellar plane (as a perpendicular line from midline to the sulcus apex excluding the cortex). In addition, the transventricular plane (visualizing the anterior and posterior horns of the lateral ventricles), was used to measure both anterior and posterior horns of the lateral ventricles. The axial transthalamic plane (showing the cave of septum pellucidum, thalamus, and hippocampal gyruses) was used to measure basic structures, such as biparietal diameter (BPD), as the largest symmetrical axial view of the foetal head, head circumference (HC), occipitofrontal diameter (OFD) biparietal diameter (BPD) and third ventricle. The transcerebellar plane (slightly lower than the transventricular plane with a posterior tilting that allows the visualization of the following structures) was used to measure the cisterna magna and the cerebellum transversal diameter. Corpus callosum and cerebellar vermis length were measured by the midsagittal plane.

Processing: In a first step, missing data for each of the neurosonography measures were detected and retrieved to obstetricians, who helped to recover these data from BiSC ultrasound images. Clinical plausibility was used as the criteria for inconsistent data and outlier detection besides classical statistical definition (based on the values' deviation from the mean). For this purpose, the INTERGROWTH-21st foetal growth standards were used as reference values for basic foetal growth sonography structures,[3] and the ISUOG normal value ranges were used for specific neurosonographical structures (eg. lateral ventricle's posterior horns should not be over 10 mm).[4] These outliers were selected and sent to obstetricians for double check: data entries were reviewed (and corrected, if needed) and remaining outliers were classified into physiologically possible values (clinically relevant values, which were confirmed and kept) or impossible values (which were set to missing). The consequent changes were included in the centralized database. This

process was repeated with a second round of checks by the data manager and any remaining changes were included before considering the datasets final.

Reproducibility: To assess the reliability of clinical measurements, we determined the interobserver variability of the neurosonographical structures. For this purpose, 30 patients (15 of each hospital unit: either Hospital de Sant Pau i la Santa Creu or Hospital Sant Joan de Déu and Hospital Clínic - La Maternitat) were randomly selected to be evaluated by two blinded raters (M.J.Z. and M.M). Images of examinations were not specifically selected for reevaluation of each variable. Following Priya Ranganathan's guideline for measures of agreement, we used the intra-class correlation coefficient (ICC) for the outcome variables.[5] Based on the characteristics of our study follow-up (we assessed the two raters of interest who performed all the measurements of the cohort) and following Therry K. Koo's Guideline for selecting and reporting ICC for reliability research,[6] ICC for interobserver variability ICC was calculated using 2-way random effect model with absolute agreement. (Table S3).

Table S1: Measures of agreement for a set of selected neurosonographical structures.

	**Variables**	**Inter-rater ICC**
	Insula depth	0·67
	Sylvian fissure depth	0·78
**Cortical folding**	Parieto-occipital fissure depth	0·45
	Cingulate fissure depth	0·64
	Calcarine fissure depth	0·65
	Anterior lateral ventricles (left)	0·68
	Anterior lateral ventricles (right)	0·77
**CSF spaces**	Posterior lateral ventricles (left)	Unavailable data
	Posterior lateral ventricles (right)	Unavailable data
	Third ventricle width	0·80
	Cisterna magna	0·75
	Corpus callosum length	0·85
**Others**	Cerebellar vermis length	0·86
	Transcerebellar diameter	0·78
	Average ICC	0·72

Abbreviation: CSF, Cerebrospinal fluid; ICC, intra-class correlation coefficient; HSJD, Hospital Sant Joan de Déu and Hospital Clínic - La Maternitat; HSP, Hospital de Sant Pau i la Santa Creu.

Appendix 2: Details of Air Pollution Assessment

BiSCAPE air pollution monitoring campaigns: we conducted monitoring campaigns of ambient $PM_{2\cdot5}$ (37mm Teflon filters collected using a BGI-400 pumps working at 4 L/min and a PCIS impactor), BC (MicroAeth® AE51), and NO_2 (Gradko NO_2 diffusion tubes) for developing land use regression (LUR) models to estimate air pollutants exposure of participants. To cover intra-annual variability in the pollution surface, we carried out three monitoring campaigns in different seasons from January 2021 to February 2022 (first campaign: 28th June 2021 - 28th July 2021; second campaign: 11th October 2021 - 12th November 2021; third campaign: 12th January 2022 - 16th February 2022). We also had an extra campaign from 16th February 2021 to 29th March 2021 where we collected data on NO_2 and BC. We monitored air pollutant levels in these sites for an average period of nine days in each campaign. Sites were located approximately at a first-floor height. The following criteria were also fulfilled when placing the samplers: (i) not to be placed near exhaust flues, chimneys, air conditioning devices or tee-lines, (ii) location should be smoking-free area, (iii) they should be placed at approximate 0·5 to 1·5 m above the floor. Trained fieldworkers logged the GPS coordinates (exact location) and height to street ground floor. Moreover, the fieldworkers collected data on installation and collection times of the different samplers, and registered (and corrected if needed) $PM_{2\cdot5}$ pump flow at the beginning, middle and end of each data collection period in each location. In addition, any incidences that may have affected the measurements were also recorded (e.g., the power supply was turned off during the data collection). Following the European Study of Cohorts for Air Pollution Effects (ESCAPE) protocols,[7,8] we selected 34 representative sites including urban traffic and background sites and one reference urban background station, that represented the gradient of various land use, emission sources, and traffic characteristics across the BiSC study area.

Table S2: Description for the LUR models for NO_2, black carbon (BC), and $PM_{2\cdot5}$ in terms of the years of data collection (Year), number of data points used to develop the model (N), the adjusted coefficient of determination (Adj-R^2), cross-validation R^2 (CV-R^2), residual standard error (RSE), and predictor variables that remained in the final model.

Pollutant	Year	N	Adj-R^2	CV-R^2	RSE	Predictor variables [a]
**NO_2**	2018-2020	483	0·62	0·62	4·03	trafload25 - sqralt + majorroadlength50 + roadlength25 + majorroadlength300
**$PM_{2\cdot5}$**	2021	34	0·47	0·45	1·47	hdres500 + trafnear + LEZ
**BC**	2021	30	0·85	0·83	0·18	hdres50 + linesnear + pop300 + trafload500 + roads500

[a]Predictor variables: trafload, total traffic intensity (veh/day); sqralt, squared root altitude (m1/2); majorroadlength, total major road length (m); roadlength, total road length (m); hdres, high-density residential area (m^2); trafnear, traffic intensity at the nearest road (veh/day); LEZ, Low Emissions Zone (Yes/No, ref value=No); linesnear, number traffic lines on nearest street; roads, roads surface area (m^2); ind, industry area (m^2); pop, population density (inhabitants).

Table S3: External validation of NO_2 level estimates by the dispersion model.

Validation dataset	Number of validation observationsa	Adjustment	External validation R^2
BiSCAPE sampling sites	98	Unadjusted	**0·65**
Participants' home-outdoor measures	1554	Unadjusted	**0·44**
Participants' personal measures	1660	Unadjusted	**0·10**
	1515	Adjusted for the indoor/outdoor ratio[a]	**0·32**

[a] The number of data point-weeks.

[b] For all times that the participant was at home, we multiplied the ambient NO_2 level predicted by the dispersion model by the home-indoor/home-outdoor ratio for that participant (The NO_2 levels at home-indoor and home-outdoor were collected during the same campaign of collecting personal NO_2 and time-activity data).

Table S4: Description for the BiSC Hybrid models for NO_2, $PM_{2·5}$, and BC in terms of the number of data points used to develop the model (N), 10-fold-cross-validation R^2 (10-CV R^2) and root mean square error (10-CV RMSE) and predictor variables.

Pollutant	Year	N	Performance 10-CV R^2	Metrics 10-CV RMSE	Predictor variables [a]
**NO_2**	2018-2021	1232	0·64	7·5	NO2_Dispersion + idw_no2_monitoring_station + distinvmajor1 + majorroadlength100 +majorroadlength50 + trafload25 + trafnear + avg_traffic_stations + avg_solar_radiation + sqralt + ldres1000 +ldres500 + hdres1000 + build1000 + roads100 + lat + lon
**$PM_{2·5}$**	2021	161	0·66	3·45	PM25_Dispersion + idw_pm25monitoring_stations + avg_traffic_stations + ldres1000 + build25 + pop1000 + roads25 + sqralt + majorroadlength25 + trafload300 + build_height_25 + lat + lon
**BC**	2021	74	0·86	0·23	BC_Dispersion + idw_nox_monitoring_stations + avg_atmospheric_pressure + avg_bc_palau_reial + pop100 + hdres300 + roads25 + avg_wind_speed + roadlength25 + hdres50 + distinvmajor1 + avg_traffic_stations + linesnear + pop300 + trafload500 + roads500

[a]Predictor variables: NO_2_Dispersion, dispersion estimates (μg/m^3); PM25_Dispersion, dispersion estimates $PM_{2.5}$ (μg/m^3); BC_Dispersion, dispersion estimates BC (μg/m^3); idw_no2_monitoring_station, weekly NO_2 inverse distance weighting interpolation estimates from XVCPA (μg/m^3); idw_pm25_monitoring_station, weekly $PM_{2.5}$ inverse distance weighting interpolation estimates from XVCPA (μg/m^3); idw_nox_monitoring_station, weekly NOx inverse distance weighting interpolation estimates from XVCPA (μg/m^3); avg_bc_palau_reial, weekly BC average concentration from Palau Reial monitoring station (ug/m^3); avg_traffic_stations, weekly average count of vehicles in AMB (count); avg_solar_radiation, weekly average solar radiation from Raval station (MJ/m^2); avg_wind_speed, weekly average wind speed from Raval station (km/h); avg_atmospheric_pressure, atmospheric pressure from Raval station (hPA); majorroadlength, total major road length (m); trafload, total traffic intensity (veh/day); trafnear, traffic intensity at the nearest road (veh/day); linesnear, number traffic lines on nearest street; hdres, high-density residential area (m^2); roads, roads surface area (m^2); pop, population density (inhabitants); build, building area (m^2); ldress, low density residential area (m^2); trafmajor, total major roads traffic intensity (veh/day); build_height, averaged building height (meters); lat, latitude (m); sqralt, squared root altitude (m1/2).

Appendix 3: The data on time-activity patterns

First, for this work, we estimated the air pollution exposure at 3 microenvironments: 1) home, 2) commuting (both outward and inward), 3) work or main place of occupation.

Second, we obtained information from the participants regarding their time-activity patterns and locations via different sources of information:

1) Questionnaires and/or interviews by fieldworkers (at 12 weeks and 32 weeks gestation). Questions: how long spent at home, workplace, and commuting; and Questions: commuting mode of transportation.
2) Manual coding of the typical commuting routes in a Geographic Information System (GIS) platform (i.e., QGIS) during the home visits by our trained fieldworkers at 12 weeks and 32 weeks gestation. Besides spatially drawing the specific route the participants most commonly took to and from work (or main occupation) we registered information on commuting times and modes of transport. This data was only collected before the COVID lockdown measures as we were not able to do such a detailed assessment due to health concerns.
3) Global Position System (GPS) monitoring during the personal measurements (total 2 weeks, one week respectively around 12 weeks and 32 weeks gestation) using the ExpoApp.

Third, we followed the following approach:

1) If data was available from different sources: Data collected by the fieldworkers during the visits using QGIS was prioritized over the data collected via GPS. Because it was meant to show the long-term routine route while the GPS data was conducted during only two weeks of pregnancy and the time-activity pattern during those two weeks might have varied from the long-term routine.
2) If the data on QGIS was not available, we applied the GPS data (collectd by ExpoApp): we identified clusters to be assigned to home (for which we already had the information) and workplace (or main place of occupation). We identified the journeys between home and workplace and selected the most ‘typical’ ones (one inward and one outward) in terms of duration, and distance time of the day, with the best GPS accuracy.
   The next figure was an example of data collected by ExpoApp. Each ExpoApp file collected 7 days, 24h of GPS data (points with timestamps) for one participant. This information can 1) identify clusters corresponding to home and workplace; 2) identify journeys between home and workplace; 3) Select the most “typicals” (one inward and one outward) in terms of duration, distance time of the day, with the best GPS accuracy

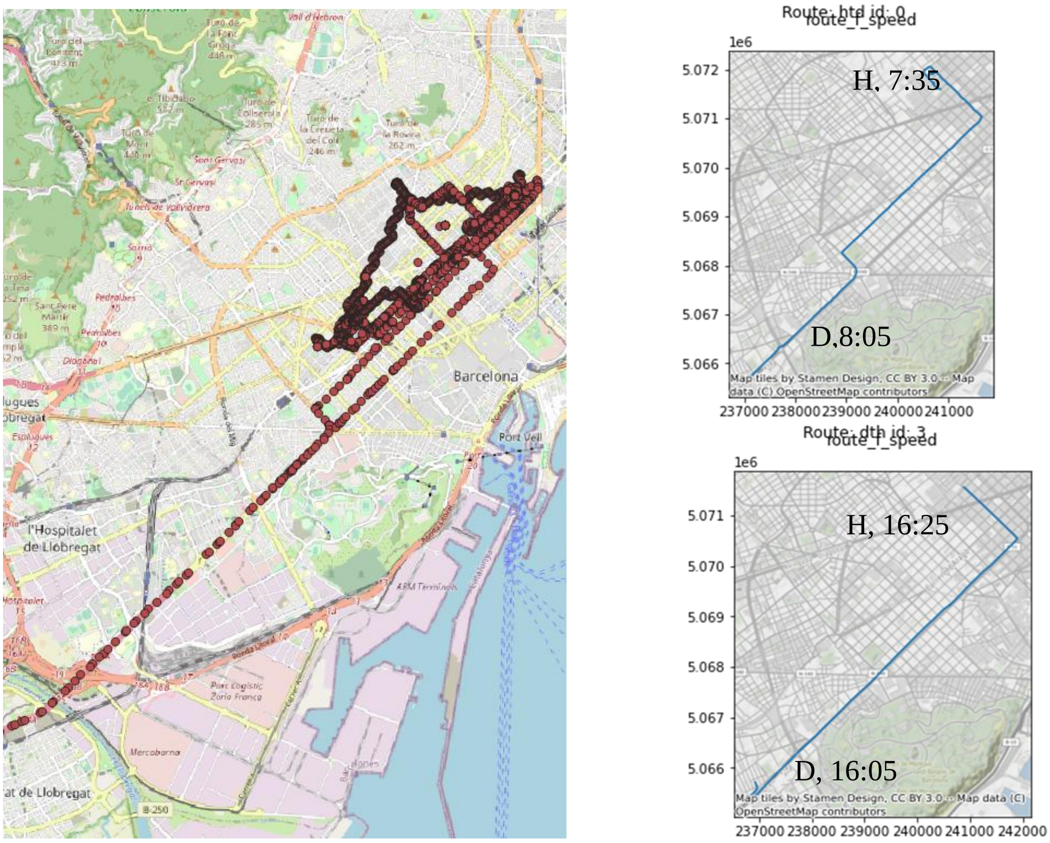

3) If only home and workplace addresses were available (through questionnaires): we used a routing service to estimate commuting routes, incorporating the mode of transport used (when available). Commuting time was estimated from the Spanish Statistical Office (INE) based on the 2010 study of time use in Spain, which could better reflect typical commuting durations for each transport mode. The next figure shows about the routing service:

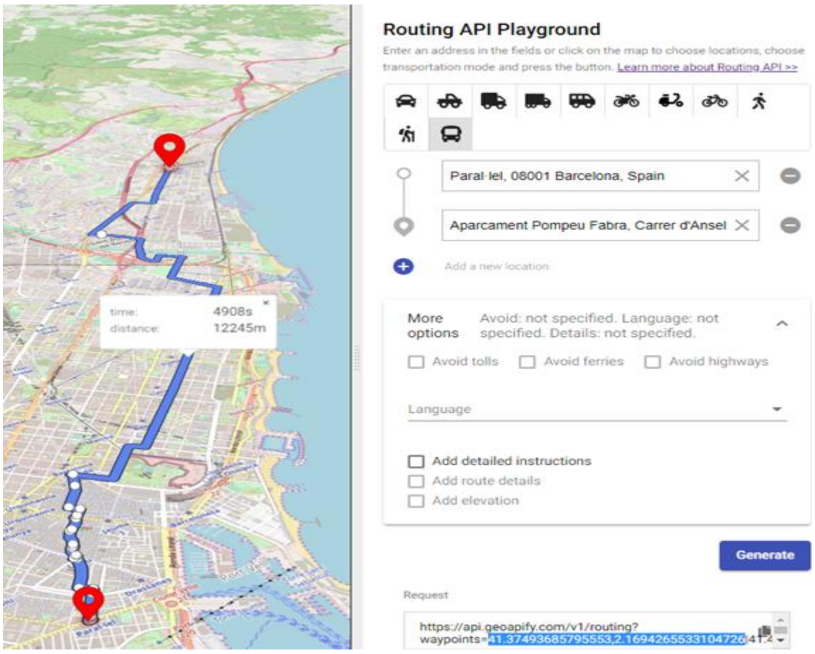

The information about INE 2010 you can find in here: https://www.ine.es/dynt3/inebase/index.htm?type=pcaxis&path=/t25/e447/a2009-2010/p01/&file=pcaxis&L=1

The final data available used is the following (H = Home, D = Destination, which means work or main place of occupation):

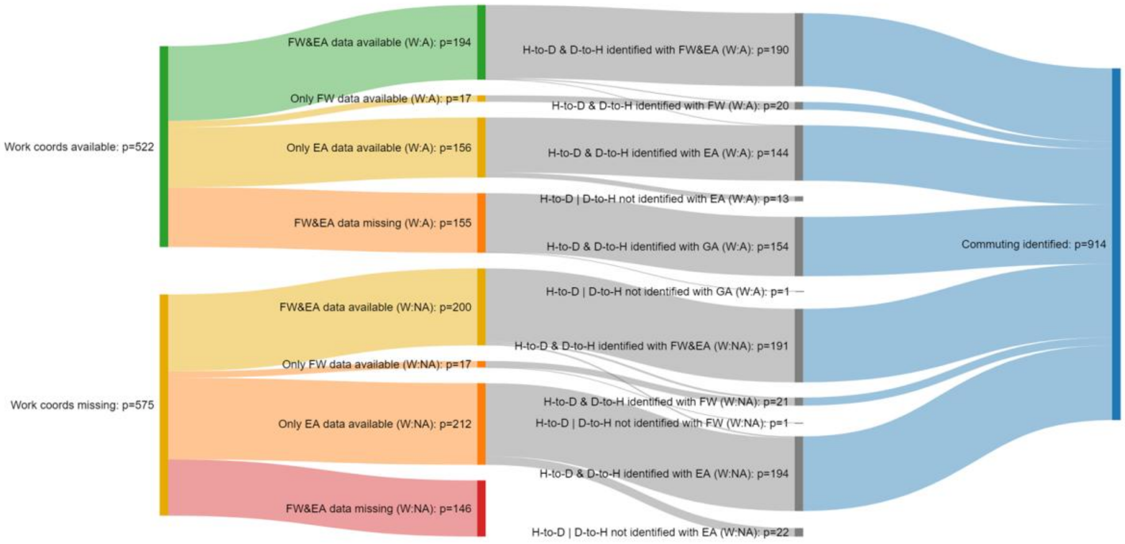

Finally, this data was used to determine whether the participant was at home, work or commuting and at which periods of the day. Using the geolocation established in the previous time-activity pattern, we obtained the exposures to air pollution using the different models at the three environments.

We have added these explanations to our supplementary appendices (Appendix 3: The data on time-activity patterns).

Appendix 4: Description of Multiple Imputation

Software used and version: R (4.2.2)

Function: mice function from mice package (Version 3.15.0)

Number of imputed datasets created: 100

Variables included in the imputation procedure: Variables used in the main analyses as covariates together with other relevant covariates: alcohol consumption during pregnancy, gestational age at birth, maternal pre-pregnancy thyroid pathology, estimated fetal weight at 32w visit, maternal body mass index, weight at birth, any breastfeeding at first month, maternal age, season of conception, year of conception and employ status.

Method for numeric data: Predictive mean matching

Method for binary data, factor with 2 levels: Logistic regression

The percentage of imputed values for each covariable: 3·6% for active smoking during pregnancy, 4·0% for passive smoking during pregnancy, 4·6% for alcohol consumption during pregnancy, 0·7% for maternal pre-pregnancy thyroid pathology, 6·9% for IUGR, preeclampsia, gestational diabetes, and gestational hypertension, and 5·7% for maternal body mass index·

Appendix 5: Directed Acrylic Graph

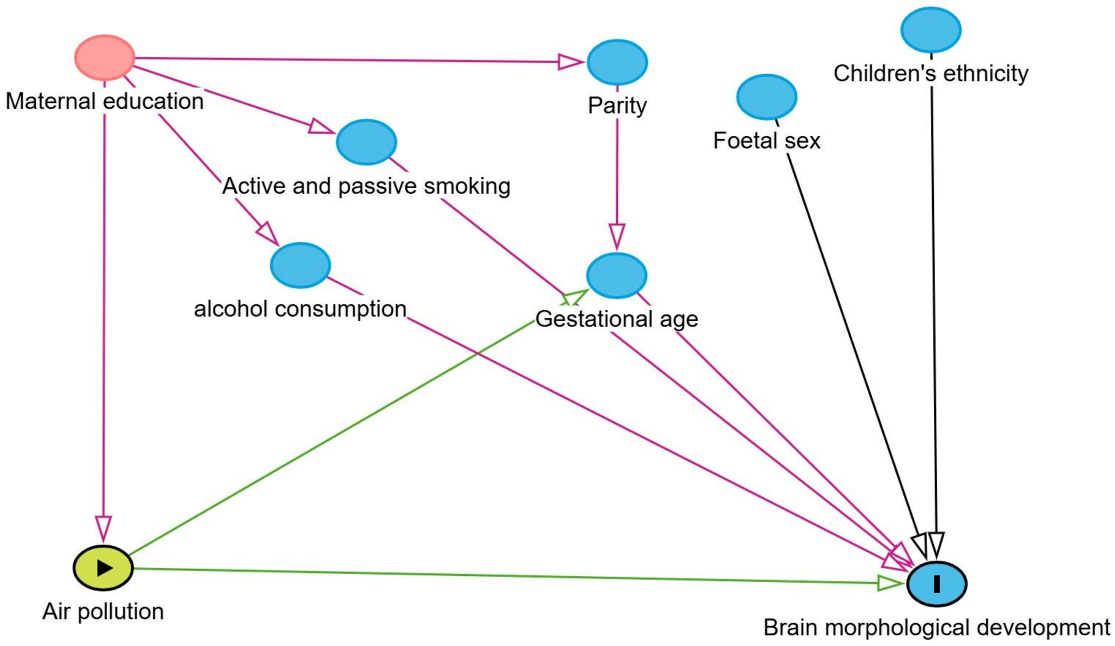

Directed acyclic graph for the association between prenatal air pollution exposure and foetal brain morphological development by dagitty.net (www.dagitty.net).

Reference

1 Basso A, Youssef L, Nakaki A, *et al.* Fetal neurosonography at 31–35 weeks reveals altered cortical development in pre-eclampsia with and without small-for-gestational-age fetus. *Ultrasound Obstet Gynecol* 2022; **59**: 737–46.

2 Alonso I, Borenstein M, Grant G, Narbona I, Azumendi G. Depth of brain fissures in normal fetuses by prenatal ultrasound between 19 and 30 weeks of gestation. *Ultrasound Obstet Gynecol* 2010; **36**: 693–9.

3 Papageorghiou AT, Kennedy SH, Salomon LJ, *et al.* The INTERGROWTH-21st fetal growth standards: toward the global integration of pregnancy and pediatric care. *Am J Obstet Gynecol* 2018; **218**: S630–40.

4 Malinger G, Paladini D, Haratz KK, Monteagudo A, Pilu GL, Timor-Tritsch IE. ISUOG Practice Guidelines (updated): sonographic examination of the fetal central nervous system. Part 1: performance of screening examination and indications for targeted neurosonography. *Ultrasound Obstet Gynecol* 2020; **56**: 476–84.

5 Ranganathan P, Pramesh C, Aggarwal R. Common pitfalls in statistical analysis: Measures of agreement. *Perspect Clin Res* 2017; **8**: 187–91.

6 Koo TK, Li MY. A Guideline of Selecting and Reporting Intraclass Correlation Coefficients for Reliability Research. *J Chiropr Med* 2016; **15**: 155–63.

7 Beelen R, Hoek G, Vienneau D, *et al.* Development of NO2 and NOx land use regression models for estimating air pollution exposure in 36 study areas in Europe - The ESCAPE project. *Atmos Environ* 2013; **72**. DOI:10.1016/j.atmosenv.2013.02.037.

8 Eeftens M, Beelen R, De Hoogh K, *et al.* Development of land use regression models for PM2.5, PM 2.5 absorbance, PM10 and PMcoarse in 20 European study areas; Results of the ESCAPE project. *Environ Sci Technol* 2012; **46**: 11195–205.

Appendix 6: Tables and Figures

Table S5: Description of socioeconomic, demographic, and lifestyle characteristics of the recruited BiSC participants (N = 1080) and those with valid data included in this study (N = 754).

**Participant's characteristics**	**BiSC (N=1080)**	**Our study (N= 754)**	***p*-value**
**Foetal sex**			
Girl	517 (47·9%)	369 (48·9%)	0·83
Boy	526 (48·7%)	385 (51·1%)	
NA	37 (3·4%)	0	
**Mother parity**			
Multiparous	474 (43·9%)	323 (42·8%)	0·69
Nulliparous	606 (56·1%)	432 (57·2%)	
**Maternal educational level**			
With university degree	747 (69·2%)	517 (68·6%)	0·86
Without university degree	333 (30·8%)	237 (31·4%)	
**Maternal ethnicity**			
European-Caucasian	725 (67·1%)	512 (67·9%)	0·77
Other ethnicities	355(32·9%)	242 (32·1%)	
**Active smoking during pregnancy**			
No	932 (86·3%)	668 (88·6%)	1·00
Yes	83 (7·7%)	59 (7·8%)	
NA	65 (6·02%)	27 (3·6%)	
**Passive smoking during pregnancy**			
No	576 (53·3%)	408 (54·1%)	0·84
Yes	435 (40·3%)	316 (41·8%)	
NA	69 (6·4%)	30 (4·0%)	
**Alcohol consumption during pregnancy**			
No	702 (65·0%)	507 (67·3%)	0·81
Yes	303 (28·1%)	212 (28·2%)	
NA	75 (6·9%)	35 (4·6%)	
**Gestational age at delivery (days)**	280 ± 12	280 ± 11	0·58

Figure S1: Spearman's correlation coefficients among NO_2, $PM_{2·5}$, and BC assessed by different models.

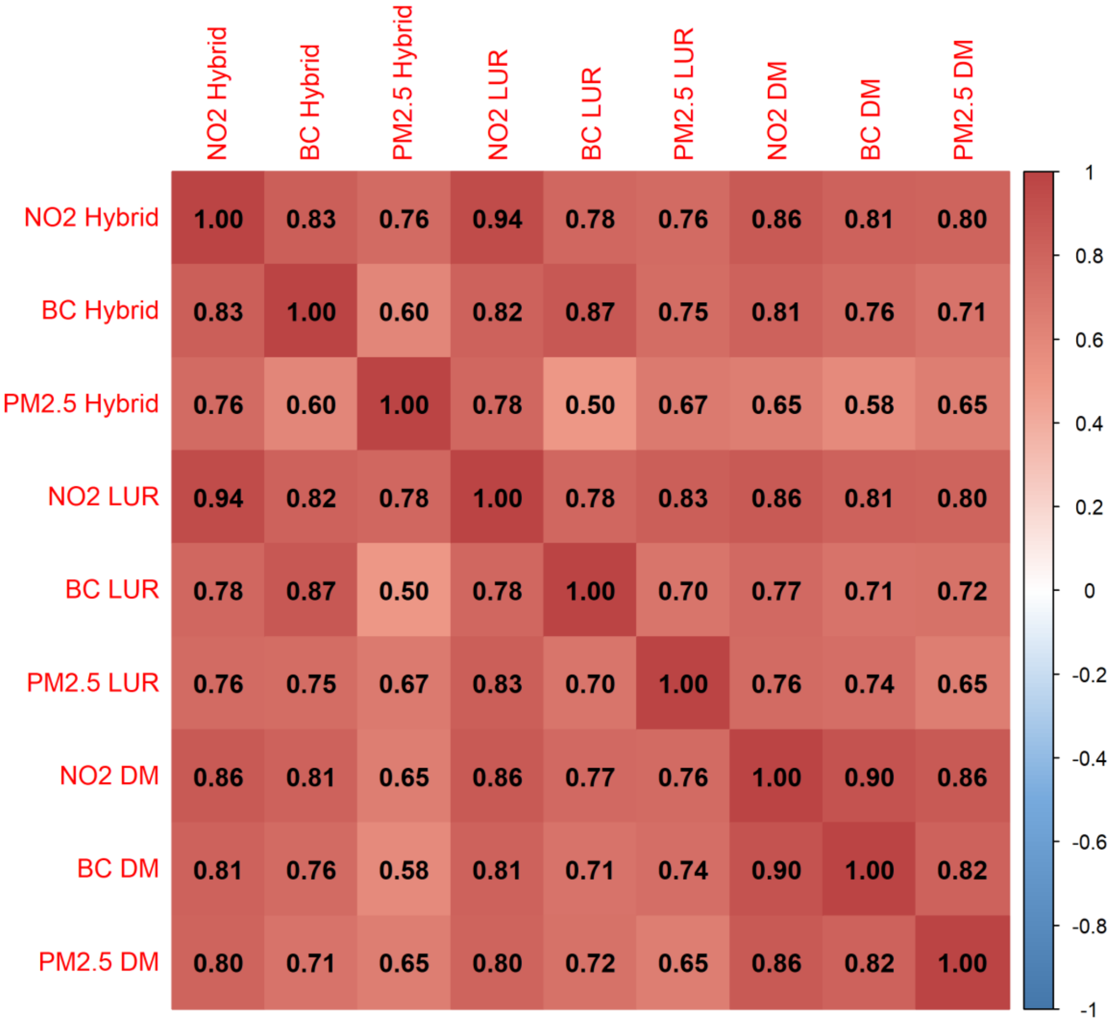

Abbreviation: LUR models, land use regression models; DMs, dispersion models; NO_2, nitrogen dioxide; $PM_{2·5}$, particulate matter with an aerodynamic diameter < 2·5 µm; BC, black carbon.

Figure S2: Association of per IQR increases exposure to NO_2, $PM_{2\cdot5}$, and BC with the insula depth during each week of pregnancy (weeks 1 to 30). [a] (A: NO_2, B: $PM_{2\cdot5}$, C: BC)

(A)

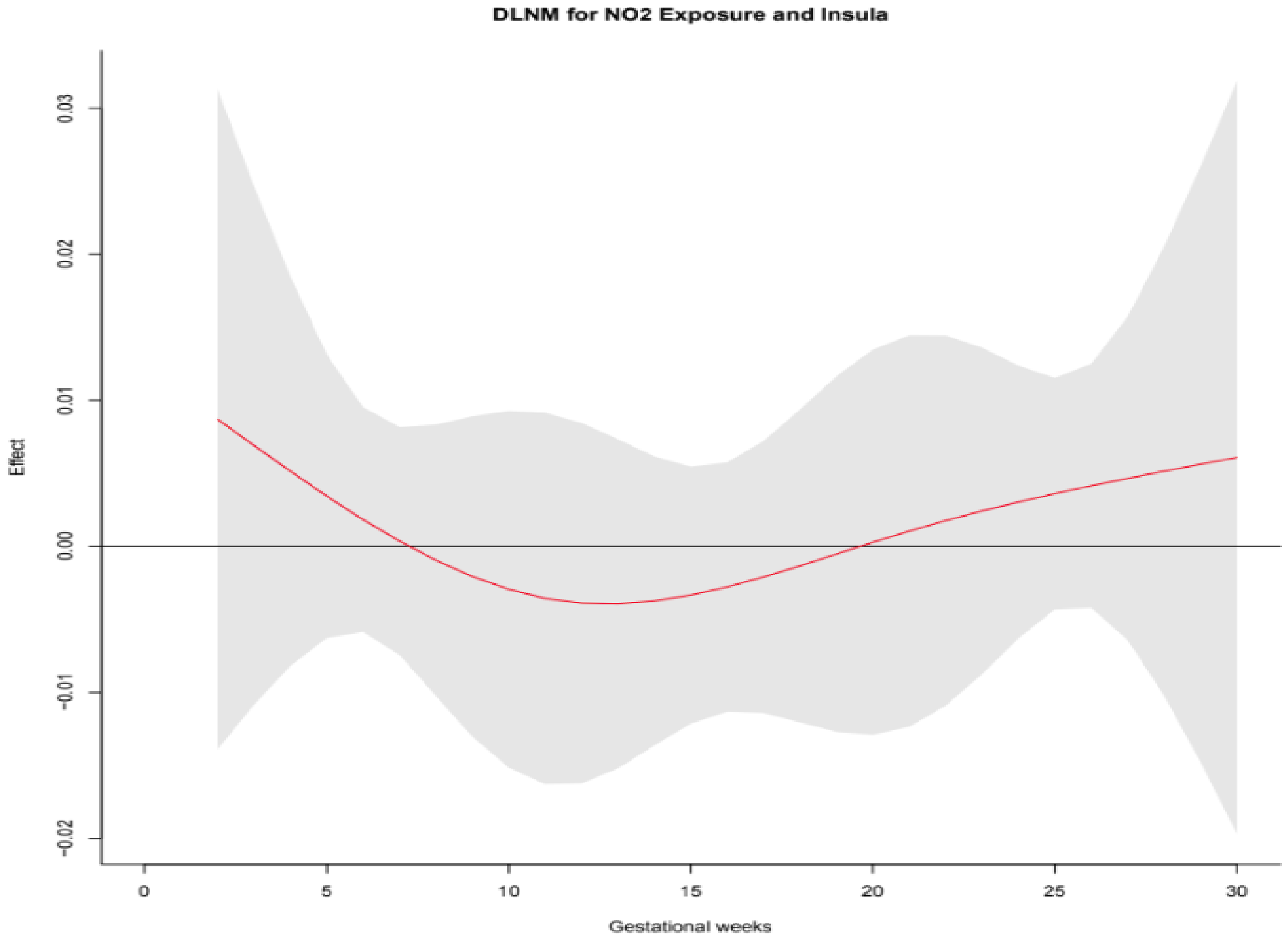

(B)

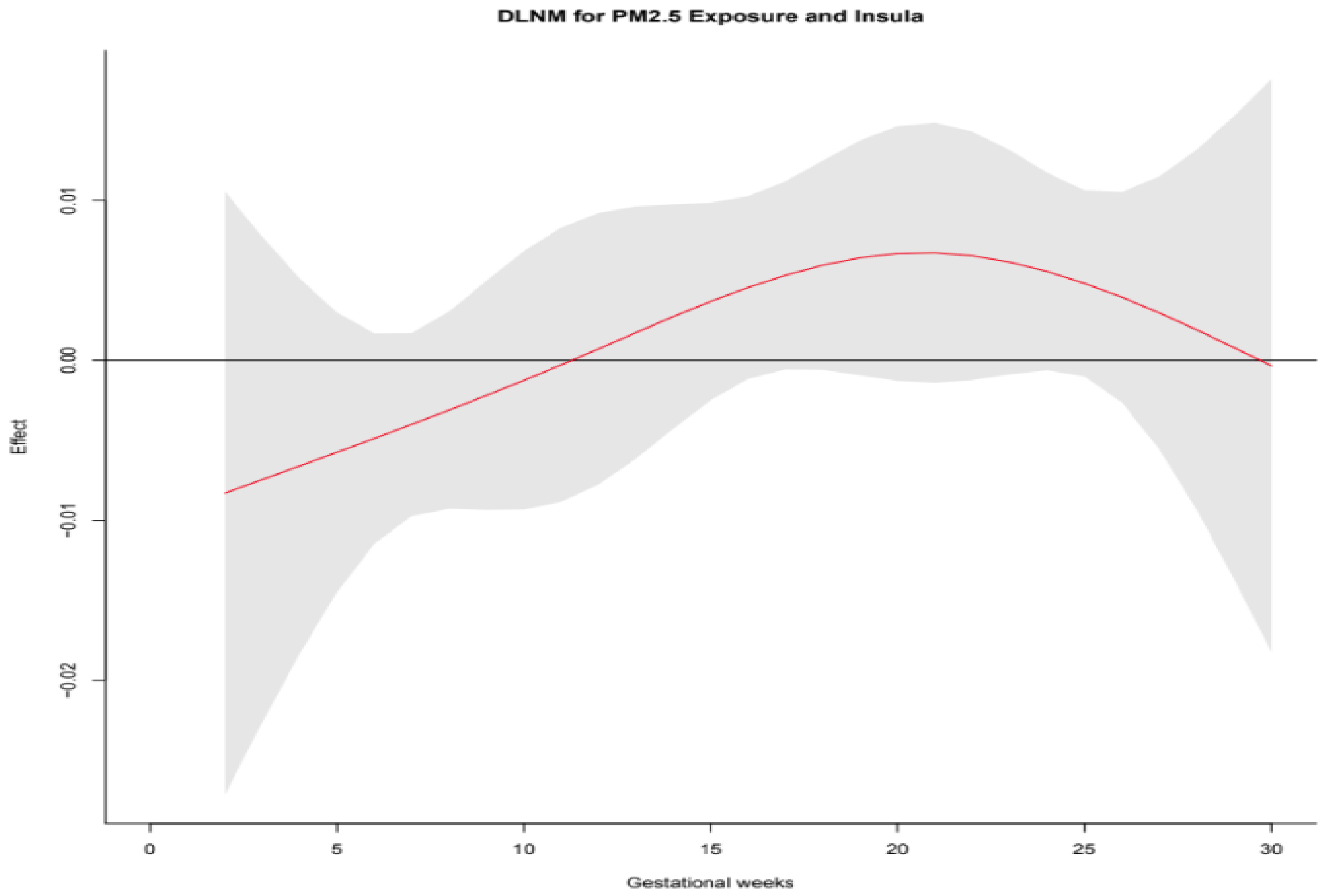
DLNM for PM2.5 Exposure and Insula
Effect
0.01
0.00
−0.01
−0.02
0
5
10
15
20
25
30
Gestational weeks

(C)

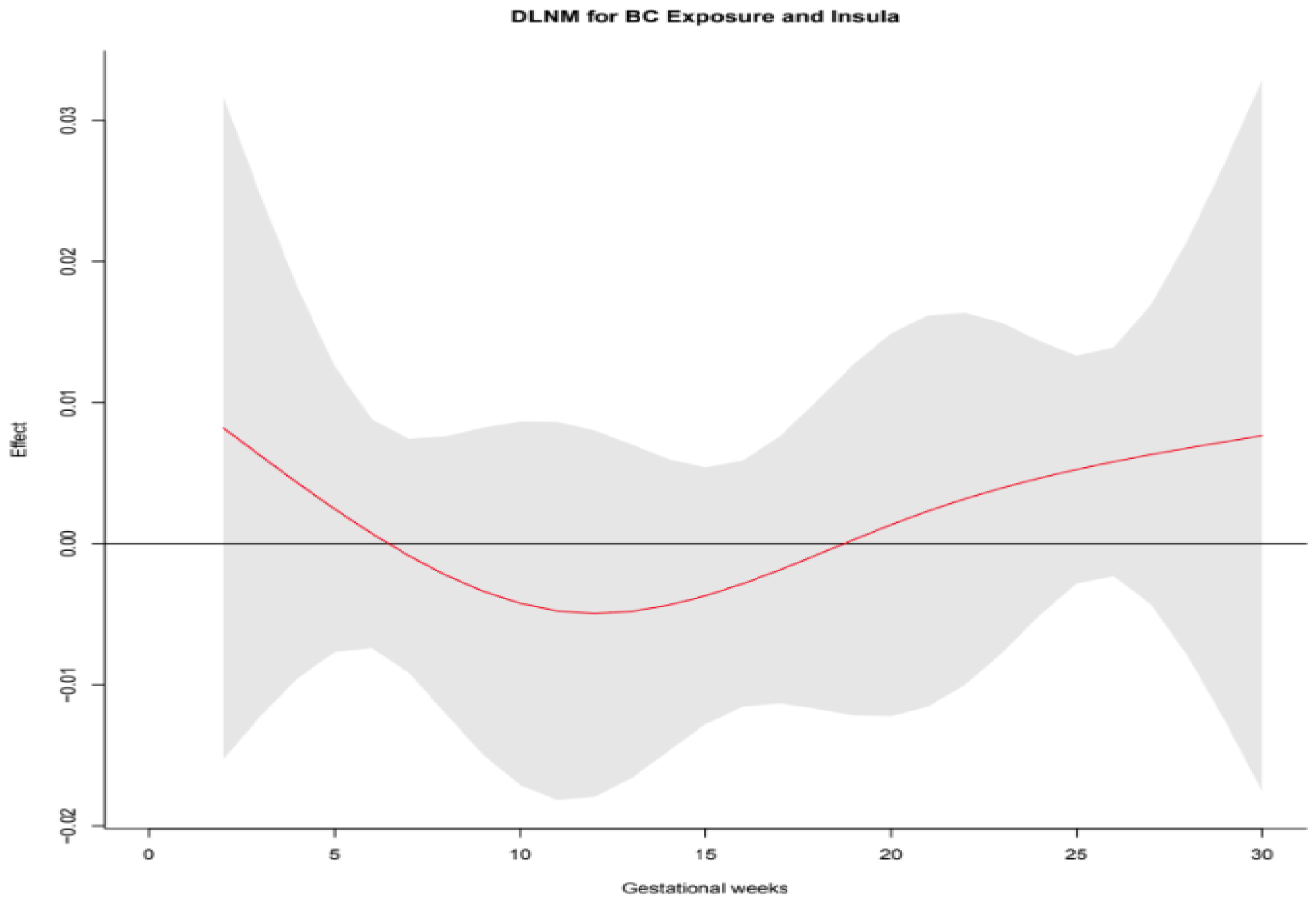

[a] Adjusted for foetal sex (boy vs girl), mother parity (multiparous vs nulliparous), maternal education (with university degree vs without university degree), ethnicity (European vs other), active smoking during pregnancy (no vs yes), passive smoking during pregnancy (no vs yes), alcohol consumption during pregnancy (no vs yes), and gestational age at ultrasound (days), and hospital and rater as random effects.

Abbreviations: NO_2, nitrogen dioxide; $PM_{2·5}$, particulate matter with an aerodynamic diameter < 2·5 µm; BC, black carbon; DLNM, Distributed Lag Non-linear Models.

Figure S3: Association of per IQR increases exposure to NO_2, $PM_{2\cdot5}$, and BC with the Sylvian fissure depth during each week of pregnancy (weeks 1 to 30). [a] (A: NO_2, B: $PM_{2\cdot5}$, C: BC)

(A)

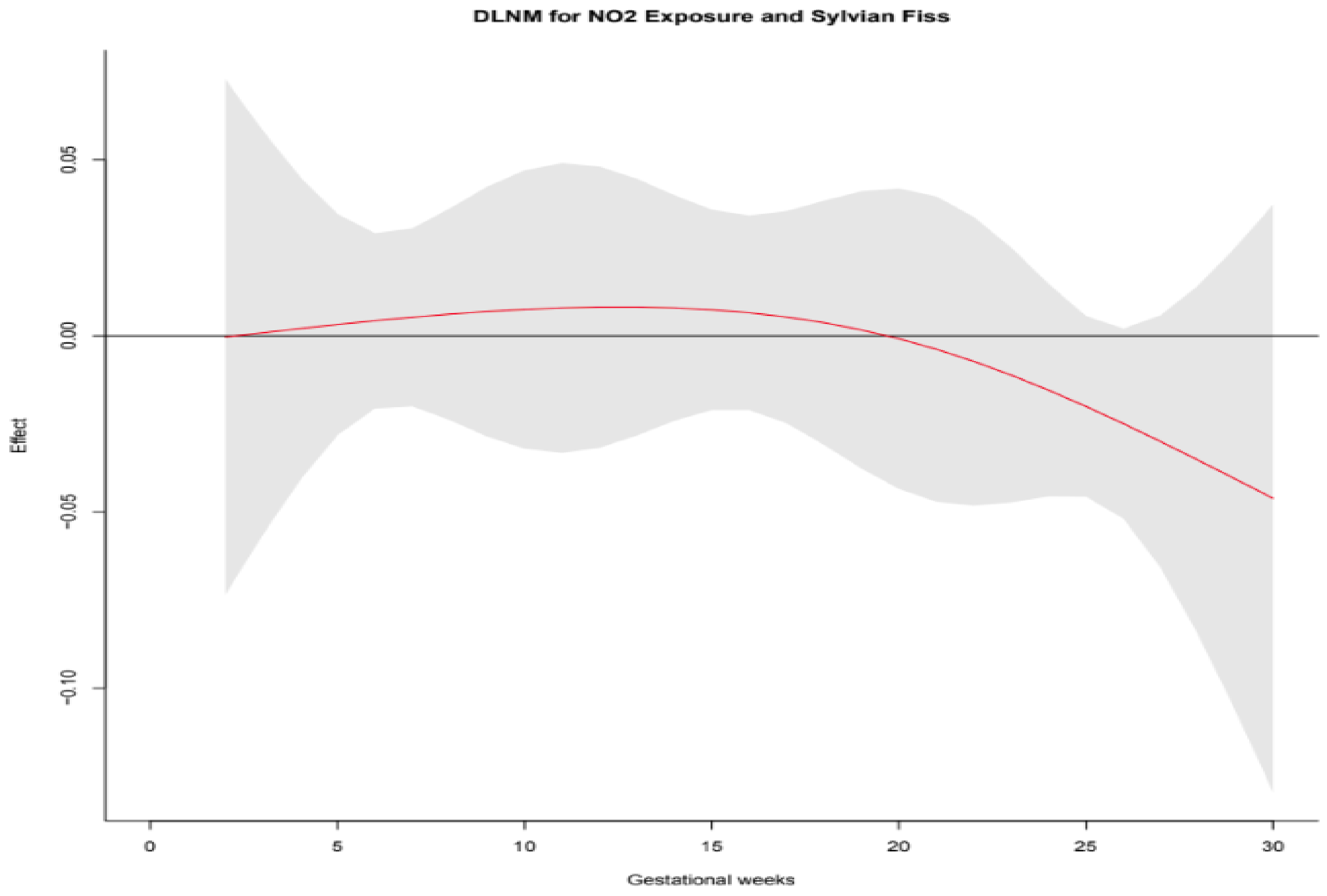

(B)

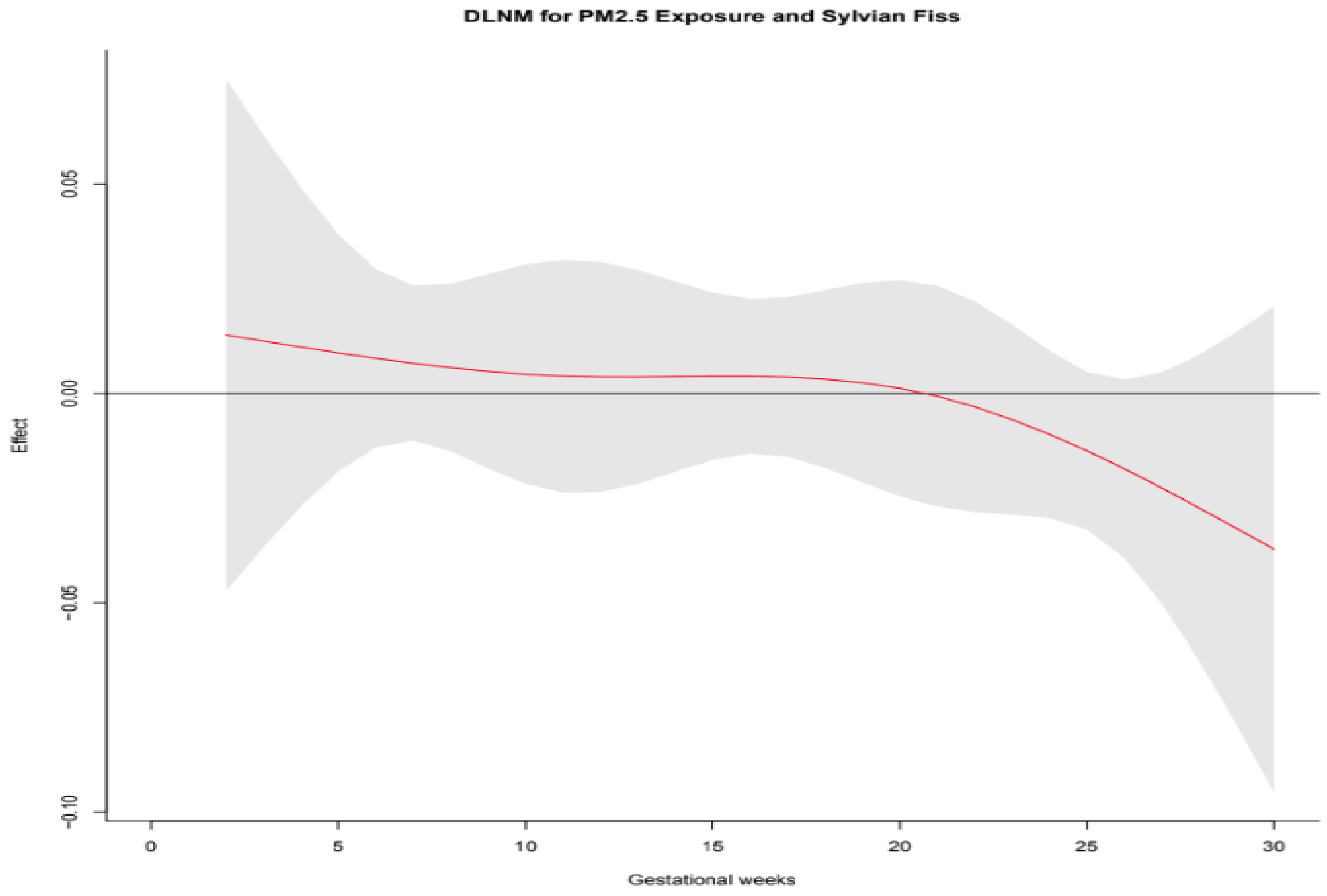
DLNM for PM2.5 Exposure and Sylvian Fiss
Effect
0.05
0.00
−0.05
−0.10
0
5
10
15
20
25
30
Gestational weeks

(C)

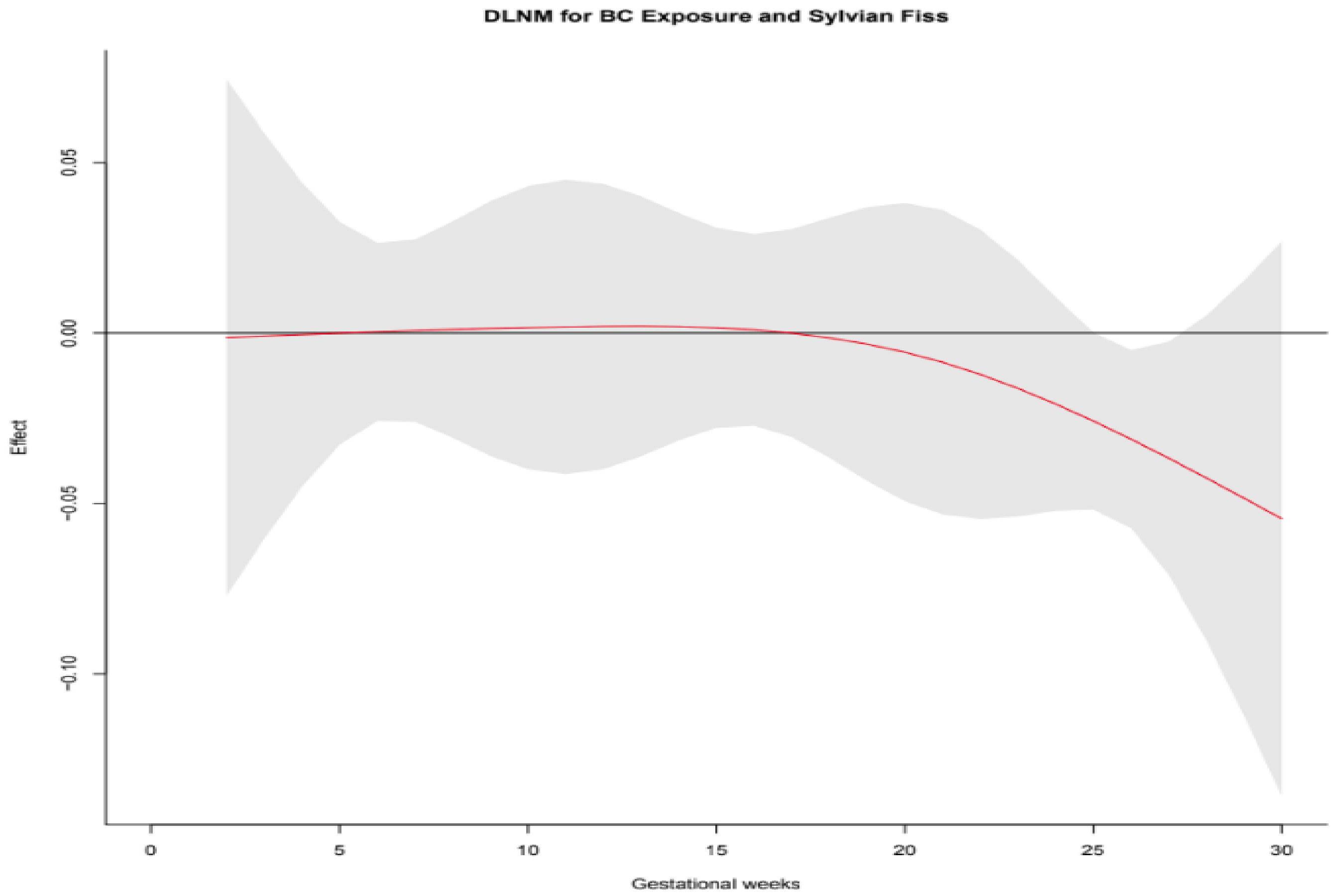

[a] Adjusted for foetal sex (boy vs girl), mother parity (multiparous vs nulliparous), maternal education (with university degree vs without university degree), ethnicity (European vs other), active smoking during pregnancy (no vs yes), passive smoking during pregnancy (no vs yes), alcohol consumption during pregnancy (no vs yes), and gestational age at ultrasound (days), and hospital and rater as random effects.

Abbreviations: NO_2, nitrogen dioxide; $PM_{2·5}$, particulate matter with an aerodynamic diameter < 2·5 µm; BC, black carbon; DLNM, Distributed Lag Non-linear Models.

Figure S4: Association of per IQR increases exposure to NO_2, $PM_{2·5}$, and BC with the Parieto-occipital sulcus depth during each week of pregnancy (weeks 1 to 30). [a] (A: NO_2, B: $PM_{2·5}$, C: BC)

(A)

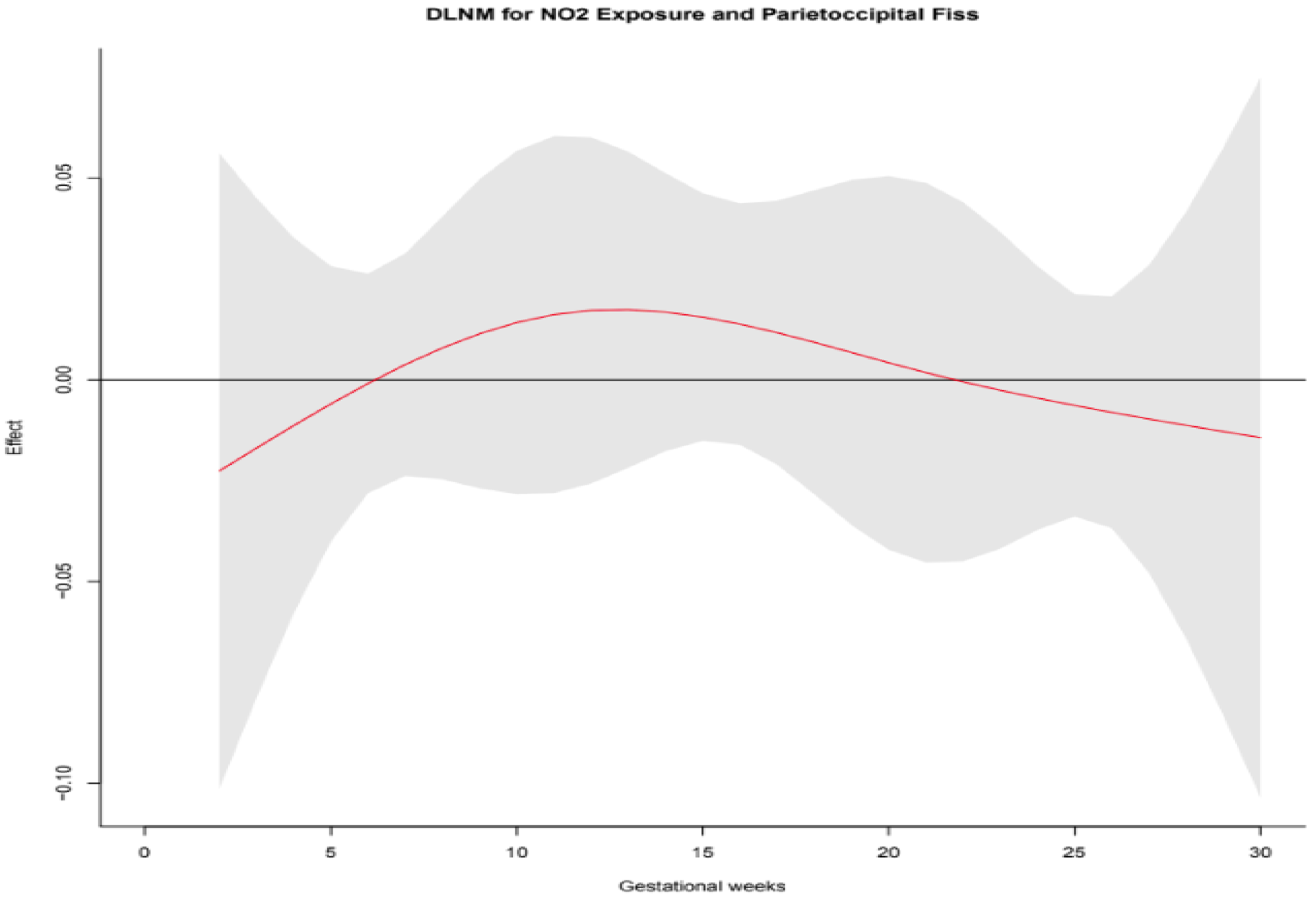

(B)

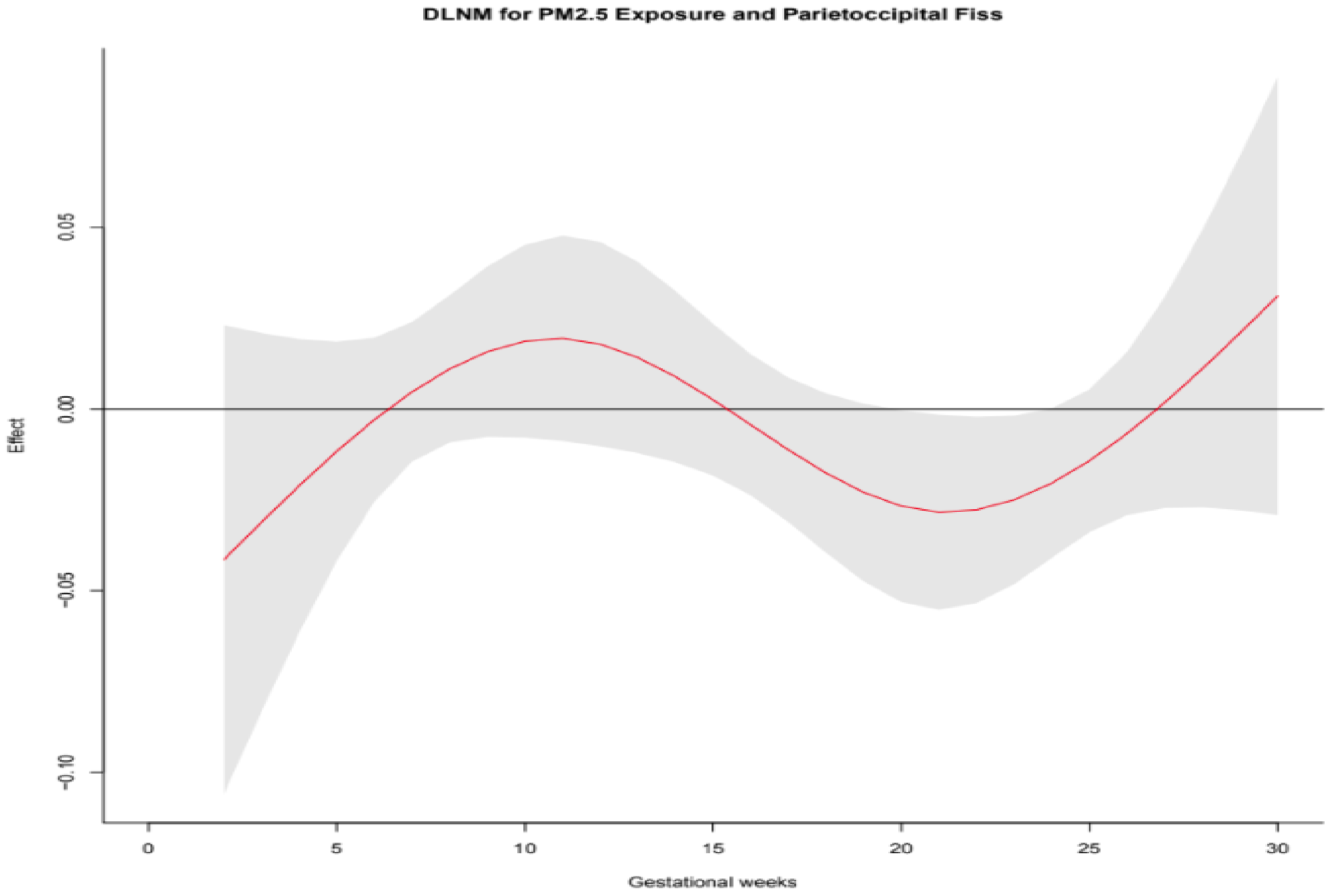
DLNM for PM2.5 Exposure and Parietoccipital Fiss
Effect
0.05
0.00
-0.05
-0.10
0
5
10
15
20
25
30
Gestational weeks

(C)

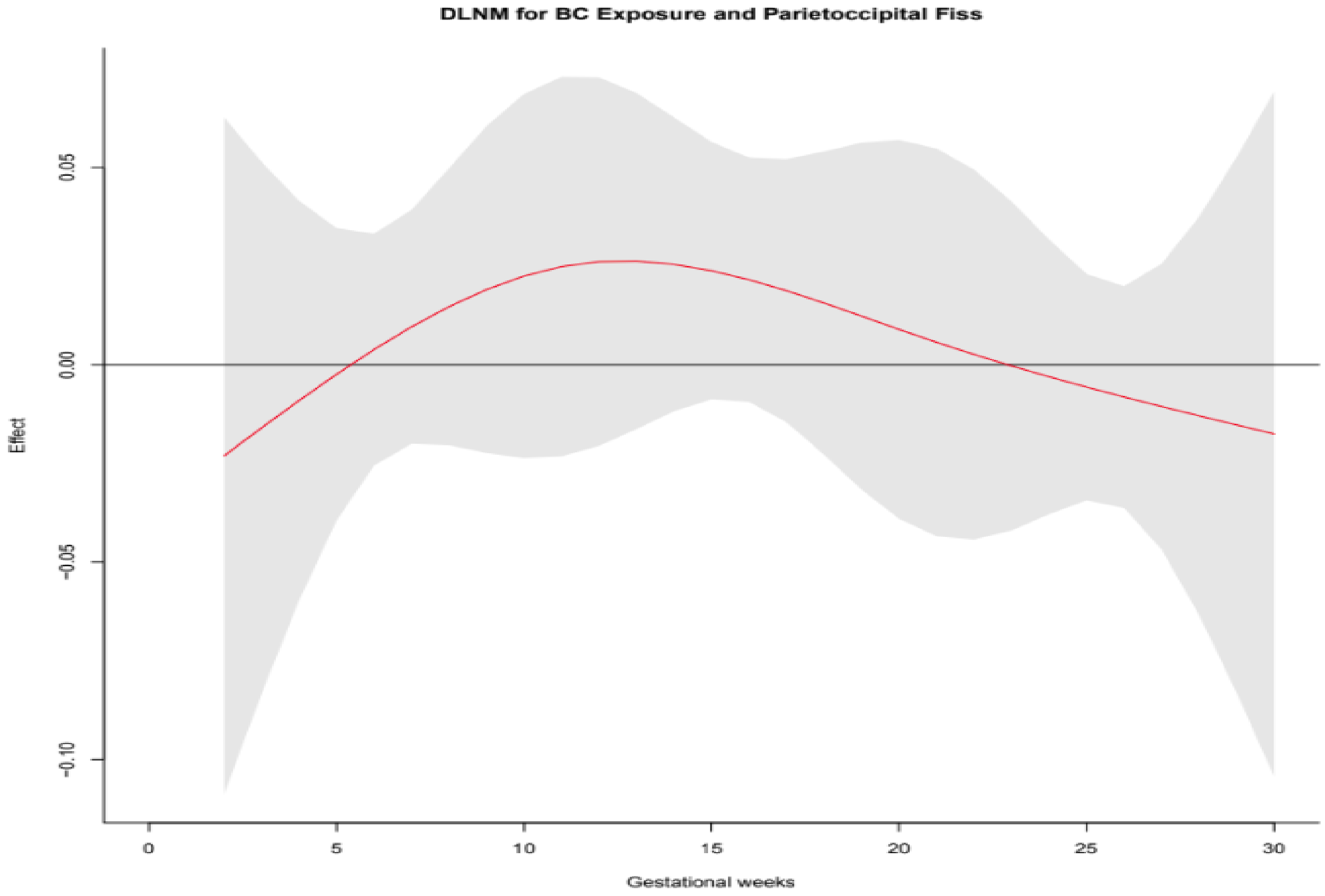

[a] Adjusted for foetal sex (boy vs girl), mother parity (multiparous vs nulliparous), maternal education (with university degree vs without university degree), ethnicity (European vs other), active smoking during pregnancy (no vs yes), passive smoking during pregnancy (no vs yes), alcohol consumption during pregnancy (no vs yes), and gestational age at ultrasound (days), and hospital and rater as random effects.

Abbreviations: NO_2, nitrogen dioxide; $PM_{2·5}$, particulate matter with an aerodynamic diameter < 2·5 µm; BC, black carbon; DLNM, Distributed Lag Non-linear Models.

Figure S5: Association of per IQR increases exposure to NO_2, $PM_{2·5}$, and BC with the Cingulate sulcus depth during each week of pregnancy (weeks 1 to 30). [a] (A: NO_2, B: $PM_{2·5}$, C: BC)

(A)

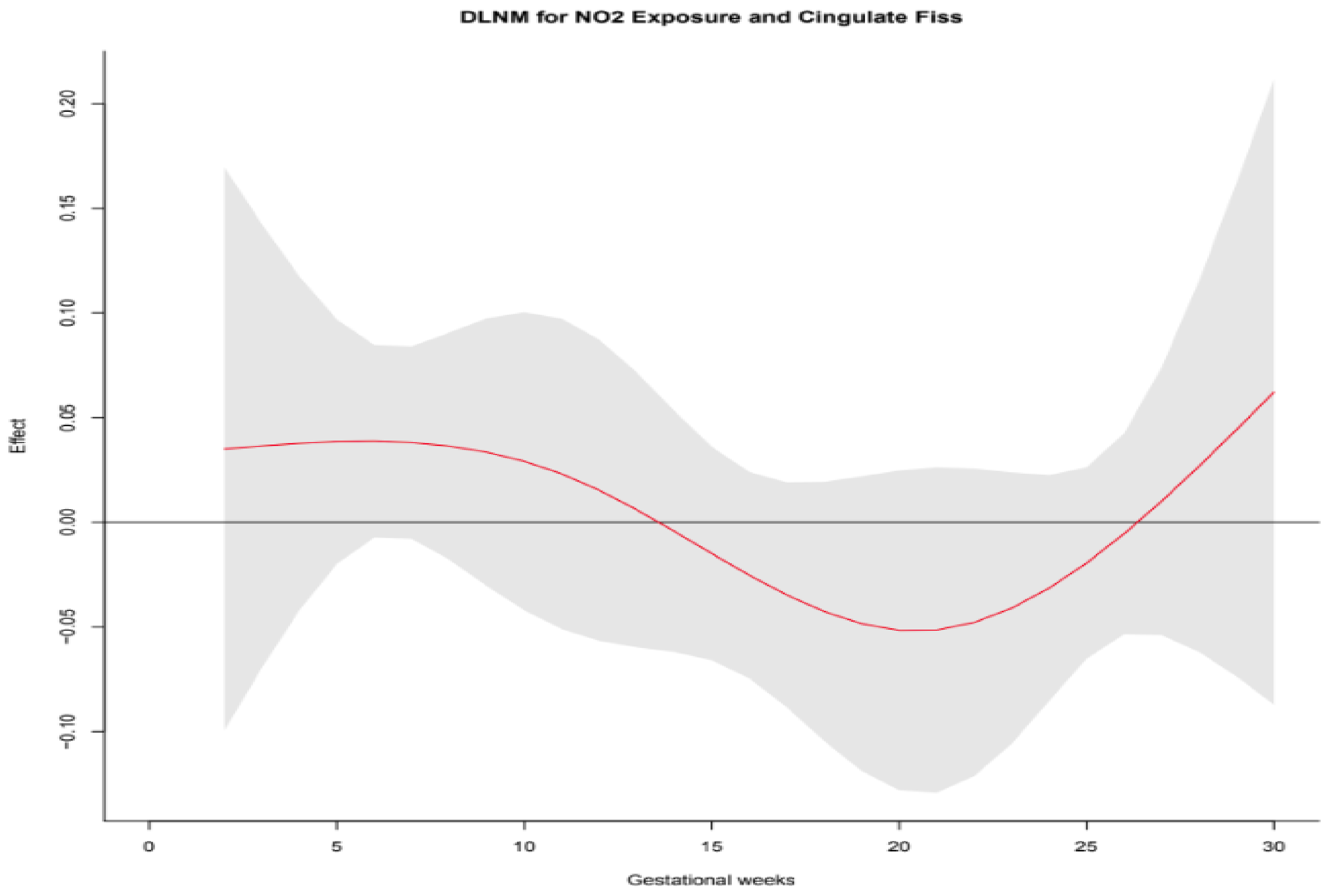

(B)

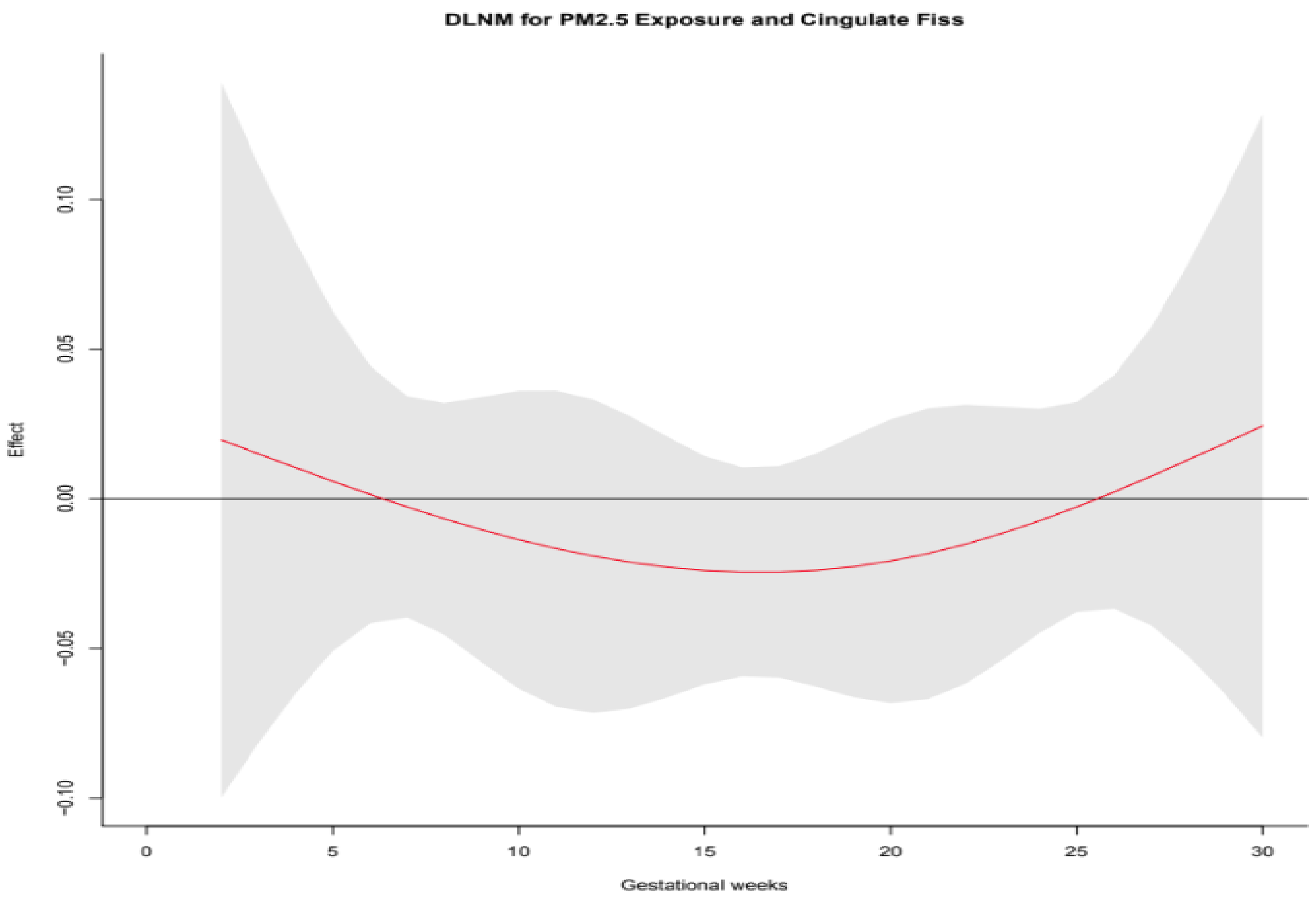
DLNM for PM2.5 Exposure and Cingulate Fiss
Effect
0.10
0.05
0.00
−0.05
−0.10
0
5
10
15
20
25
30
Gestational weeks

(C)

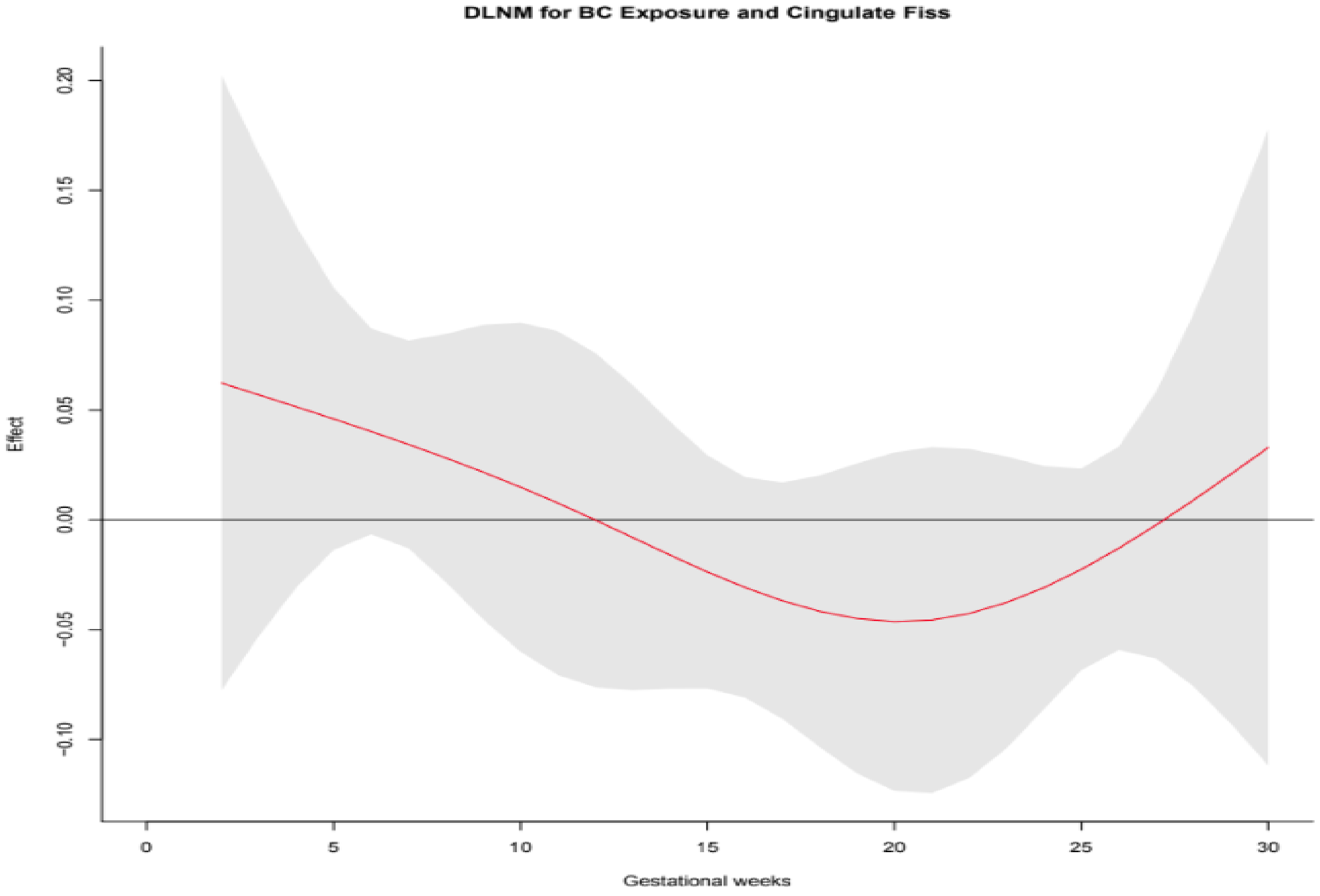

[a] Adjusted for foetal sex (boy vs girl), mother parity (multiparous vs nulliparous), maternal education (with university degree vs without university degree), ethnicity (European vs other), active smoking during pregnancy (no vs yes), passive smoking during pregnancy (no vs yes), alcohol consumption during pregnancy (no vs yes), and gestational age at ultrasound (days), and hospital and rater as random effects.

Abbreviations: NO_2, nitrogen dioxide; $PM_{2\cdot5}$, particulate matter with an aerodynamic diameter < 2·5 µm; BC, black carbon; DLNM, Distributed Lag Non-linear Models.

Figure S6: Association of per IQR increases exposure to NO_2, $PM_{2\cdot5}$, and BC with the Calcarine sulcus depth during each week of pregnancy (weeks 1 to 30). [a] (A: NO_2, B: $PM_{2\cdot5}$, C: BC)

(A)

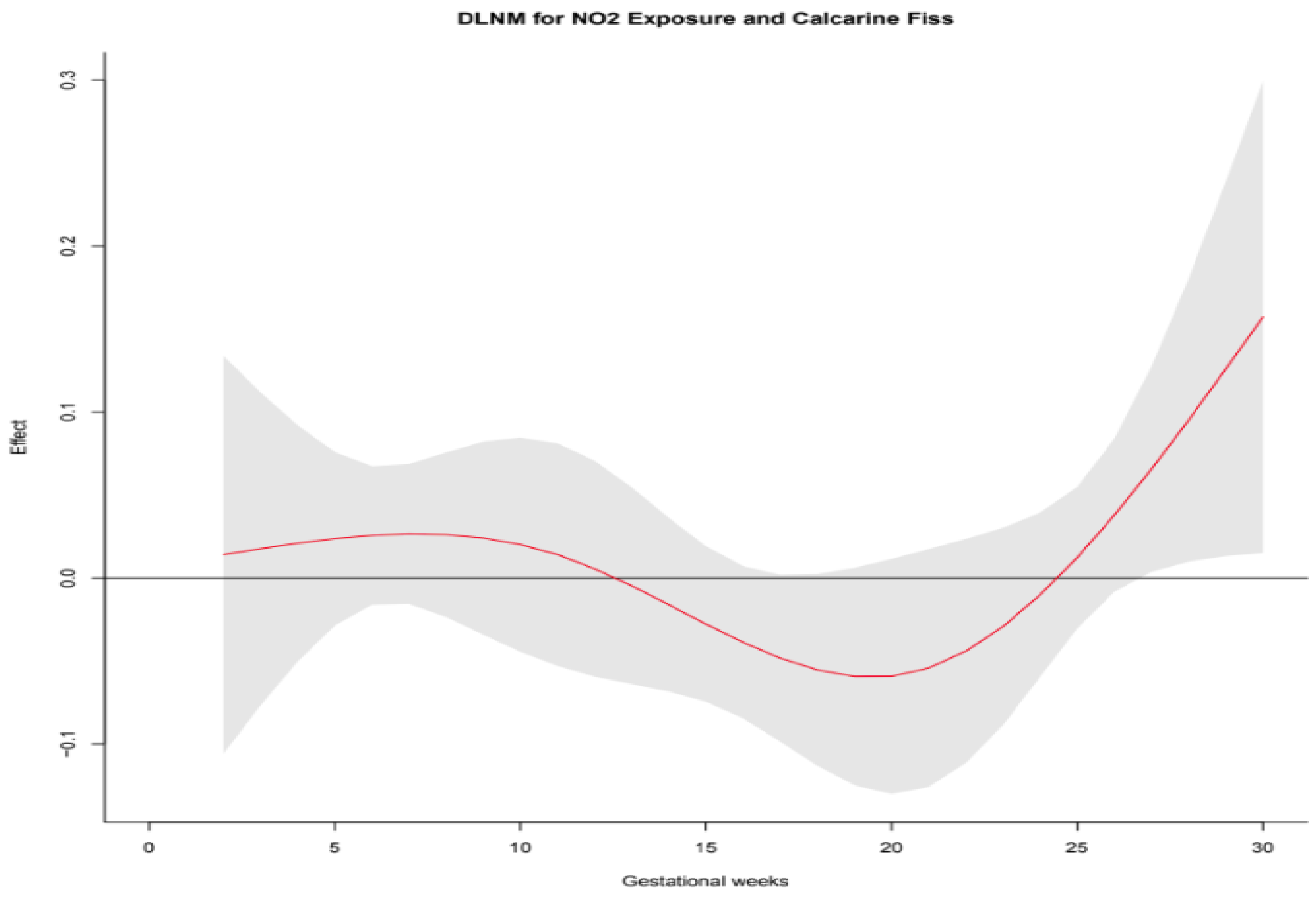

(B)

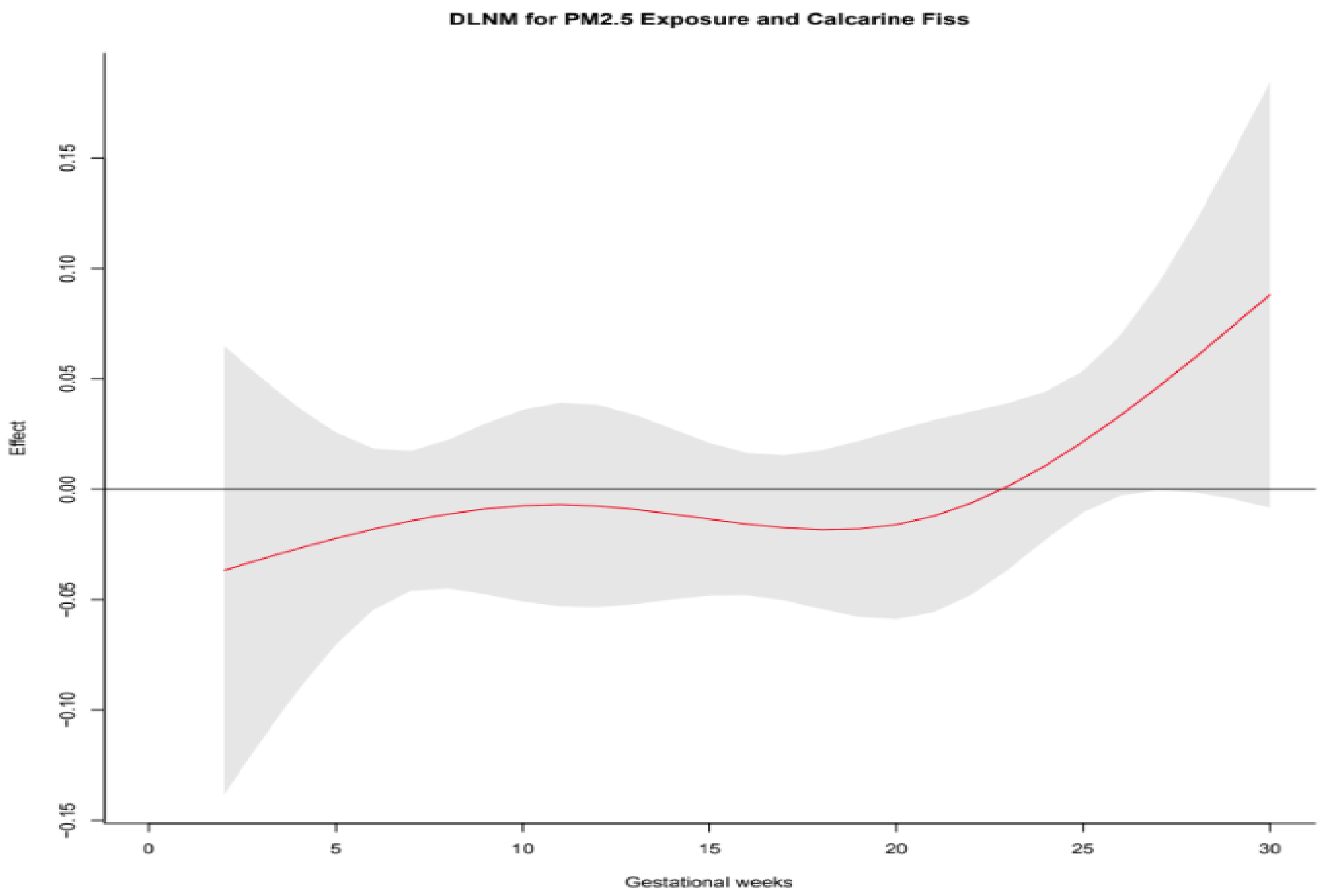
DLNM for PM2.5 Exposure and Calcarine Fiss
Effect
0.15
0.10
0.05
0.00
−0.05
−0.10
−0.15
0
5
10
15
20
25
30
Gestational weeks

(C)

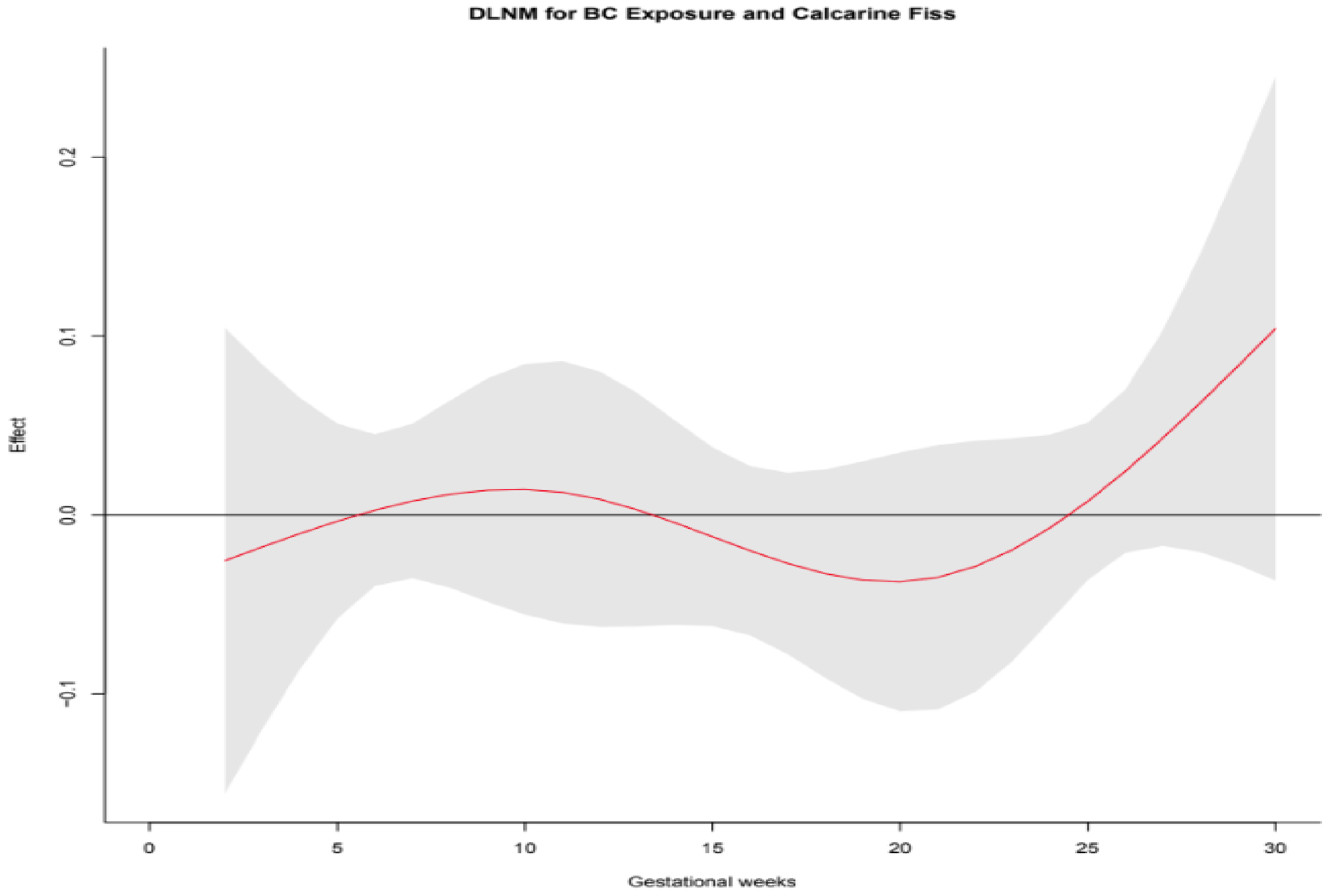

[a] Adjusted for foetal sex (boy vs girl), mother parity (multiparous vs nulliparous), maternal education (with university degree vs without university degree), ethnicity (European vs other), active smoking during pregnancy (no vs yes), passive smoking during pregnancy (no vs yes), alcohol consumption during pregnancy (no vs yes), and gestational age at ultrasound (days), and hospital and rater as random effects.

Abbreviations: NO_2, nitrogen dioxide; $PM_{2·5}$, particulate matter with an aerodynamic diameter < 2·5 µm; BC, black carbon; DLNM, Distributed Lag Non-linear Models.

Figure S7: Association of per IQR increases exposure to NO_2, $PM_{2\cdot5}$, and BC with the anterior lateral ventricle width during each week of pregnancy (weeks 1 to 30). [a] (A: NO_2, B: $PM_{2\cdot5}$, C: BC)

(A)

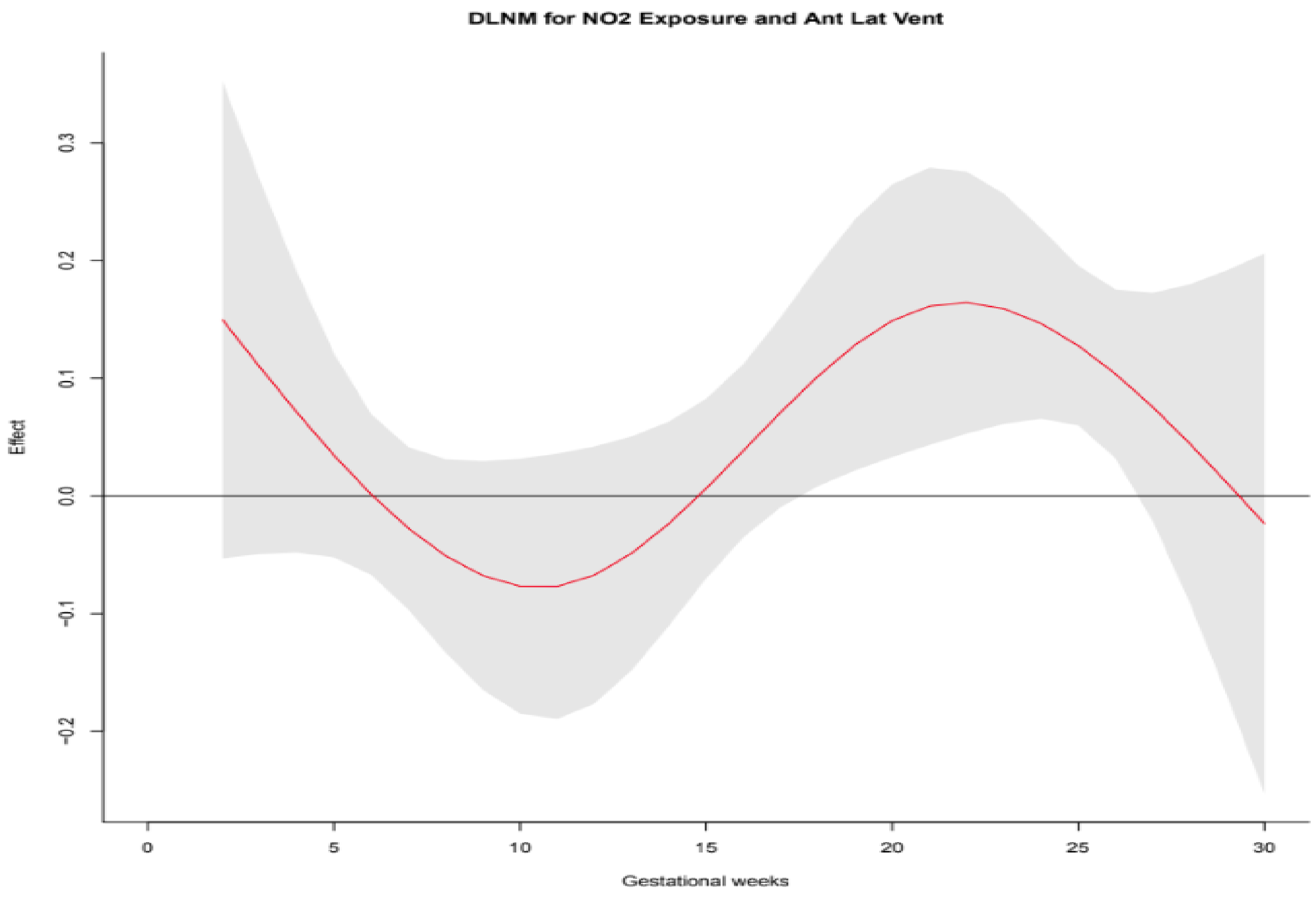

(B)

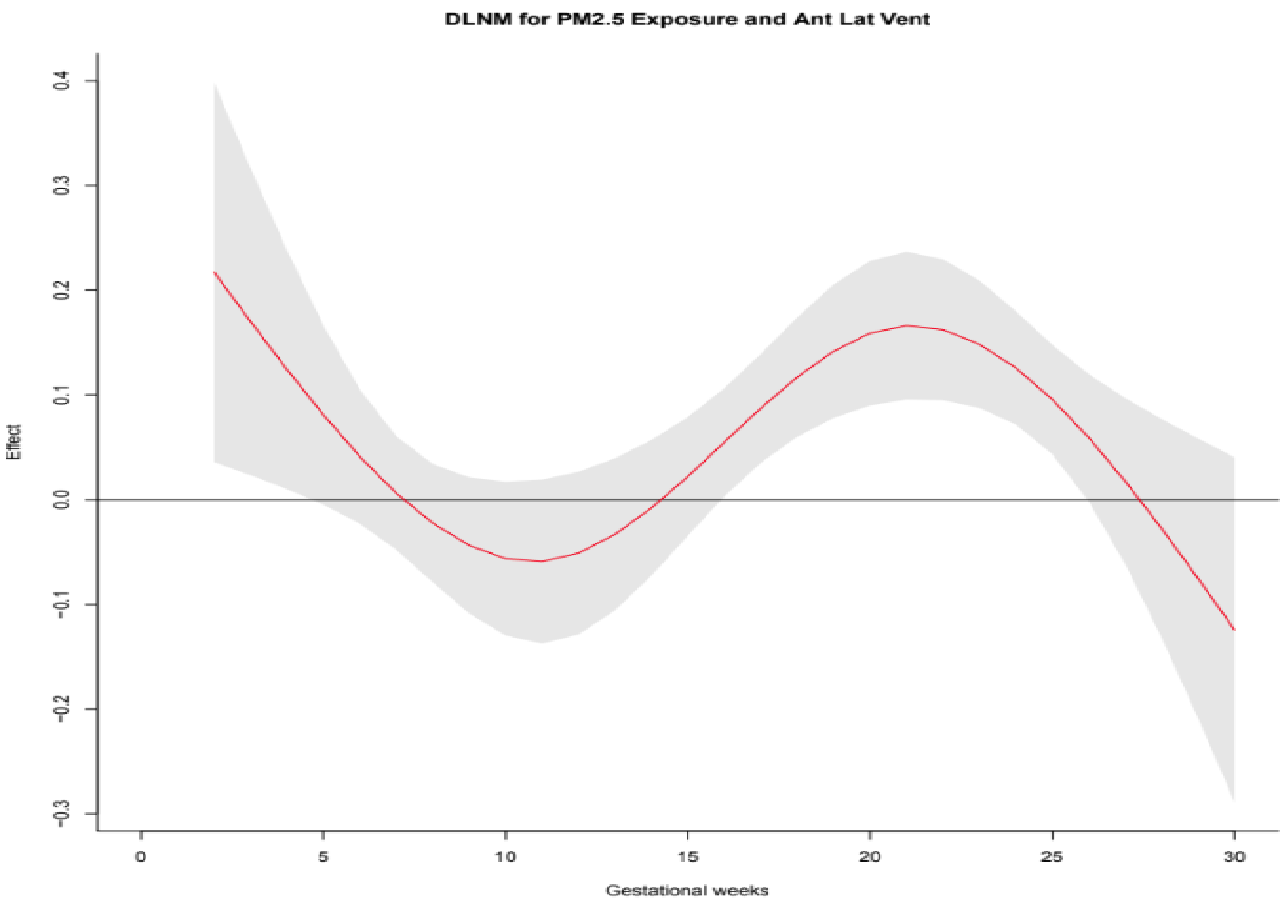
DLNM for PM2.5 Exposure and Ant Lat Vent
Effect
0.4
0.3
0.2
0.1
0.0
-0.1
-0.2
-0.3
0
5
10
15
20
25
30
Gestational weeks

(C)

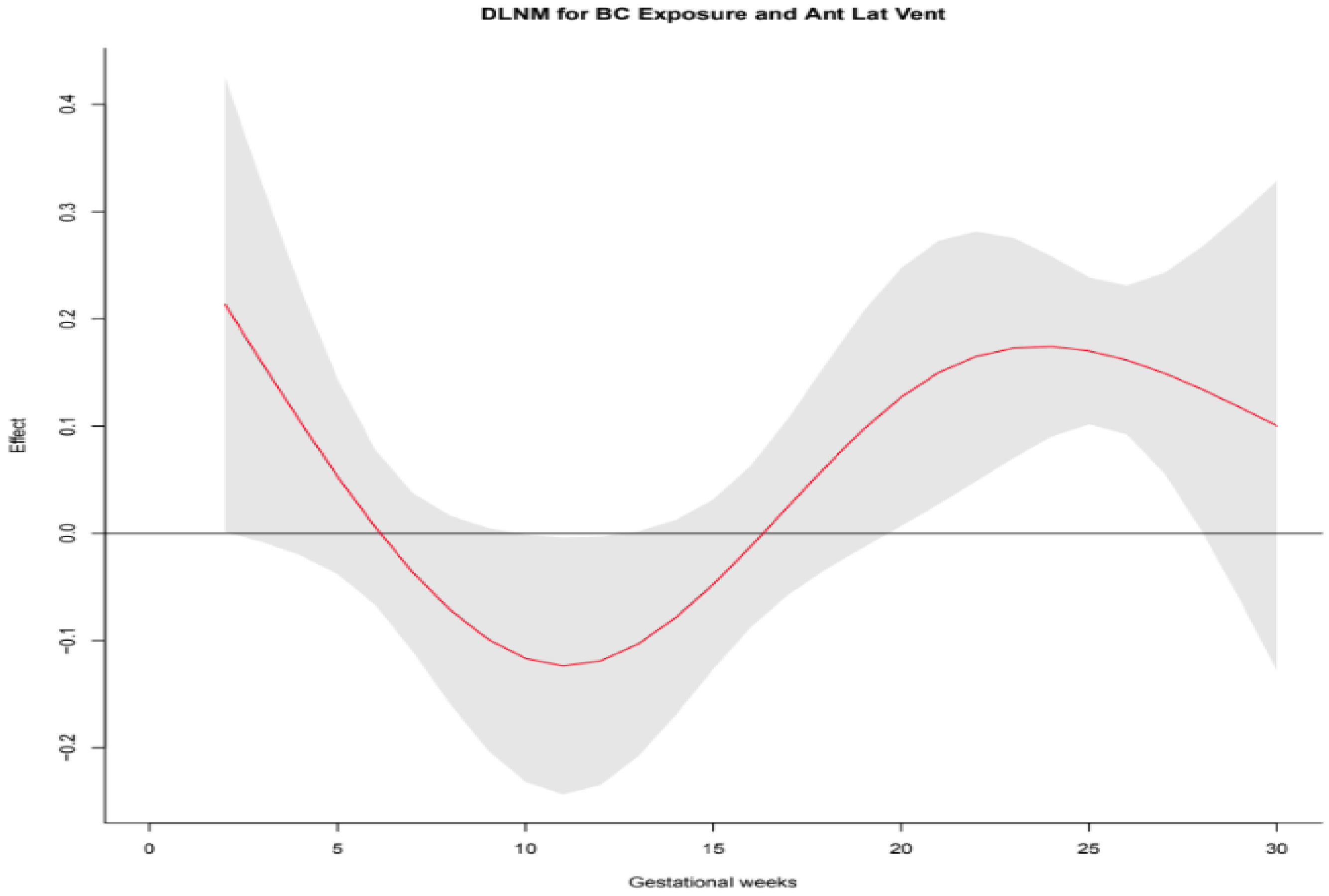

[a] Adjusted for foetal sex (boy vs girl), mother parity (multiparous vs nulliparous), maternal education (with university degree vs without university degree), ethnicity (European vs other), active smoking during pregnancy (no vs yes), passive smoking during pregnancy (no vs yes), alcohol consumption during pregnancy (no vs yes), and gestational age at ultrasound (days), and hospital and rater as random effects.

Abbreviations: NO_2, nitrogen dioxide; $PM_{2·5}$, particulate matter with an aerodynamic diameter < 2·5 µm; BC, black carbon; DLNM, Distributed Lag Non-linear Models.

Figure S8: Association of per IQR increases exposure to NO_2, $PM_{2.5}$, and BC with the posterior lateral ventricle width during each week of pregnancy (weeks 1 to 30). [a] (A: NO_2, B: $PM_{2.5}$, C: BC)

(A)

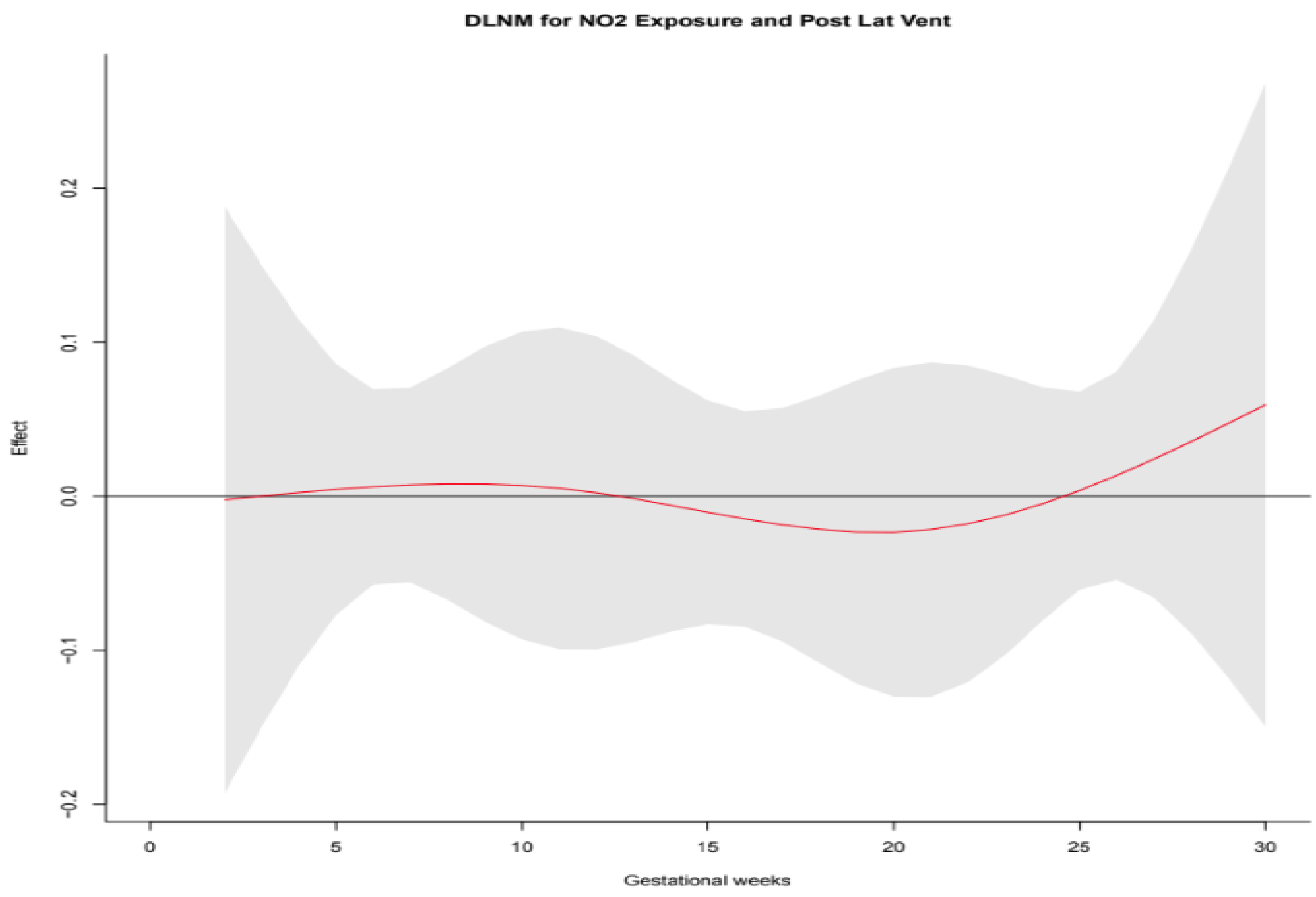

(B)

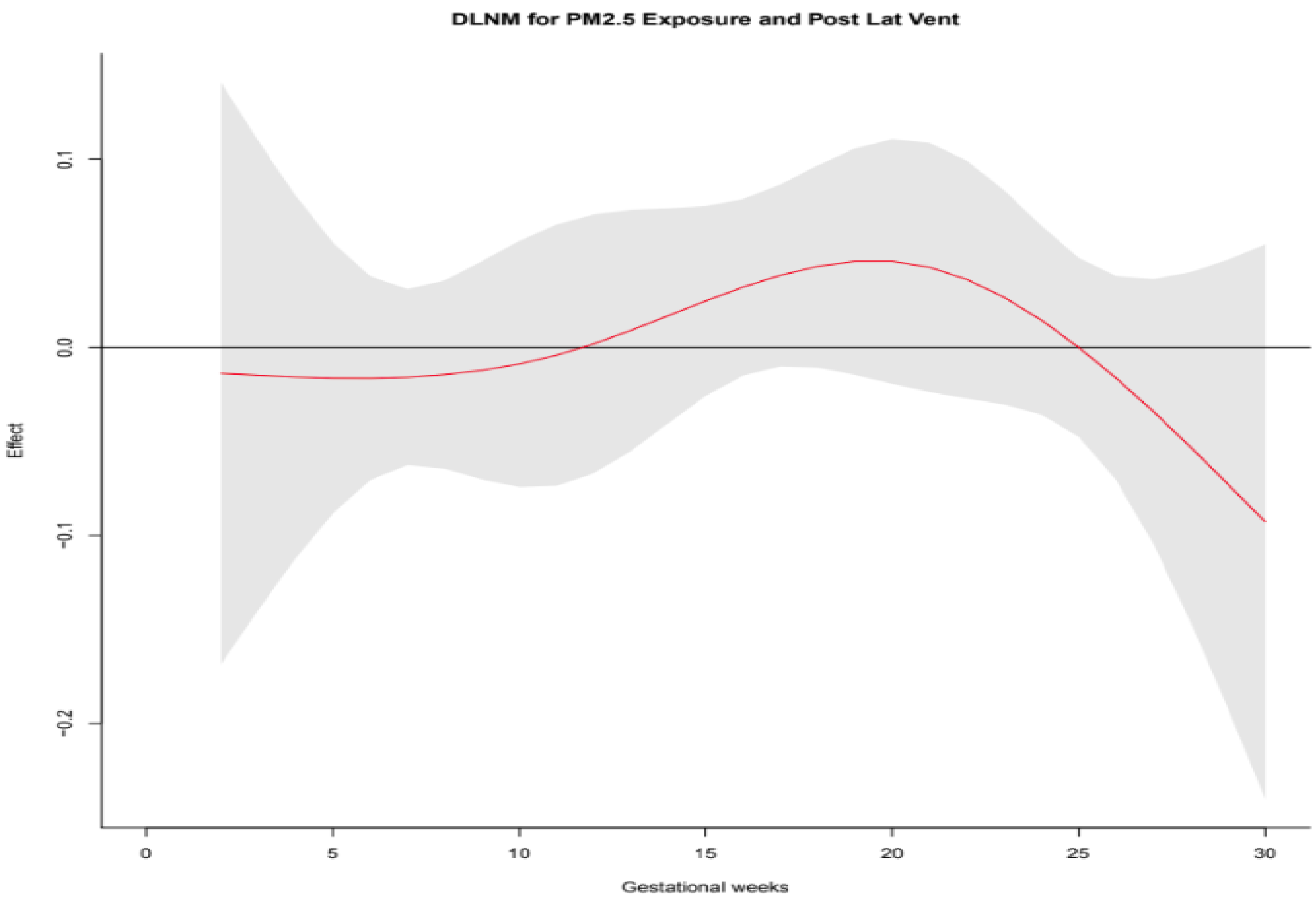
DLNM for PM2.5 Exposure and Post Lat Vent
Effect
0.1
0.0
-0.1
-0.2
0
5
10
15
20
25
30
Gestational weeks

(C)

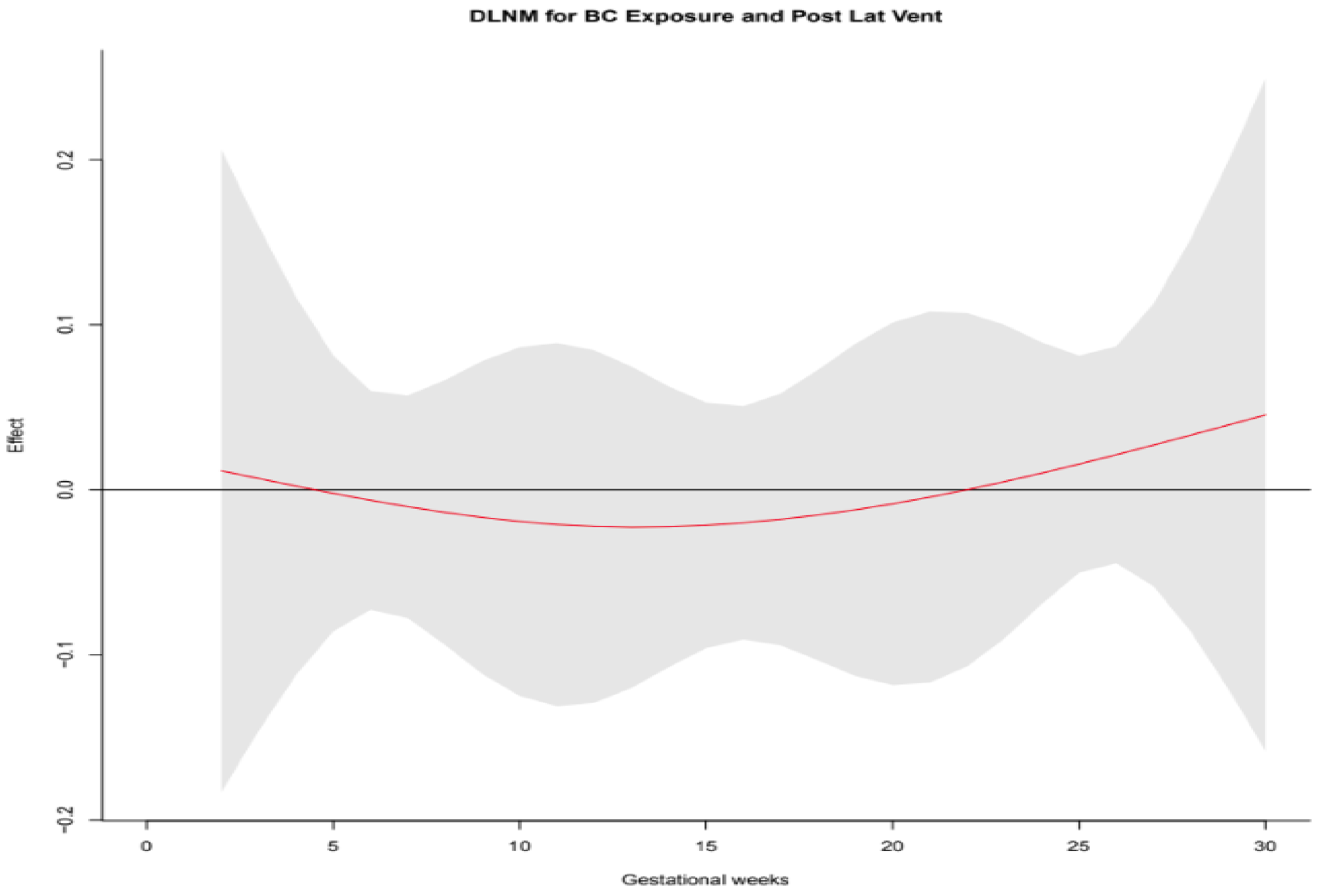

[a] Adjusted for foetal sex (boy vs girl), mother parity (multiparous vs nulliparous), maternal education (with university degree vs without university degree), ethnicity (European vs other), active smoking during pregnancy (no vs yes), passive smoking during pregnancy (no vs yes), alcohol consumption during pregnancy (no vs yes), and gestational age at ultrasound (days), and hospital and rater as random effects.

Abbreviations: NO_2, nitrogen dioxide; $PM_{2\cdot5}$, particulate matter with an aerodynamic diameter < 2·5 µm; BC, black carbon; DLNM, Distributed Lag Non-linear Models.

Figure S9: Association of per IQR increases exposure to NO_2, $PM_{2·5}$, and BC with the third ventricle width during each week of pregnancy (weeks 1 to 30). [a] (A: NO_2, B: $PM_{2·5}$, C: BC)

(A)

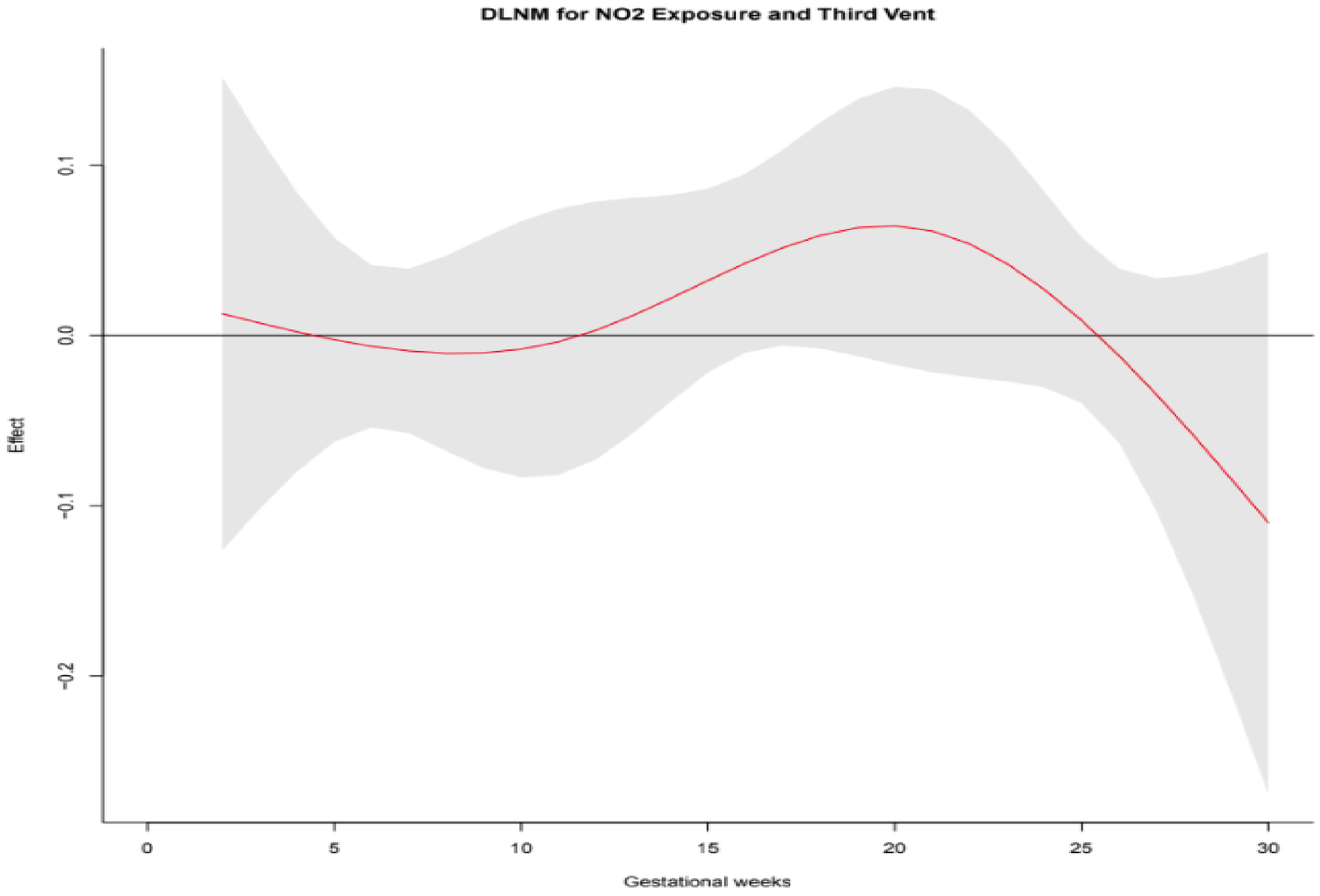

(B)

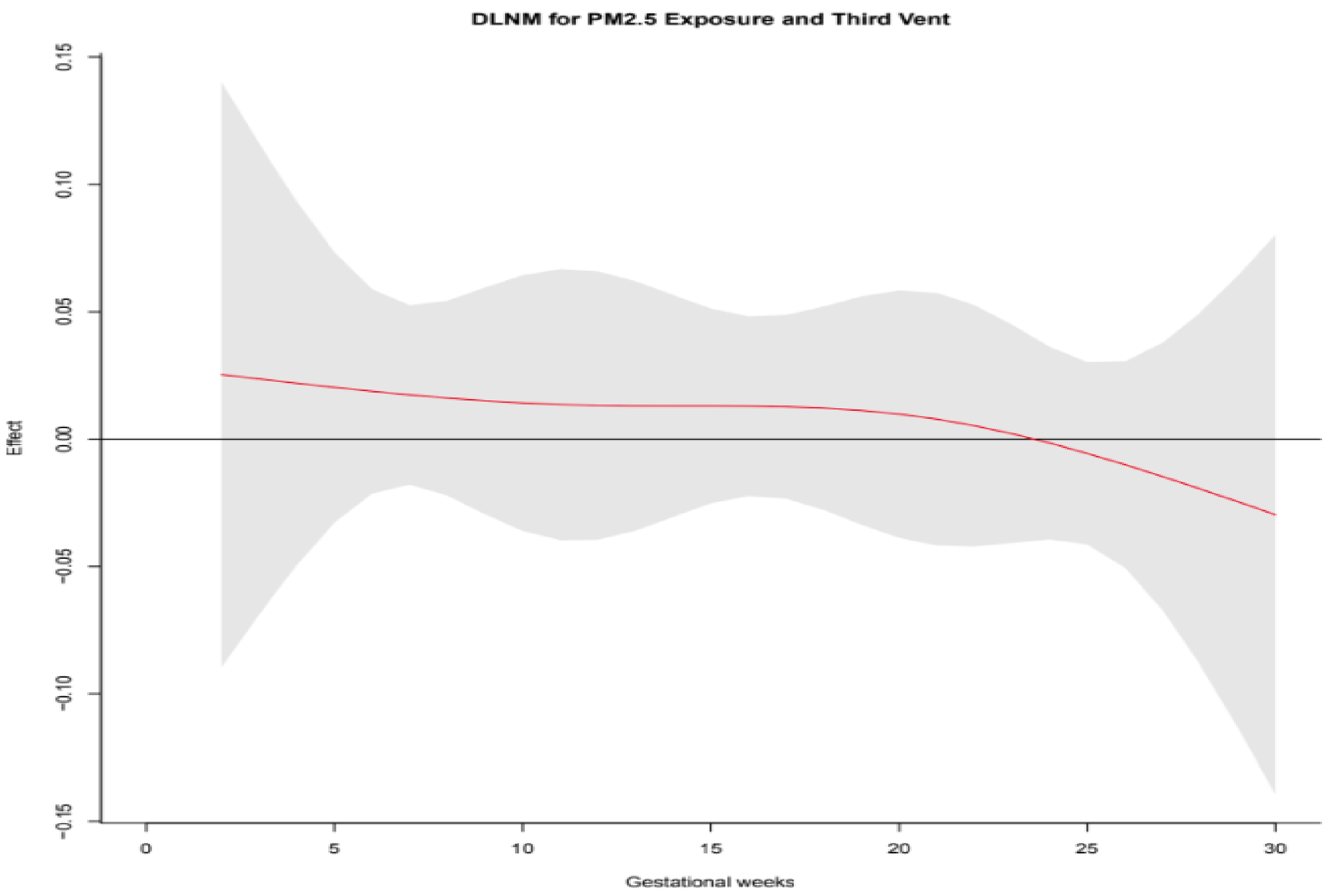
DLNM for PM2.5 Exposure and Third Vent
Effect
0.15
0.10
0.05
0.00
−0.05
−0.10
−0.15
0
5
10
15
20
25
30
Gestational weeks

(C)

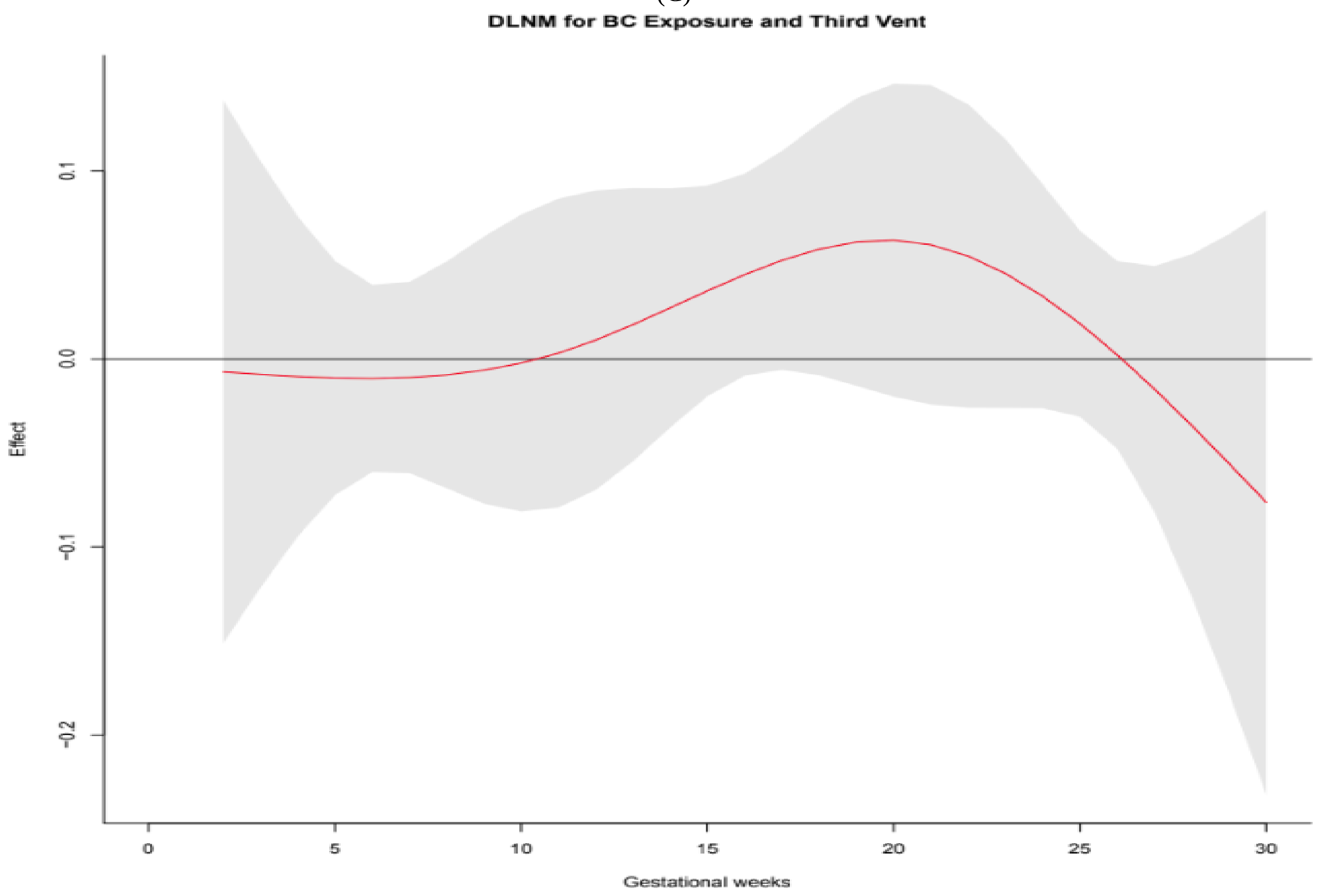

[a] Adjusted for foetal sex (boy vs girl), mother parity (multiparous vs nulliparous), maternal education (with university degree vs without university degree), ethnicity (European vs other), active smoking during pregnancy (no vs yes), passive smoking during pregnancy (no vs yes), alcohol consumption during pregnancy (no vs yes), and gestational age at ultrasound (days), and hospital and rater as random effects.

Abbreviations: NO_2, nitrogen dioxide; $PM_{2·5}$, particulate matter with an aerodynamic diameter < 2·5 µm; BC, black carbon; DLNM, Distributed Lag Non-linear Models.

Figure S10: Association of per IQR increases exposure to NO_2, $PM_{2\cdot5}$, and BC with the cisterna magna width during each week of pregnancy (weeks 1 to 30). [a] (A: NO_2, B: $PM_{2\cdot5}$, C: BC)

(A)

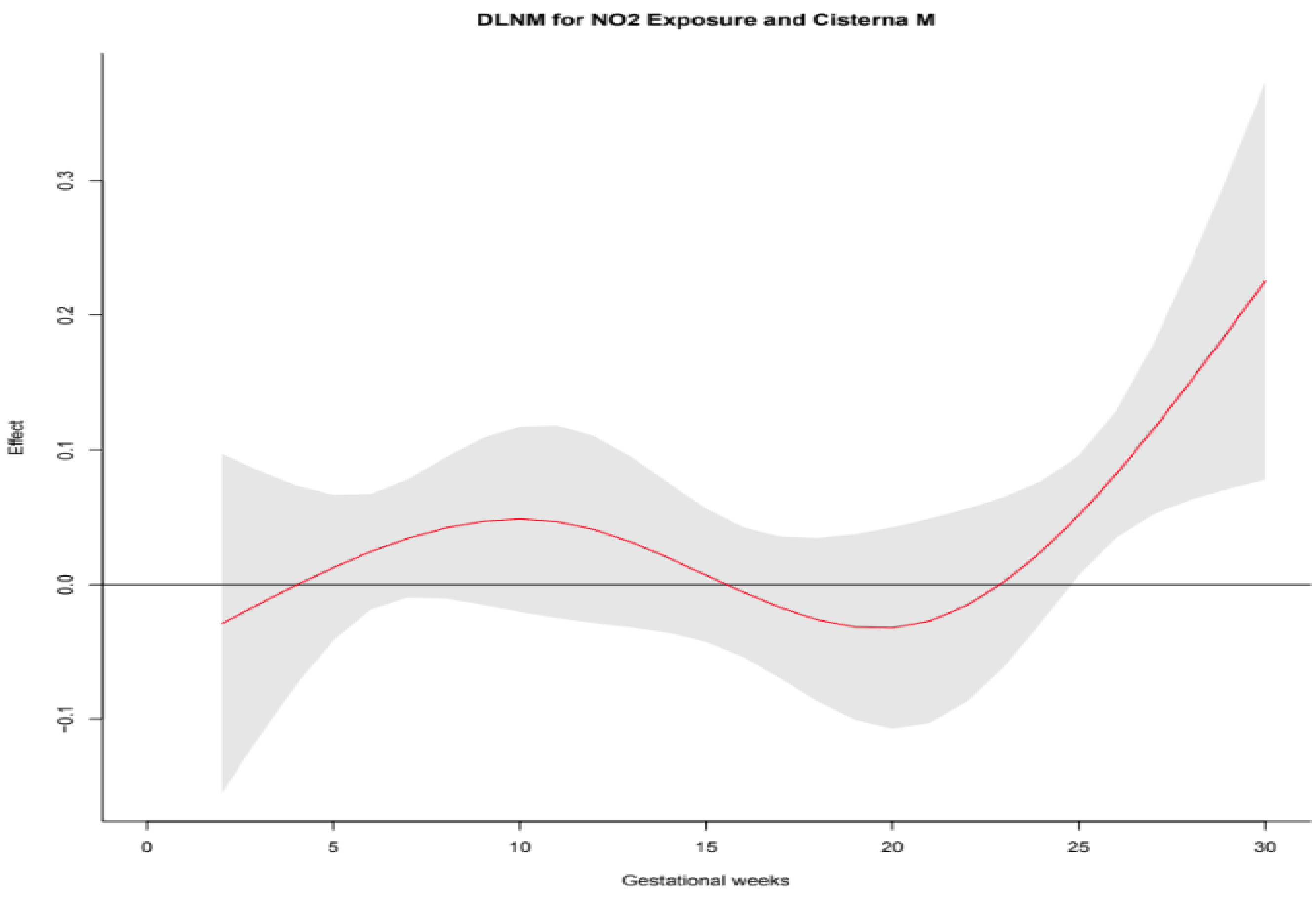

(B)

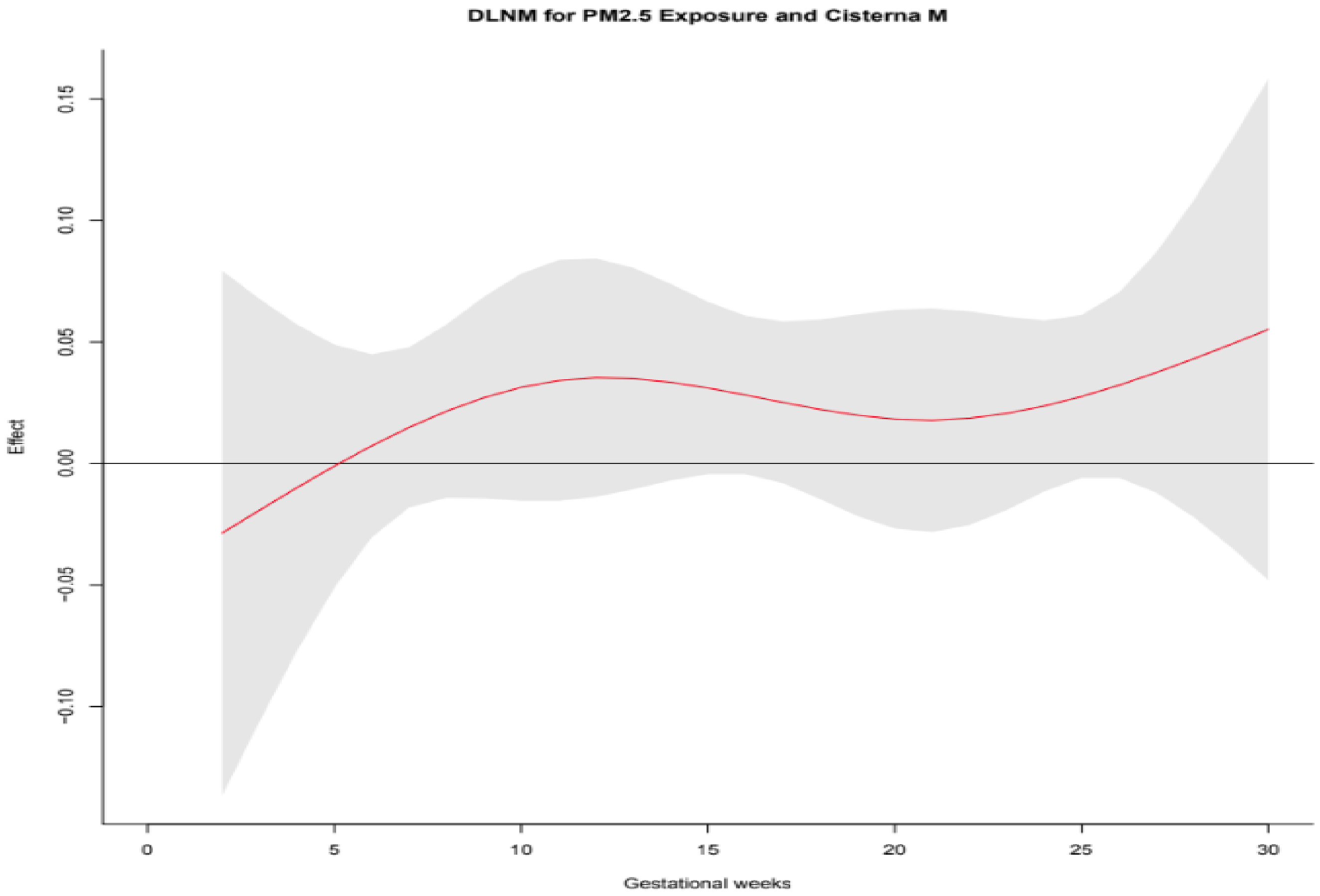
DLNM for PM2.5 Exposure and Cisterna M
Effect
0.15
0.10
0.05
0.00
-0.05
-0.10
0
5
10
15
20
25
30
Gestational weeks

(C)

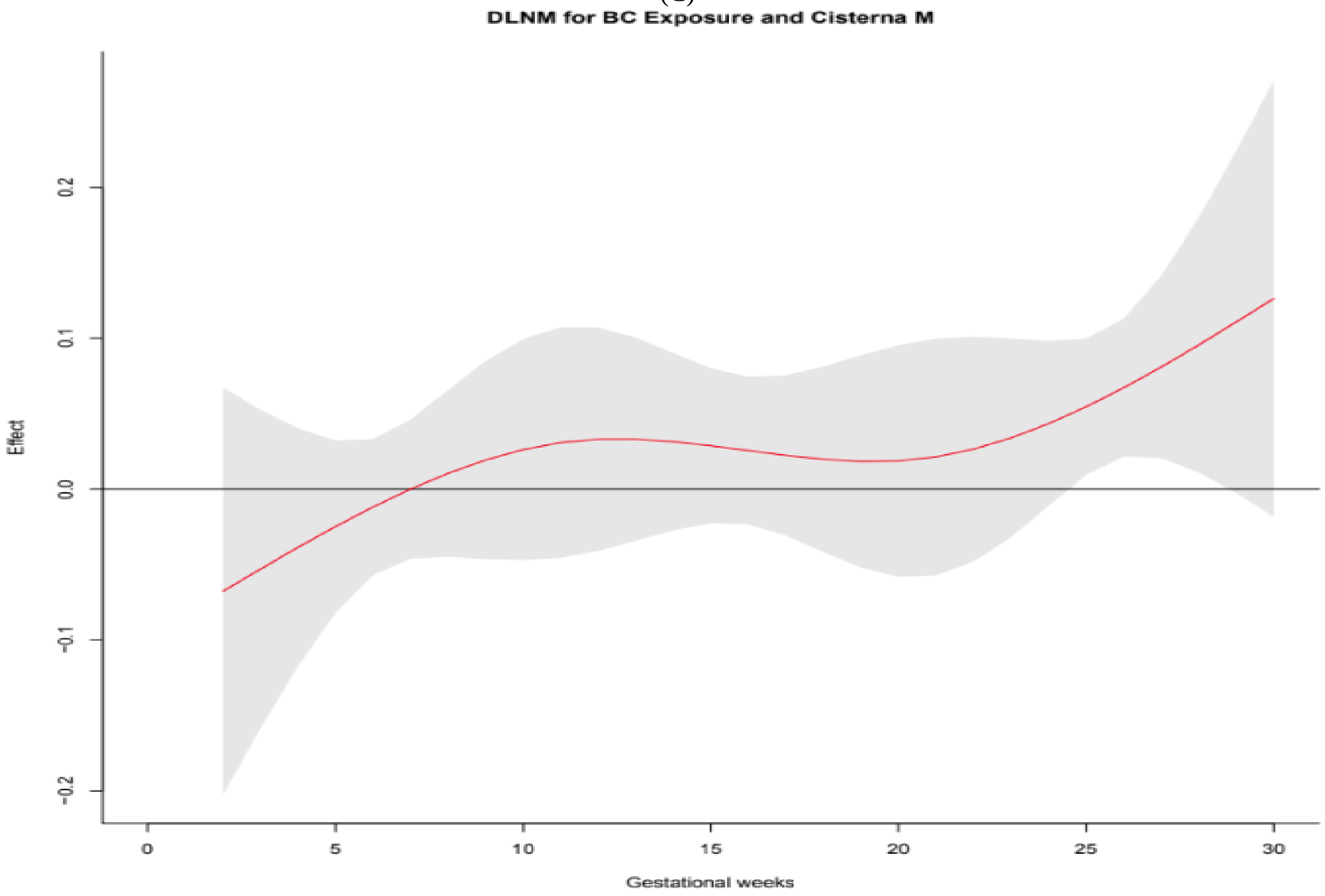

[a] Adjusted for foetal sex (boy vs girl), mother parity (multiparous vs nulliparous), maternal education (with university degree vs without university degree), ethnicity (European vs other), active smoking during pregnancy (no vs yes), passive smoking during pregnancy (no vs yes), alcohol consumption during pregnancy (no vs yes), and gestational age at ultrasound (days), and hospital and rater as random effects.

Abbreviations: NO_2, nitrogen dioxide; $PM_{2·5}$, particulate matter with an aerodynamic diameter < 2·5 µm; BC, black carbon; DLNM, Distributed Lag Non-linear Models.

Figure S11: Association of per IQR increases exposure to NO_2, $PM_{2\cdot5}$, and BC with the corpus callosum length during each week of pregnancy (weeks 1 to 30). [a] (A: NO_2, B: $PM_{2\cdot5}$, C: BC)

(A)

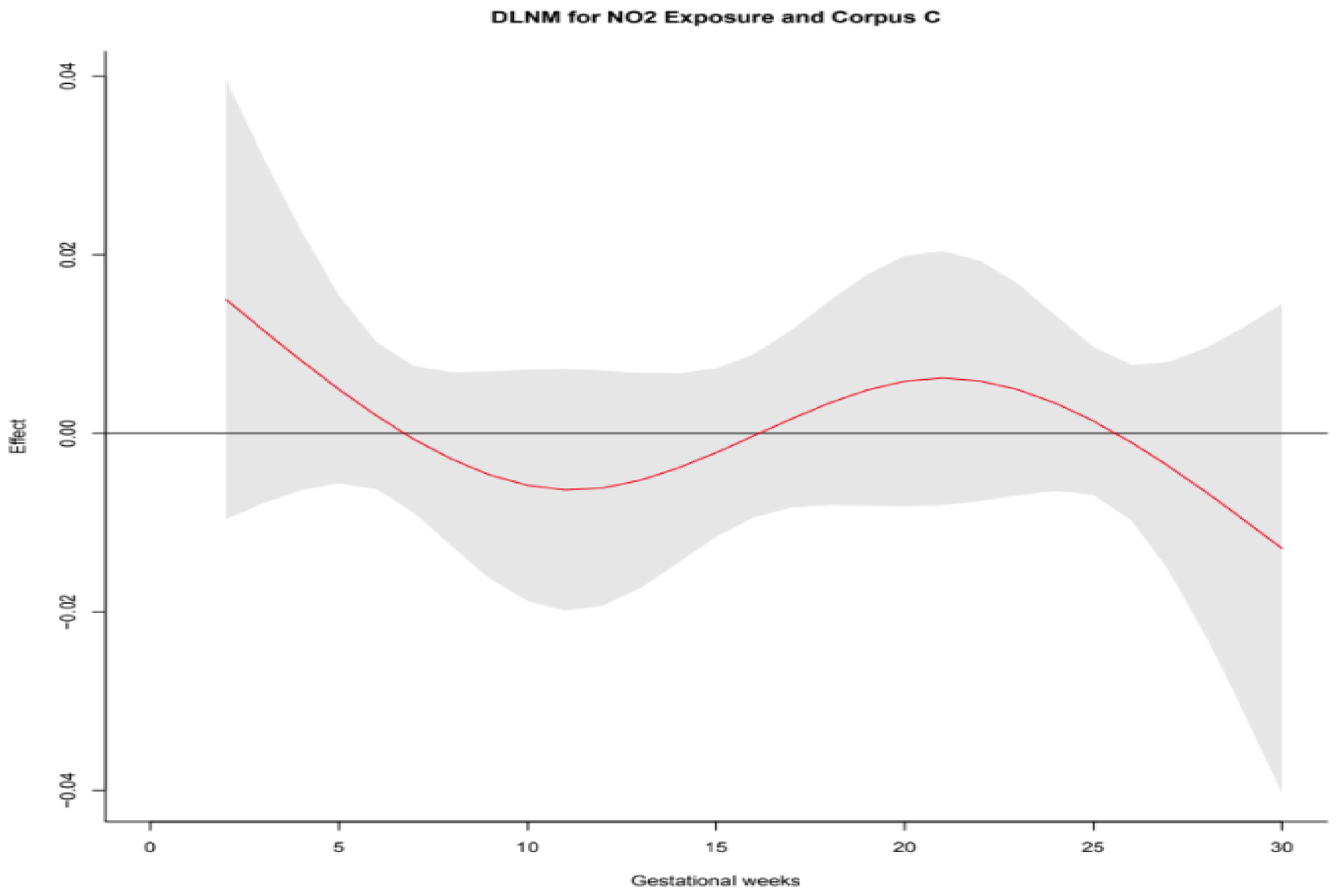

(B)

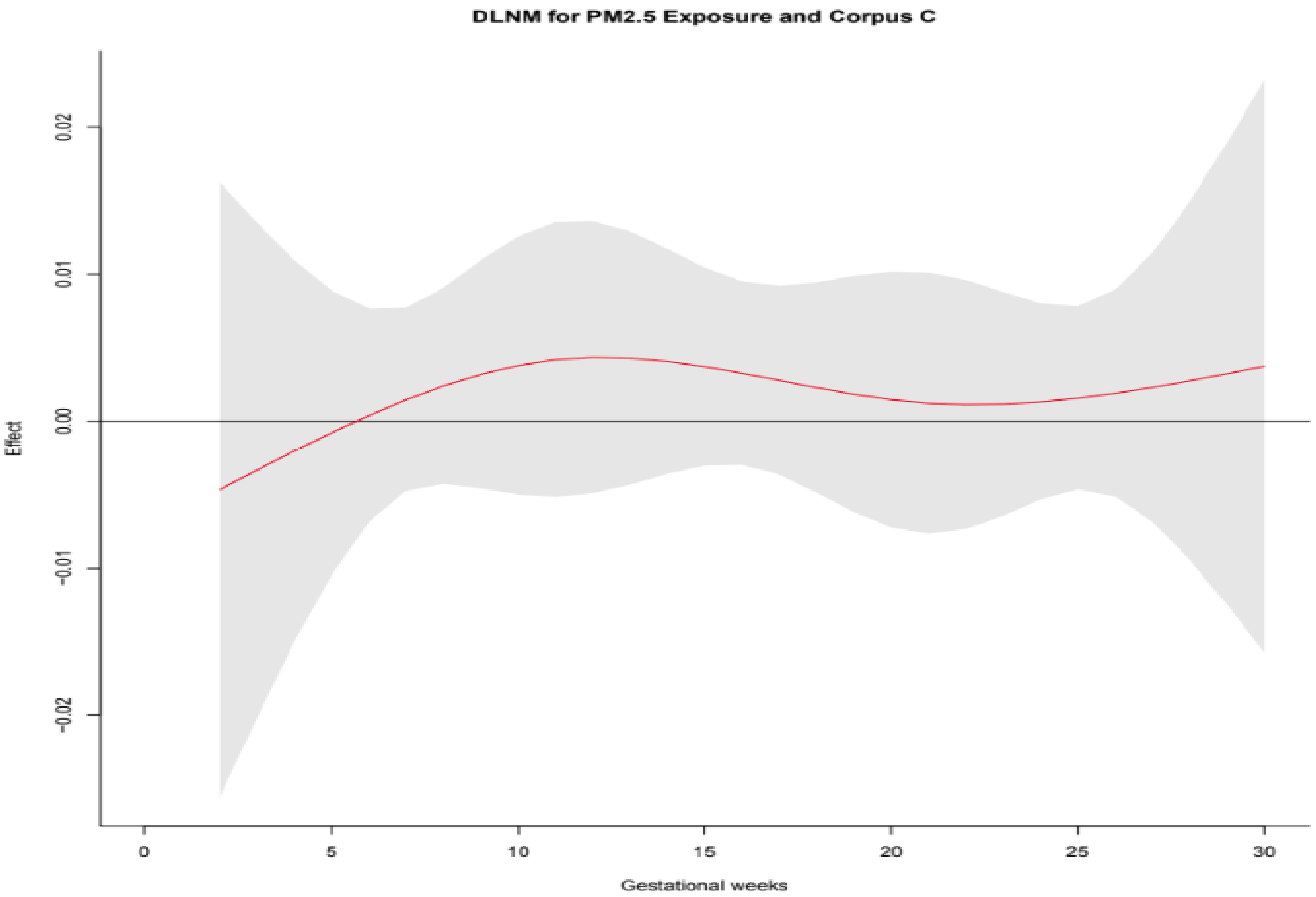
DLNM for PM2.5 Exposure and Corpus C
Effect
0.02
0.01
0.00
-0.01
-0.02
0
5
10
15
20
25
30
Gestational weeks

(C)

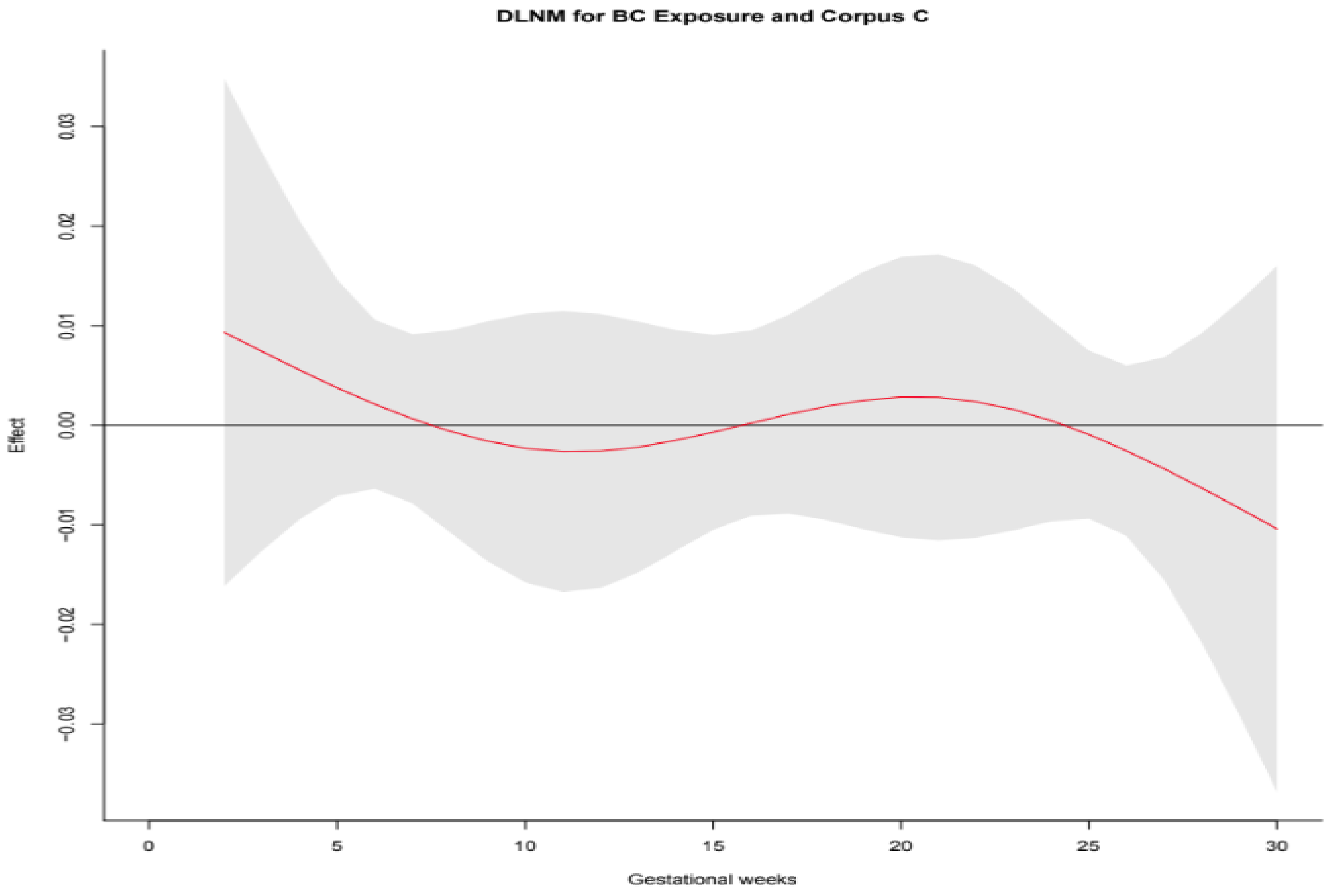

[a] Adjusted for foetal sex (boy vs girl), mother parity (multiparous vs nulliparous), maternal education (with university degree vs without university degree), ethnicity (European vs other), active smoking during pregnancy (no vs yes), passive smoking during pregnancy (no vs yes), alcohol consumption during pregnancy (no vs yes), and gestational age at ultrasound (days), and hospital and rater as random effects.

Abbreviations: NO_2, nitrogen dioxide; $PM_{2·5}$, particulate matter with an aerodynamic diameter < 2·5 µm; BC, black carbon; DLNM, Distributed Lag Non-linear Models.

Figure S12: Association of per IQR increases exposure to NO_2, $PM_{2 \cdot 5}$, and BC with the cerebellar vermis height during each week of pregnancy (weeks 1 to 30). [a] (A: NO_2, B: $PM_{2 \cdot 5}$, C: BC)

(A)

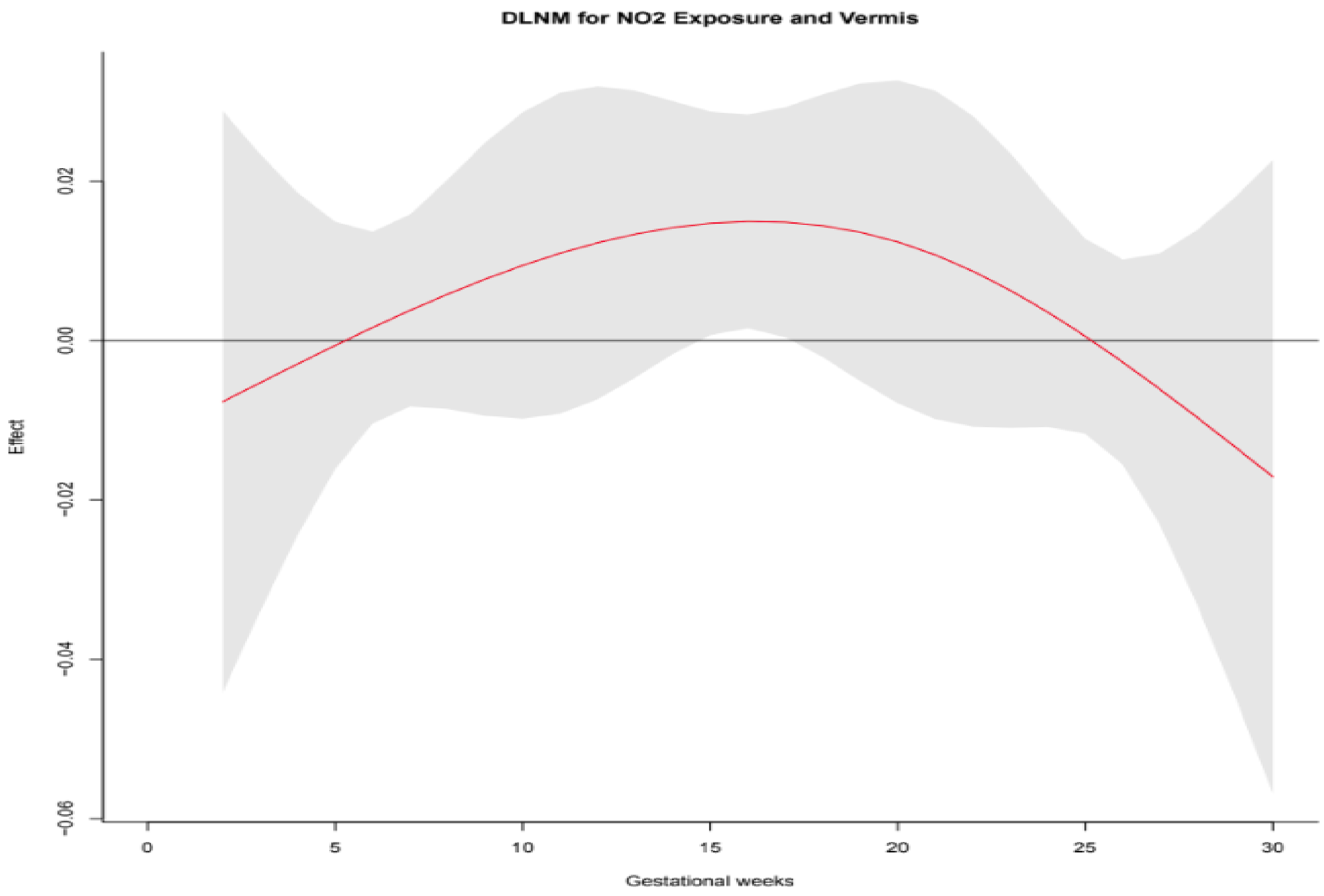

(B)

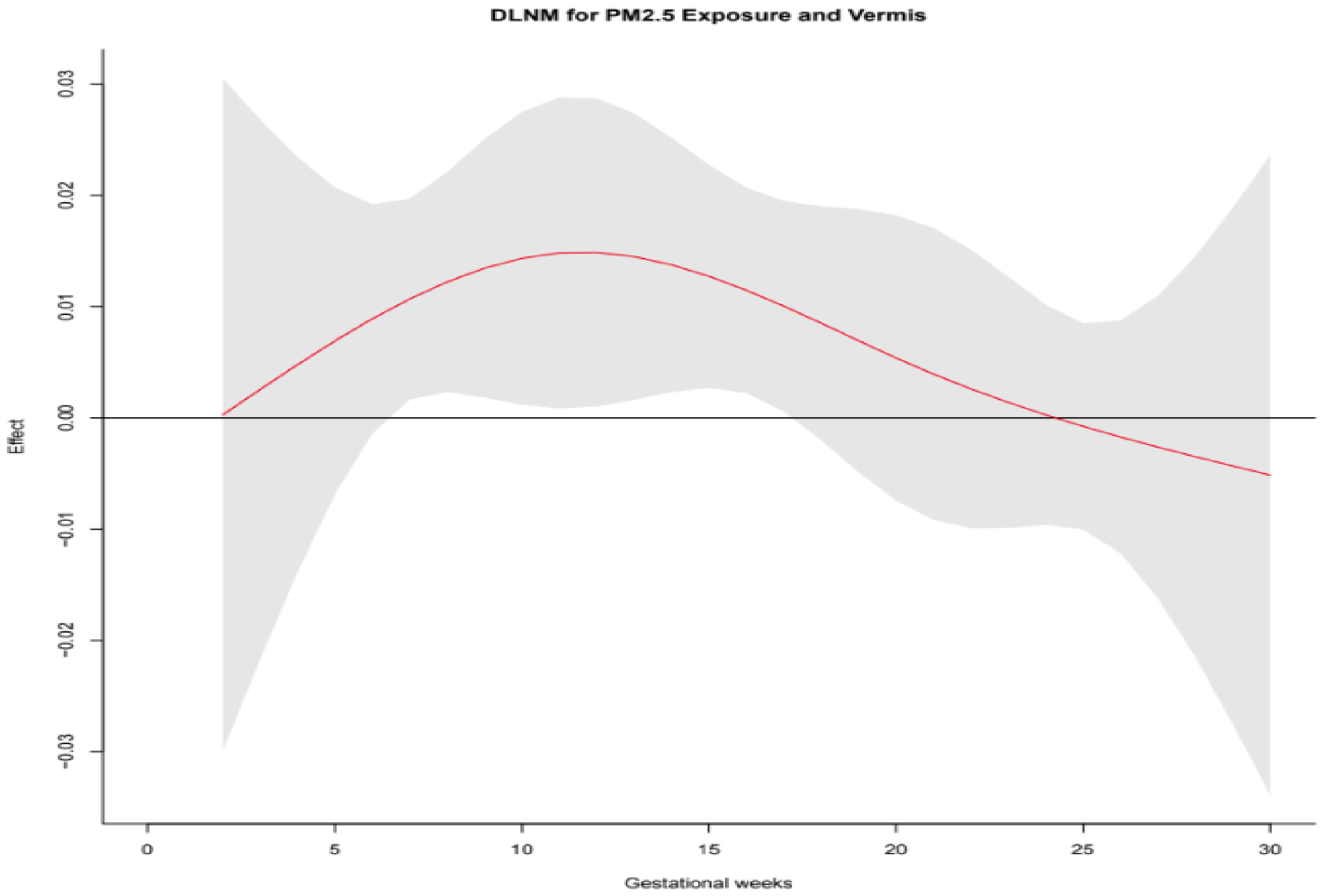
DLNM for PM2.5 Exposure and Vermis
Effect
0.03
0.02
0.01
0.00
−0.01
−0.02
−0.03
0
5
10
15
20
25
30
Gestational weeks

(C)

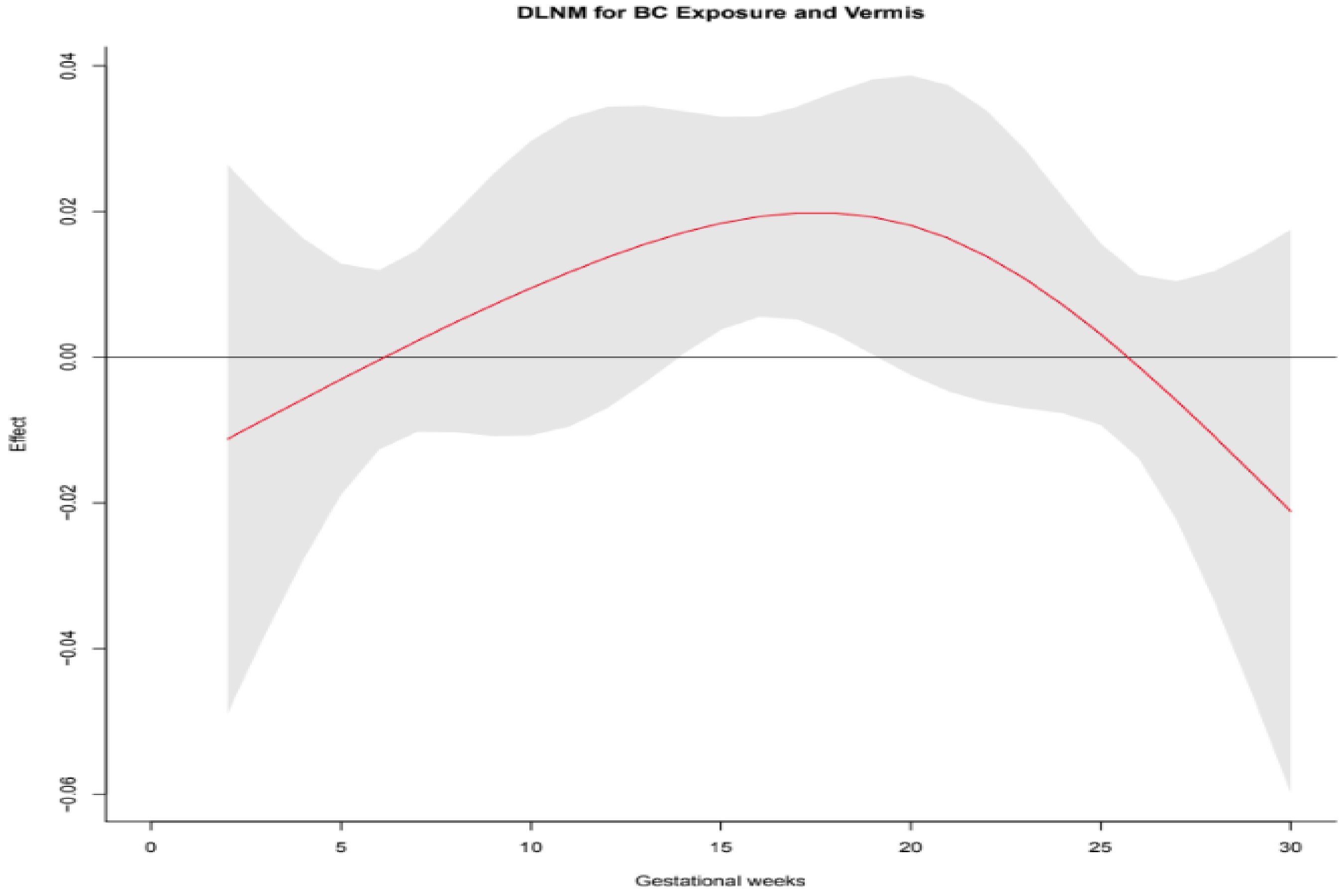

[a] Adjusted for foetal sex (boy vs girl), mother parity (multiparous vs nulliparous), maternal education (with university degree vs without university degree), ethnicity (European vs other), active smoking during pregnancy (no vs yes), passive smoking during pregnancy (no vs yes), alcohol consumption during pregnancy (no vs yes), and gestational age at ultrasound (days), and hospital and rater as random effects.

Abbreviations: NO_2, nitrogen dioxide; $PM_{2·5}$, particulate matter with an aerodynamic diameter < 2·5 µm; BC, black carbon; DLNM, Distributed Lag Non-linear Models.

Figure S13: Association of per IQR increases exposure to NO_2, $PM_{2·5}$, and BC with the transverse cerebellar diameter during each week of pregnancy (weeks 1 to 30). [a] (A: NO_2, B: $PM_{2·5}$, C: BC)

(A)

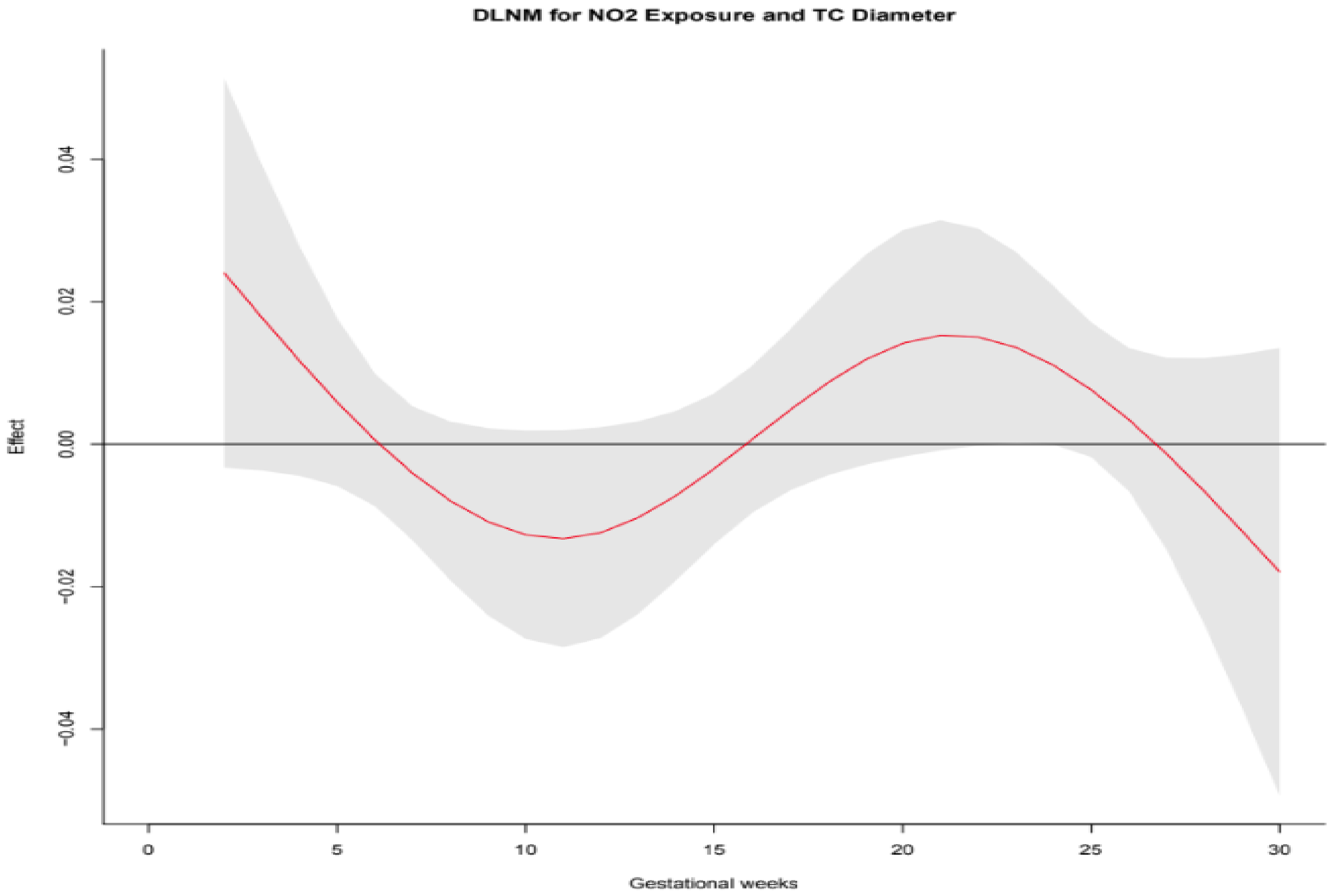

(B)

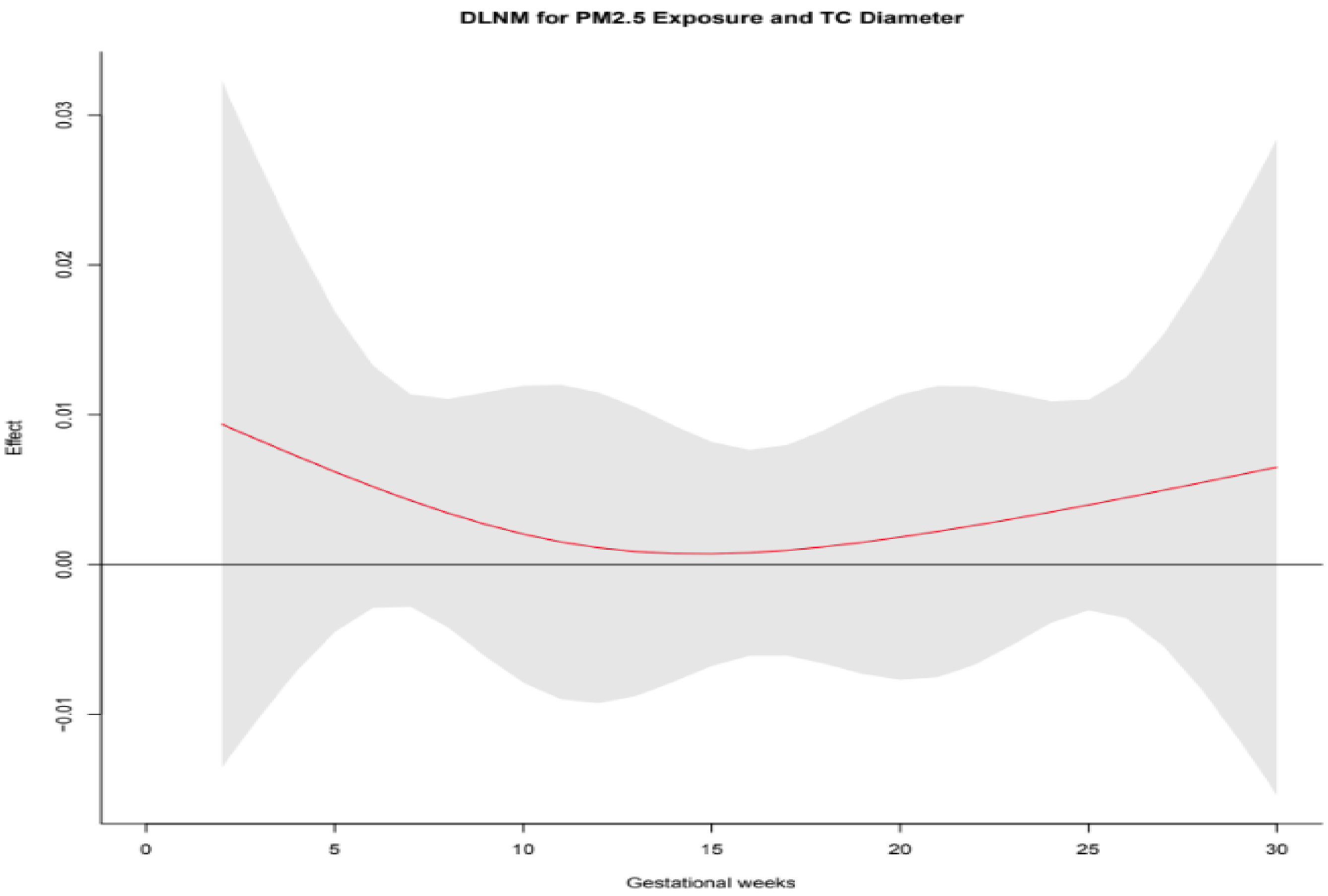
DLNM for PM2.5 Exposure and TC Diameter
Effect
0.03
0.02
0.01
0.00
-0.01
0
5
10
15
20
25
30
Gestational weeks

(C)

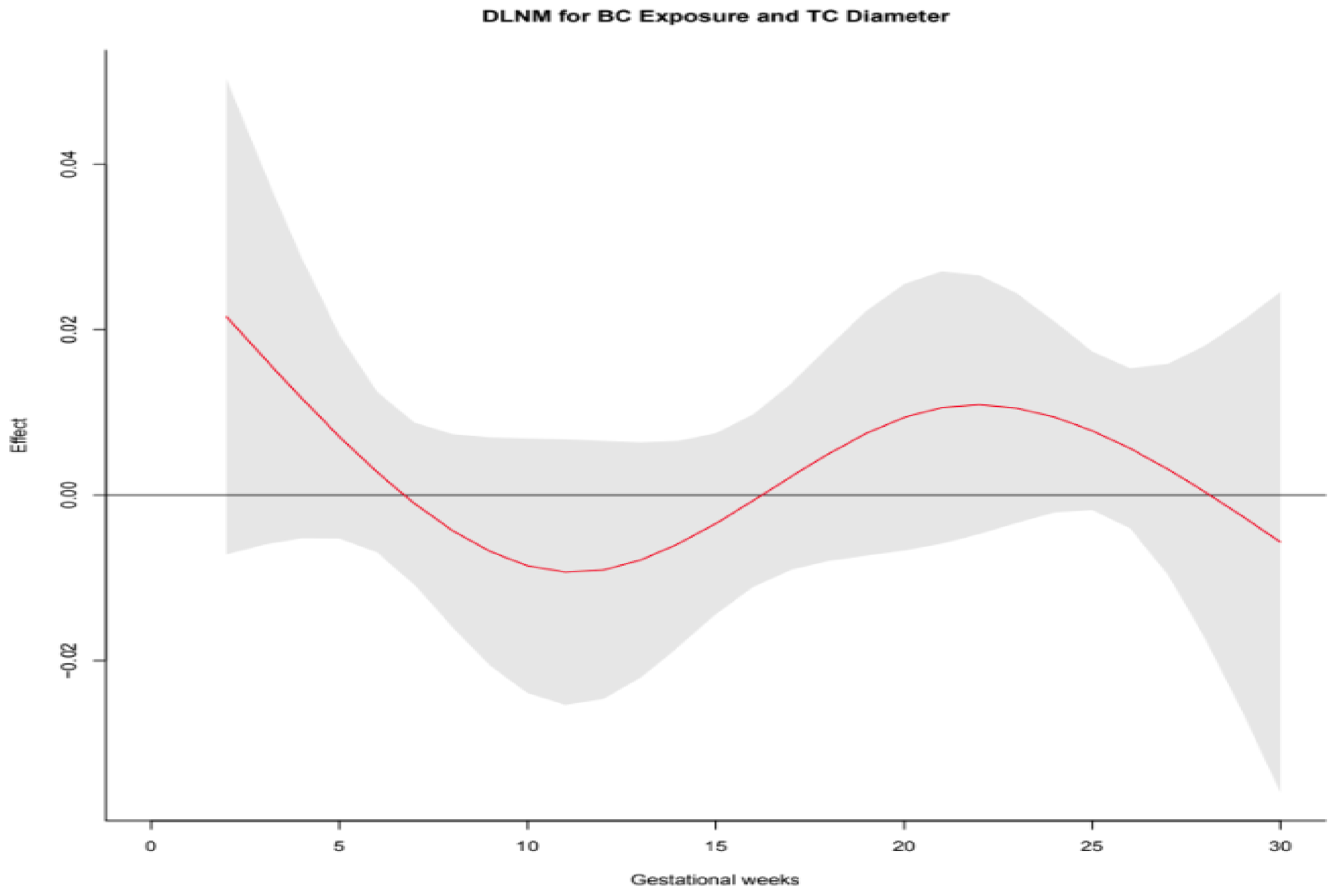

[a] Adjusted for foetal sex (boy vs girl), mother parity (multiparous vs nulliparous), maternal education (with university degree vs without university degree), ethnicity (European vs other), active smoking during pregnancy (no vs yes), passive smoking during pregnancy (no vs yes), alcohol consumption during pregnancy (no vs yes), and gestational age at ultrasound (days), and hospital and rater as random effects.

Abbreviations: NO_2, nitrogen dioxide; $PM_{2 \cdot 5}$, particulate matter with an aerodynamic diameter < 2·5 µm; BC, black carbon; DLNM, Distributed Lag Non-linear Models.

Figure S14: Association of per IQR increases exposure to NO_2, $PM_{2\cdot5}$, and BC with the percent difference in brain morphological structures (%) using all complete cases. [a]

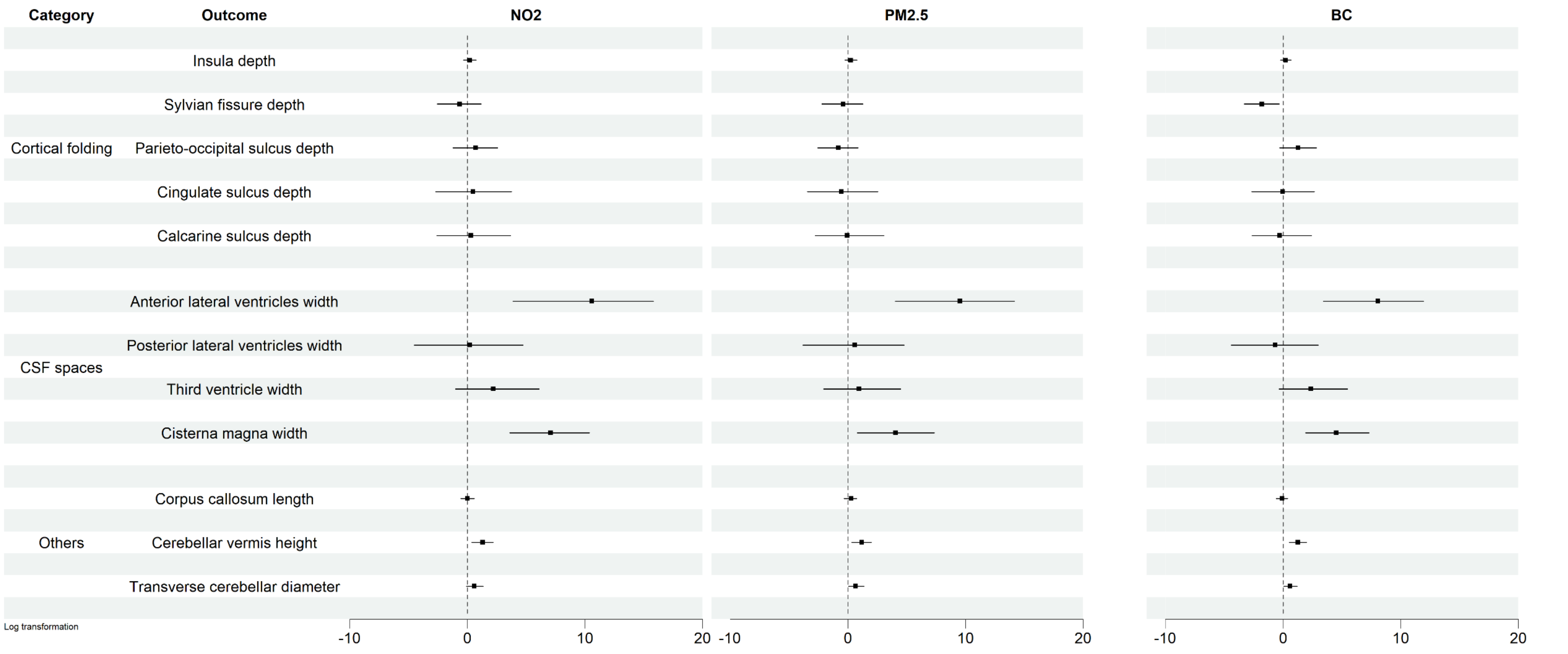

[a] Adjusted for foetal sex (boy vs girl), mother parity (multiparous vs nulliparous), maternal education (with university degree vs without university degree), ethnicity (European vs other), active smoking during pregnancy (no vs yes), passive smoking during pregnancy (no vs yes), alcohol consumption during pregnancy (no vs yes), and gestational age at ultrasound (days), and hospital and rater as random effects.

Abbreviations: N, number of samples; IQR, interquartile range; NO_2, nitrogen dioxide; $PM_{2\cdot5}$, particulate matter with an aerodynamic diameter < 2·5 µm; BC, black carbon; CSF, cerebrospinal fluid.

Table S6: Association of per IQR increases exposure to NO_2, $PM_{2·5}$, and BC with the percent difference in brain morphological structures (%) using all complete cases. [a]

Category	Outcome	N	NO_2 % (95% CI)	$PM_{2.5}$ % (95% CI)	BC % (95% CI)
Cortical folding	Insula depth	638	0·19 (-0·35, 0·75)	0·26 (-0·25, 0·79)	0·22 (-0·23, 0·68)
	Sylvian fissure depth	638	-0·65 (-2·58, 1·18)	-0·40 (-2·21, 1·29)	-1·79 (-3·31, -0·33)
	Parieto-occipital sulcus depth	520	0·69 (-1·23, 2·56)	-0·81 (-2·55, 0·88)	1·29 (-0·29, 2·84)
	Cingulate sulcus depth	536	0·47 (-2·71, 3·75)	-0·53 (-3·44, 2·55)	-0·04 (-2·67, 2·66)
	Calcarine sulcus depth	437	0·30 (-2·59, 3·66)	-0·06 (-2·77, 3·08)	-0·28 (-2·63, 2·39)
CSF spaces	Anterior lateral ventricles width	443	10·58 (3·88, 15·80)	9·53 (4·04, 14·18)	8·08 (3·44, 11·93)
	Posterior lateral ventricles width	624	0·20 (-4·53, 4·72)	0·58 (-3·81, 4·79)	-0·67 (-4·42, 2·99)
	Third ventricle width	646	2·18 (-1·04, 6·10)	0·94 (-2·06, 4·52)	2·36 (-0·35, 5·49)
	Cisterna magna width	584	7·07 (3·59, 10·38)	4·07 (0·82, 7·36)	4·51 (1·91, 7·30)
Others	Corpus callosum length	636	0·02 (-0·56, 0·58)	0·28 (-0·33, 0·75)	-0·09 (-0·57, 0·39)
	Cerebellar vermis height	610	1·30 (0·38, 2·19)	1·18 (0·34, 2·00)	1·25 (0·52, 1·98)
	Transcerebellar diameter	598	0·57 (-0·07, 1·33)	0·66 (0·07, 1·37)	0·59 (0·06, 1·19)

[a] Adjusted for foetal sex (boy vs girl), mother parity (multiparous vs nulliparous), maternal education (with university degree vs without university degree), ethnicity (European vs other), active smoking during pregnancy (no vs yes), passive smoking during pregnancy (no vs yes), alcohol consumption during pregnancy (no vs yes), and gestational age at ultrasound (days), and hospital and rater as random effects.

Abbreviations: N, number of samples; IQR, interquartile range; NO_2, nitrogen dioxide; $PM_{2·5}$, particulate matter with an aerodynamic diameter < 2·5 μm; BC, black carbon; CSF, cerebrospinal fluid.

Figure S15: Association of per IQR increases exposure to NO_2, $PM_{2\cdot5}$, and BC with the percent difference in brain morphological structures (%) further adjusting possible covariates. [a]

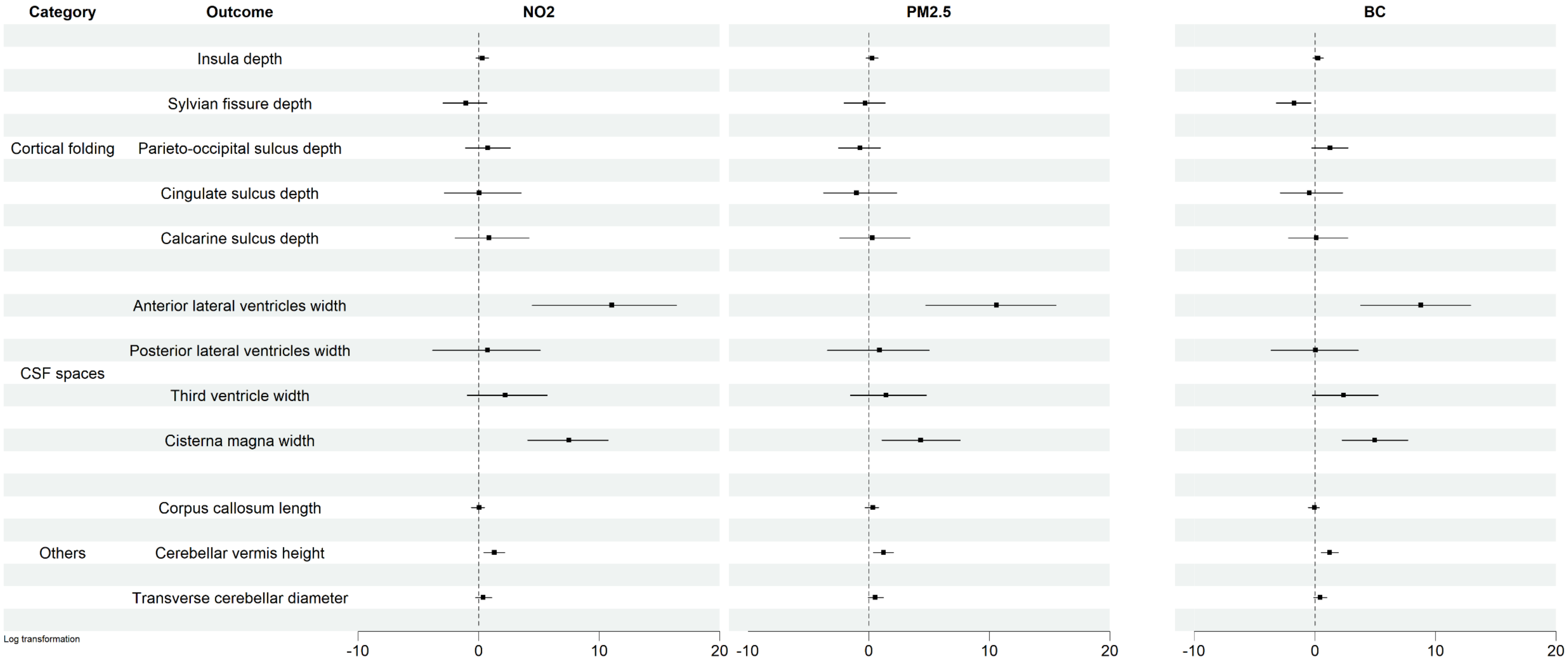

[a] Adjusted for foetal sex (boy vs girl), mother parity (multiparous vs nulliparous), maternal education (with university degree vs without university degree), ethnicity (European vs other), active smoking during pregnancy (no vs yes), passive smoking during pregnancy (no vs yes), alcohol consumption during pregnancy (no vs yes), gestational age at ultrasound (days), maternal thyroid pathology (no vs yes), estimated foetal weight, maternal body mass index, and maternal age at ultrasound, and hospital and rater as random effects.

Abbreviations: N, number of samples; IQR, interquartile range; NO_2, nitrogen dioxide; $PM_{2\cdot5}$, particulate matter with an aerodynamic diameter < 2·5 µm; BC, black carbon; CSF, cerebrospinal fluid.

Table S7: Association of per IQR increases exposure to NO_2, $PM_{2\cdot5}$, and BC with the percent difference in brain morphological structures (%) further adjusting possible covariates. [a]

Category	Outcome	N	NO_2 % (95% CI)	$PM_{2.5}$ % (95% CI)	BC % (95% CI)
**Cortical folding**	Insula depth	673	0·28 (-0·23, 0·81)	0·28 (-0·21, 0·79)	0·26 (-0·16, 0·70)
	Sylvian fissure depth	673	-1·07 (-2·98, 0·68)	-0·28 (-2·05, 1·37)	-1·73 (-3·21, -0·33)
	Parieto-occipital sulcus depth	549	0·76 (-1·11, 2·61)	-0·71 (-2·50, 0·99)	1·25 (-0·28, 2·77)
	Cingulate sulcus depth	570	0·03 (-2·86, 3·52)	-1·01 (-3·75, 2·32)	-0·47 (-2·87, 2·31)
	Calcarine sulcus depth	458	0·86 (-1·97, 4·17)	0·30 (-2·38, 3·43)	0·13 (-2·17, 2·72)
**CSF spaces**	Anterior lateral ventricles width	471	11·03 (4·44, 16·40)	10·62 (4·73, 15·53)	8·80 (3·80, 12·92)
	Posterior lateral ventricles width	657	0·74 (-3·84, 5·12)	0·91 (-3·43, 5·02)	0·06 (-3·63, 3·61)
	Third ventricle width	681	2·18 (-0·95, 5·67)	1·43 (-1·54, 4·78)	2·39 (-0·23, 5·25)
	Cisterna magna width	613	7·48 (4·06, 10·73)	4·32 (1·09, 7·60)	4·95 (2·24, 7·72)
**Others**	Corpus callosum length	672	0·03 (-0·63, 0·48)	0·35 (-0·30, 0·83)	-0·03 (-0·55, 0·38)
	Cerebellar vermis height	645	1·30 (0·42, 2·15)	1·23 (0·40, 2·03)	1·24 (0·53, 1·93)
	Transcerebellar diameter	627	0·37 (-0·26, 1·07)	0·55 (-0·03, 1·23)	0·43 (-0·08, 0·99)

[a] Adjusted for foetal sex (boy vs girl), mother parity (multiparous vs nulliparous), maternal education (with university degree vs without university degree), ethnicity (European vs other), active smoking during pregnancy (no vs yes), passive smoking during pregnancy (no vs yes), alcohol consumption during pregnancy (no vs yes), gestational age at ultrasound (days), maternal thyroid pathology (no vs yes), estimated foetal weight, maternal body mass index, and maternal age at ultrasound, and hospital and rater as random effects.

Abbreviations: N, number of samples; IQR, interquartile range; NO_2, nitrogen dioxide; $PM_{2\cdot5}$, particulate matter with an aerodynamic diameter < 2·5 μm; BC, black carbon; CSF, cerebrospinal fluid.

Figure S16: Association of per IQR increases exposure to NO_2, $PM_{2.5}$, and BC with the percent difference in brain morphological structures (%) further adjusting prenatal complications.[a] (A: intrauterine growth retardation (IUGR); B: preeclampsia; C: gestational diabetes; D: gestational hypertension; E: all prenatal complications)

(A)

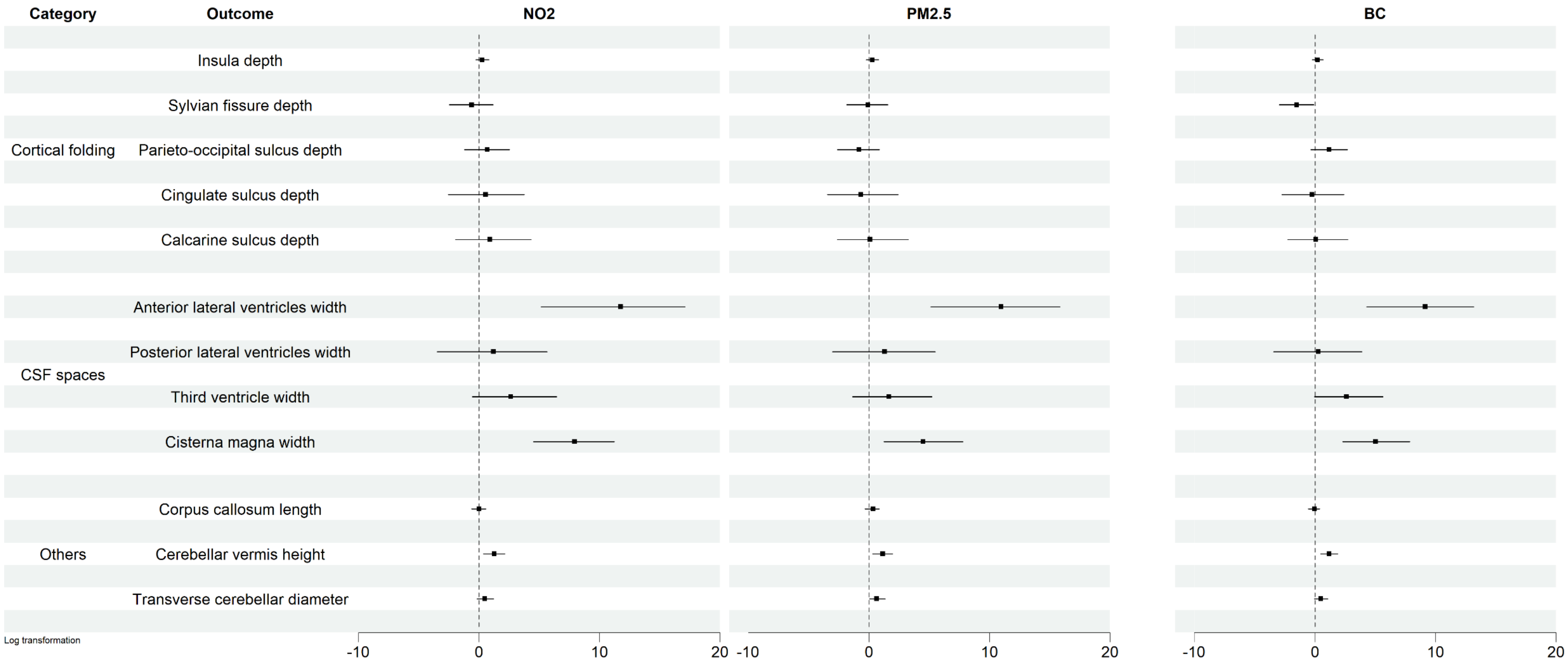

[a] Adjusted for foetal sex (boy vs girl), mother parity (multiparous vs nulliparous), maternal education (with university degree vs without university degree), ethnicity (European vs other), active smoking during pregnancy (no vs yes), passive smoking during pregnancy (no vs yes), alcohol consumption during pregnancy (no vs yes), gestational age at ultrasound (days), and intrauterine growth retardation (IUGR) (no vs yes), and hospital and rater as random effects.

(B)

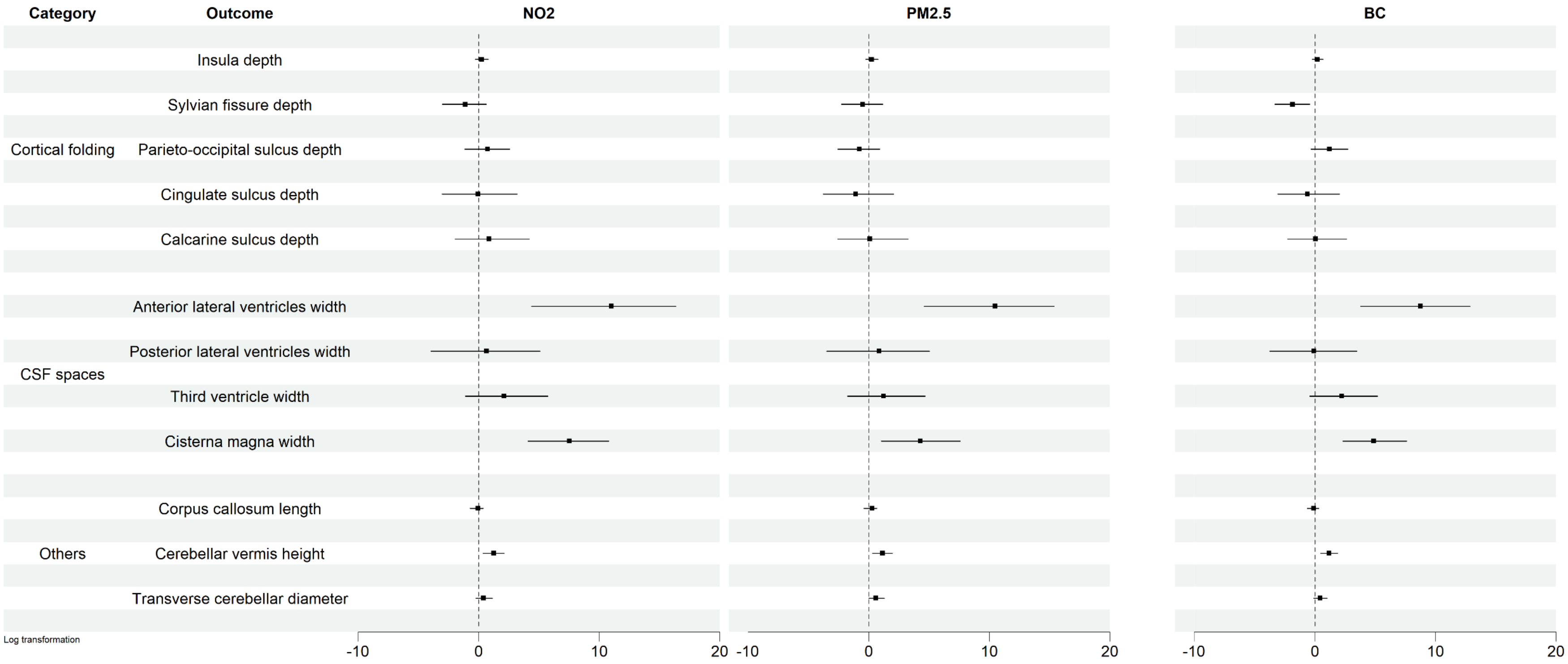

[a] Adjusted for foetal sex (boy vs girl), mother parity (multiparous vs nulliparous), maternal education (with university degree vs without university degree), ethnicity (European vs other), active smoking during pregnancy (no vs yes), passive smoking during pregnancy (no vs yes), alcohol consumption during pregnancy (no vs yes), gestational age at ultrasound (days), and preeclampsia (no vs yes), and hospital and rater as random effects.

(C)

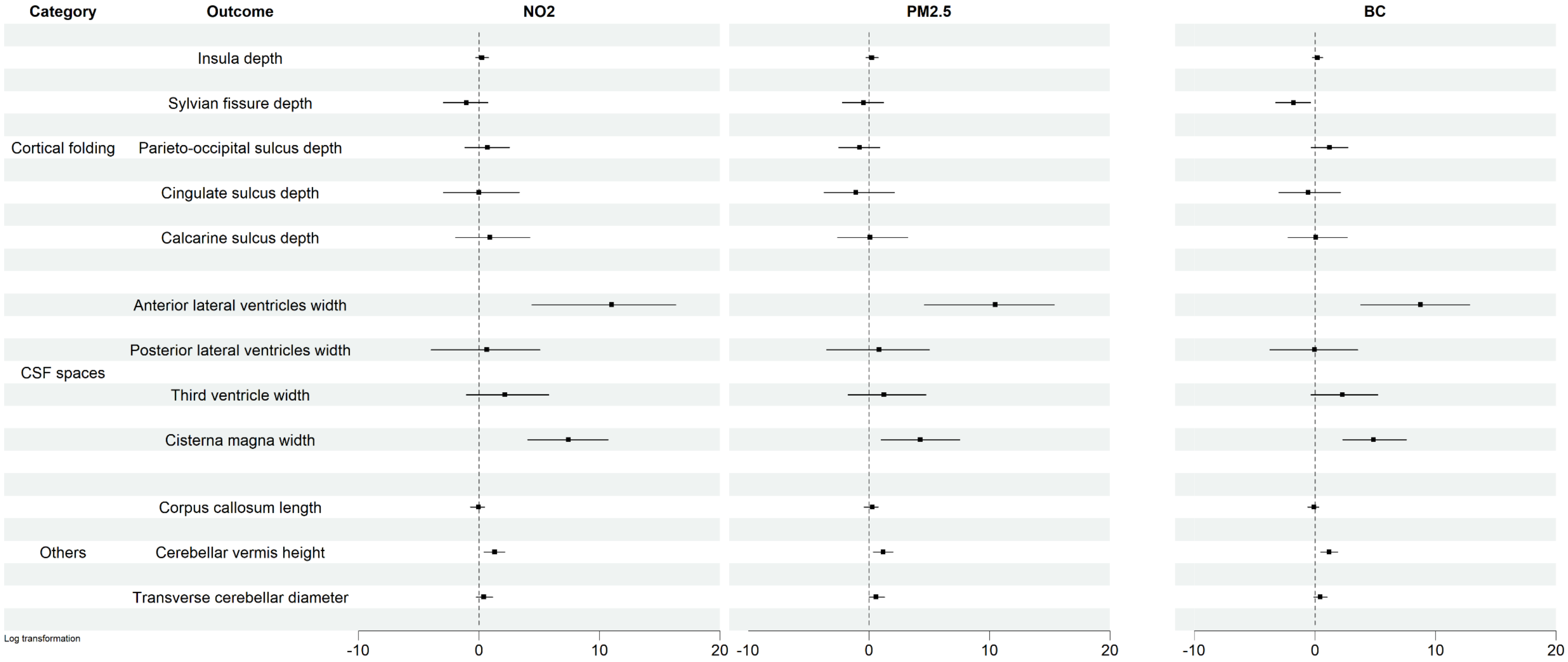

[a] Adjusted for foetal sex (boy vs girl), mother parity (multiparous vs nulliparous), maternal education (with university degree vs without university degree), ethnicity (European vs other), active smoking during pregnancy (no vs yes), passive smoking during pregnancy (no vs yes), alcohol consumption during pregnancy (no vs yes), gestational age at ultrasound (days), and gestational diabetes (no vs yes), and hospital and rater as random effects.

(D)

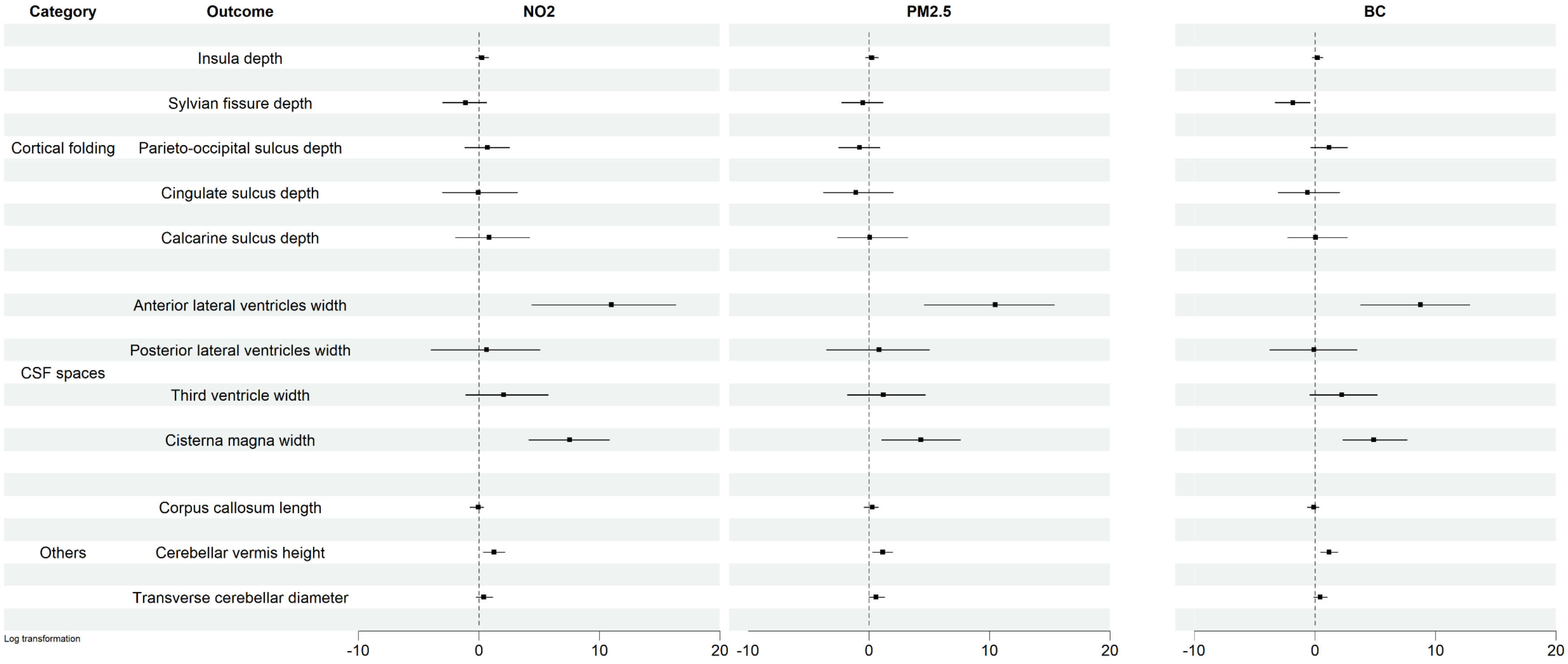

[a] Adjusted for foetal sex (boy vs girl), mother parity (multiparous vs nulliparous), maternal education (with university degree vs without university degree), ethnicity (European vs other), active smoking during pregnancy (no vs yes), passive smoking during pregnancy (no vs yes), alcohol consumption during pregnancy (no vs yes), gestational age at ultrasound (days), and gestational hypertension (no vs yes), and hospital and rater as random effects.

(E)

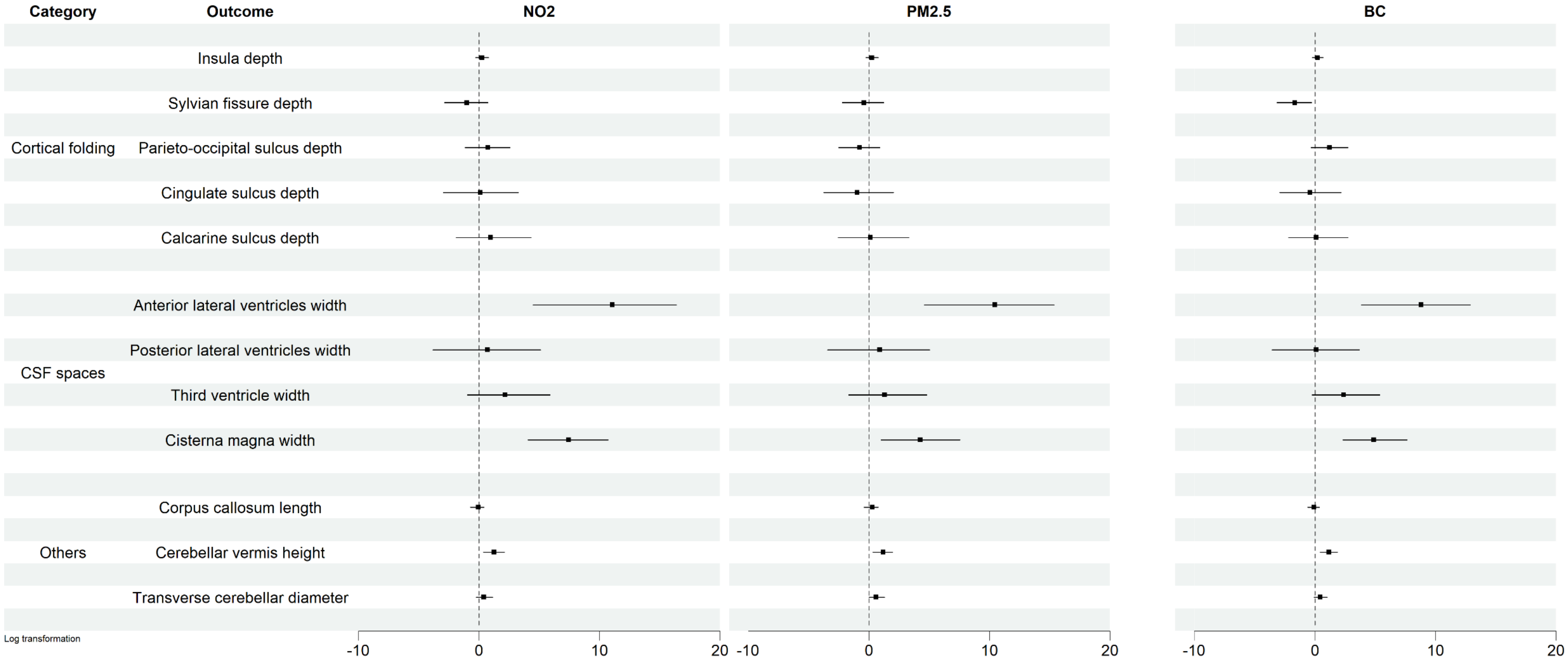

[a] Adjusted for foetal sex (boy vs girl), mother parity (multiparous vs nulliparous), maternal education (with university degree vs without university degree), ethnicity (European vs other), active smoking during pregnancy (no vs yes), passive smoking during pregnancy (no vs yes), alcohol consumption during pregnancy (no vs yes), gestational age at ultrasound (days), intrauterine growth retardation (IUGR) (no vs yes), preeclampsia (no vs yes), gestational diabetes (no vs yes), and gestational hypertension (no vs yes), and hospital and rater as random effects.

Abbreviations: N, number of samples; IQR, interquartile range; NO_2, nitrogen dioxide; $PM_{2·5}$, particulate matter with an aerodynamic diameter < 2·5 µm; BC, black carbon; CSF, cerebrospinal fluid.

Table S8: Association of per IQR increases exposure to NO_2, $PM_{2 \cdot 5}$, and BC with the percent difference in brain morphological structures (%) further adjusting prenatal complications. [a] (A: intrauterine growth retardation (IUGR); B: preeclampsia; C: gestational diabetes; D: gestational hypertension; E: all prenatal complications)

(A)

Category	Outcome	N	NO_2 % (95% CI)	$PM_{2.5}$ % (95% CI)	BC % (95% CI)
**Cortical folding**	Insula depth	673	0·26 (-0·27, 0·82)	0·27 (-0·23, 0·81)	0·23 (-0·21, 0·69)
	Sylvian fissure depth	673	-0·61 (-2·46, 1·17)	-0·08 (-1·85, 1·59)	-1·51 (-2·96, -0·08)
	Parieto-occipital sulcus depth	549	0·68 (-1·22, 2·54)	-0·82 (-2·60, 0·89)	1·19 (-0·36, 2·71)
	Cingulate sulcus depth	570	0·55 (-2·54, 3·75)	-0·67 (-3·43, 2·43)	-0·24 (-2·73, 2·40)
	Calcarine sulcus depth	458	0·92 (-1·94, 4·32)	0·09 (-2·60, 3·28)	0·08 (-2·24, 2·71)
**CSF spaces**	Anterior lateral ventricles width	471	11·75 (5·15, 17·08)	10·98 (5·14, 15·85)	9·15 (4·30, 13·18)
	Posterior lateral ventricles width	657	1·18 (-3·48, 5·65)	1·32 (-3·01, 5·51)	0·29 (-3·43, 3·90)
	Third ventricle width	681	2·64 (-0·56, 6·44)	1·66 (-1·34, 5·23)	2·63 (-0·03, 5·65)
	Cisterna magna width	613	7·92 (4·51, 11·22)	4·49 (1·24, 7·80)	5·04 (2·29, 7·87)
**Others**	Corpus callosum length	672	0·02 (-0·63, 0·55)	0·35 (-0·32, 0·85)	-0·04 (-0·55, 0·40)
	Cerebellar vermis height	645	1·26 (0·37, 2·12)	1·16 (0·33, 1·97)	1·19 (0·47, 1·89)
	Transcerebellar diameter	627	0·46 (-0·16, 1·21)	0·65 (0·07, 1·35)	0·49 (-0·03, 1·07)

[a] Adjusted for foetal sex (boy vs girl), mother parity (multiparous vs nulliparous), maternal education (with university degree vs without university degree), ethnicity (European vs other), active smoking during pregnancy (no vs yes), passive smoking during pregnancy (no vs yes), alcohol consumption during pregnancy (no vs yes), gestational age at ultrasound (days), and intrauterine growth retardation (IUGR) (no vs yes), and hospital and rater as random effects.

(B)

Category	Outcome	N	NO_2 % (95% CI)	$PM_{2.5}$ % (95% CI)	BC % (95% CI)
**Cortical folding**	Insula depth	673	0·23 (-0·29, 0·79)	0·25 (-0·25, 0·78)	0·21 (-0·22, 0·67)
	Sylvian fissure depth	673	-1·13 (-3·05, 0·65)	-0·48 (-2·27, 1·18)	-1·84 (-3·34, -0·43)
	Parieto-occipital sulcus depth	549	0·72 (-1·16, 2·57)	-0·77 (-2·55, 0·93)	1·21 (-0·33, 2·73)
	Cingulate sulcus depth	570	-0·06 (-3·03, 3·19)	-1·08 (-3·78, 2·06)	-0·63 (-3·07, 2·03)
	Calcarine sulcus depth	458	0·86 (-1·96, 4·19)	0·11 (-2·56, 3·24)	0·04 (-2·25, 2·64)
**CSF spaces**	Anterior lateral ventricles width	471	11·01 (4·40, 16·34)	10·49 (4·60, 15·37)	8·77 (3·79, 12·85)
	Posterior lateral ventricles width	657	0·65 (-3·97, 5·08)	0·87 (-3·49, 5·04)	-0·07 (-3·73, 3·49)
	Third ventricle width	681	2·08 (-1·10, 5·76)	1·23 (-1·77, 4·69)	2·24 (-0·42, 5·21)
	Cisterna magna width	613	7·51 (4·08, 10·80)	4·30 (1·05, 7·59)	4·88 (2·32, 7·63)
**Others**	Corpus callosum length	672	-0·08 (-0·73, 0·39)	0·27 (-0·40, 0·67)	-0·10 (-0·62, 0·32)
	Cerebellar vermis height	645	1·25 (0·36, 2·11)	1·16 (0·33, 1·97)	1·18 (0·47, 1·88)
	Transcerebellar diameter	627	0·39 (-0·23, 1·12)	0·59 (0·00, 1·29)	0·44 (-0·08, 1·02)

[a] Adjusted for foetal sex (boy vs girl), mother parity (multiparous vs nulliparous), maternal education (with university degree vs without university degree), ethnicity (European vs other), active smoking during pregnancy (no vs yes), passive smoking during pregnancy (no vs yes), alcohol consumption during pregnancy (no vs yes), gestational age at ultrasound (days), and preeclampsia (no vs yes), and hospital and rater as random effects.

(C)

Category	Outcome	N	NO_2 % (95% CI)	$PM_{2.5}$ % (95% CI)	BC % (95% CI)
**Cortical folding**	Insula depth	673	0·23 (-0·30, 0·78)	0·25 (-0·25, 0·77)	0·21 (-0·22, 0·66)
	Sylvian fissure depth	673	-1·05 (-2·99, 0·73)	-0·45 (-2·21, 1·23)	-1·78 (-3·28, -0·36)
	Parieto-occipital sulcus depth	549	0·70 (-1·18, 2·55)	-0·78 (-2·52, 0·90)	1·21 (-0·33, 2·73)
	Cingulate sulcus depth	570	-0·01 (-2·95, 3·33)	-1·09 (-3·72, 2·13)	-0·57 (-2·99, 2·13)
	Calcarine sulcus depth	458	0·90 (-1·94, 4·22)	0·09 (-2·59, 3·23)	0·07 (-2·23, 2·68)
**CSF spaces**	Anterior lateral ventricles width	471	11·00 (4·39, 16·32)	10·49 (4·60, 15·39)	8·76 (3·78, 12·84)
	Posterior lateral ventricles width	657	0·64 (-3·98, 5·07)	0·85 (-3·52, 5·01)	-0·04 (-3·73, 3·55)
	Third ventricle width	681	2·13 (-1·05, 5·81)	1·27 (-1·73, 4·73)	2·29 (-0·36, 5·23)
	Cisterna magna width	613	7·42 (4·04, 10·70)	4·26 (1·02, 7·55)	4·85 (2·29, 7·59)
**Others**	Corpus callosum length	672	-0·05 (-0·72, 0·45)	0·28 (-0·39, 0·79)	-0·08 (-0·61, 0·33)
	Cerebellar vermis height	645	1·28 (0·40, 2·14)	1·19 (0·36, 2·00)	1·19 (0·48, 1·89)
	Transcerebellar diameter	627	0·40 (-0·23, 1·12)	0·59 (0·00, 1·29)	0·43 (-0·08, 1·01)

[a] Adjusted for foetal sex (boy vs girl), mother parity (multiparous vs nulliparous), maternal education (with university degree vs without university degree), ethnicity (European vs other), active smoking during pregnancy (no vs yes), passive smoking during pregnancy (no vs yes), alcohol consumption during pregnancy (no vs yes), gestational age at ultrasound (days), and gestational diabetes (no vs yes), and hospital and rater as random effects.

(D)

Category	Outcome	N	NO_2<br>% (95% CI)	$PM_{2.5}$<br>% (95% CI)	BC<br>% (95% CI)
**Cortical folding**	Insula depth	673	0·23 (-0·30, 0·78)	0·25 (-0·26, 0·77)	0·21 (-0·23, 0·66)
	Sylvian fissure depth	673	-1·12 (-3·04, 0·65)	-0·49 (-2·27, 1·18)	-1·83 (-3·30, -0·41)
	Parieto-occipital sulcus depth	549	0·69 (-1·19, 2·54)	-0·78 (-2·52, 0·91)	1·19 (-0·35, 2·71)
	Cingulate sulcus depth	570	-0·06 (-3·05, 3·18)	-1·09 (-3·78, 2·02)	-0·61 (-3·06, 2·03)
	Calcarine sulcus depth	458	0·84 (-1·97, 4·18)	0·07 (-2·58, 3·22)	0·04 (-2·24, 2·65)
**CSF spaces**	Anterior lateral ventricles width	471	10·99 (4·39, 16·31)	10·49 (4·61, 15·37)	8·75 (3·78, 12·83)
	Posterior lateral ventricles width	657	0·63 (-3·99, 5·06)	0·85 (-3·51, 5·02)	-0·07 (-3·73, 3·49)
	Third ventricle width	681	2·05 (-1·12, 5·74)	1·20 (-1·80, 4·69)	2·23 (-0·42, 5·17)
	Cisterna magna width	613	7·52 (4·13, 10·81)	4·31 (1·06, 7·60)	4·89 (2·33, 7·64)
**Others**	Corpus callosum length	672	-0·08 (-0·74, 0·39)	0·27 (-0·41, 0·77)	-0·10 (-0·63, 0·32)
	Cerebellar vermis height	645	1·25 (0·37, 2·12)	1·16 (0·33, 1·97)	1·18 (0·47, 1·88)
	Transcerebellar diameter	627	0·39 (-0·23, 1·12)	0·59 (0·00, 1·29)	0·44 (-0·08, 1·01)

[a] Adjusted for foetal sex (boy vs girl), mother parity (multiparous vs nulliparous), maternal education (with university degree vs without university degree), ethnicity (European vs other), active smoking during pregnancy (no vs yes), passive smoking during pregnancy (no vs yes), alcohol consumption during pregnancy (no vs yes), gestational age at ultrasound (days), and gestational hypertension (no vs yes), and hospital and rater as random effects.

(E)

Category	Outcome	N	NO_2 % (95% CI)	$PM_{2.5}$ % (95% CI)	BC % (95% CI)
**Cortical folding**	Insula depth	673	0·23 (-0·29, 0·78)	0·25 (-0·25, 0·78)	0·22 (-0·22, 0·67)
	Sylvian fissure depth	673	-1·03 (-2·88, 0·73)	-0·42 (-2·19, 1·24)	-1·68 (-3·14, -0·26)
	Parieto-occipital sulcus depth	549	0·72 (-1·16, 2·57)	-0·77 (-2·51, 0·92)	1·21 (-0·33, 2·73)
	Cingulate sulcus depth	570	0·11 (-2·96, 3·27)	-0·99 (-3·75, 2·05)	-0·42 (-2·93, 2·16)
	Calcarine sulcus depth	458	0·95 (-1·88, 4·31)	0·13 (-2·54, 3·29)	0·11 (-2·18, 2·73)
**CSF spaces**	Anterior lateral ventricles width	471	11·05 (4·48, 16·36)	10·47 (4·62, 15·35)	8·82 (3·87, 12·89)
	Posterior lateral ventricles width	657	0·71 (-3·83, 5·10)	0·90 (-3·44, 5·06)	0·11 (-3·55, 3·68)
	Third ventricle width	681	2·17 (-0·99, 5·90)	1·30 (-1·69, 4·81)	2·40 (-0·25, 5·38)
	Cisterna magna width	613	7·44 (4·05, 10·70)	4·27 (1·02, 7·56)	4·88 (2·33, 7·64)
**Others**	Corpus callosum length	672	-0·06 (-0·73, 0·40)	0·27 (-0·41, 0·79)	-0·07 (-0·60, 0·36)
	Cerebellar vermis height	645	1·25 (0·37, 2·11)	1·17 (0·34, 1·97)	1·16 (0·45, 1·87)
	Transcerebellar diameter	627	0·39 (-0·24, 1·12)	0·58 (0·00, 1·29)	0·44 (-0·07, 1·02)

[a] Adjusted for foetal sex (boy vs girl), mother parity (multiparous vs nulliparous), maternal education (with university degree vs without university degree), ethnicity (European vs other), active smoking during pregnancy (no vs yes), passive smoking during pregnancy (no vs yes), alcohol consumption during pregnancy (no vs yes), gestational age at ultrasound (days), intrauterine growth retardation (IUGR) (no vs yes), preeclampsia (no vs yes), gestational diabetes (no vs yes), and gestational hypertension (no vs yes), and hospital and rater as random effects.

Abbreviations: N, number of samples; IQR, interquartile range; NO_2, nitrogen dioxide; $PM_{2·5}$, particulate matter with an aerodynamic diameter < 2·5 µm; BC, black carbon; CSF, cerebrospinal fluid.

Figure S17: Association of per IQR increases exposure to NO_2, $PM_{2·5}$, and BC with the percent difference in brain morphological structures (%) after removing the participants diagnosed with one of the prenatal complications (i.e., intrauterine growth retardation (IUGR), preeclampsia, gestational diabetes, and gestational hypertension).[a]

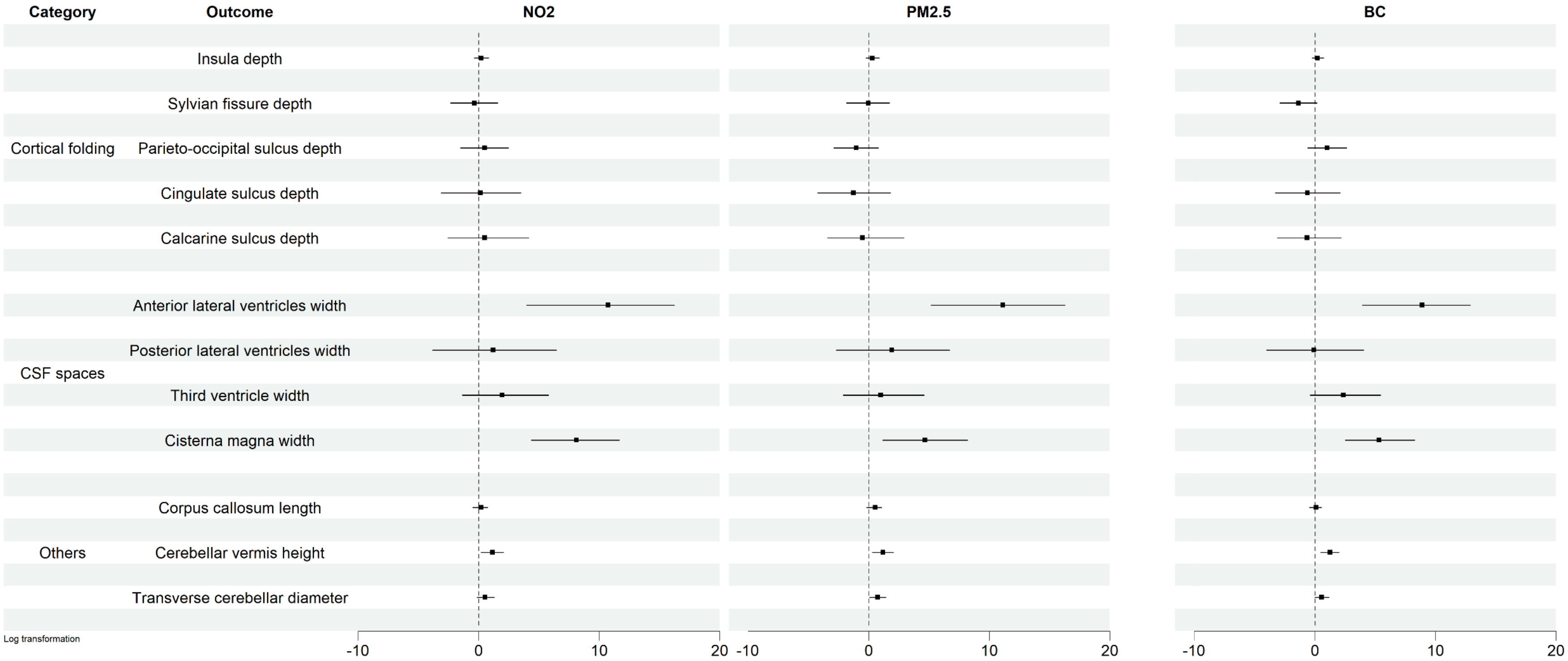

[a] Adjusted for foetal sex (boy vs girl), mother parity (multiparous vs nulliparous), maternal education (with university degree vs without university degree), ethnicity (European vs other), active smoking during pregnancy (no vs yes), passive smoking during pregnancy (no vs yes), alcohol consumption during pregnancy (no vs yes), and gestational age at ultrasound (days), and hospital and rater as random effects.

Abbreviations: N, number of samples; IQR, interquartile range; NO_2, nitrogen dioxide; $PM_{2·5}$, particulate matter with an aerodynamic diameter < 2·5 µm; BC, black carbon; CSF, cerebrospinal fluid.

Table S9: Association of per IQR increases exposure to NO_2, $PM_{2·5}$, and BC with the percent difference in brain morphological structures (%) after removing the participants diagnosed with one of the prenatal complications (i.e., intrauterine growth retardation (IUGR), preeclampsia, gestational diabetes, and gestational hypertension)

Category	Outcome	N	NO_2 % (95% CI)	$PM_{2.5}$ % (95% CI)	BC % (95% CI)
Cortical folding	Insula depth	595	0·21 (-0·36, 0·81)	0·31 (-0·23, 0·88)	0·24 (-0·23, 0·73)
	Sylvian fissure depth	595	-0·36 (-2·34, 1·58)	-0·03 (-1·84, 1·74)	-1·37 (-2·93, 0·20)
	Parieto-occipital sulcus depth	490	0·49 (-1·52, 2·45)	-1·02 (-2·90, 0·80)	1·02 (-0·61, 2·62)
	Cingulate sulcus depth	507	0·13 (-3·12, 3·48)	-1·27 (-4·24, 1·80)	-0·63 (-3·28, 2·09)
	Calcarine sulcus depth	406	0·50 (-2·56, 4·14)	-0·51 (-3·39, 2·91)	-0·64 (-3·09, 2·16)
CSF spaces	Anterior lateral ventricles width	418	10·72 (3·98, 16·21)	11·12 (5·18, 16·27)	8·89 (3·94, 12·90)
	Posterior lateral ventricles width	585	1·19 (-3·83, 6·45)	1·93 (-2·70, 6·72)	-0·09 (-4·00, 4·05)
	Third ventricle width	603	1·93 (-1·36, 5·78)	0·99 (-2·12, 4·62)	2·37 (-0·39, 5·47)
	Cisterna magna width	545	8·10 (4·37, 11·67)	4·67 (1·17, 8·21)	5·32 (2·52, 8·29)
Others	Corpus callosum length	599	0·20 (-0·50, 0·75)	0·53 (-0·16, 1·07)	0·11 (-0·44, 0·56)
	Cerebellar vermis height	578	1·14 (0·21, 2·05)	1·19 (0·31, 2·05)	1·25 (0·49, 1·99)
	Transcerebellar diameter	557	0·52 (-0·13, 1·28)	0·74 (0·10, 1·43)	0·56 (0·01, 1·17)

[a] Adjusted for foetal sex (boy vs girl), mother parity (multiparous vs nulliparous), maternal education (with university degree vs without university degree), ethnicity (European vs other), active smoking during pregnancy (no vs yes), passive smoking during pregnancy (no vs yes), alcohol consumption during pregnancy (no vs yes), and gestational age at ultrasound (days), and hospital and rater as random effects.

Abbreviations: N, number of samples; IQR, interquartile range; NO_2, nitrogen dioxide; $PM_{2·5}$, particulate matter with an aerodynamic diameter < 2·5 µm; BC, black carbon; CSF, cerebrospinal fluid.

Figure S18: Association of per IQR increases exposure to NO_2, $PM_{2·5}$, and BC with the percent difference in brain morphological structures (%) further adjusted for the ambient temperature.[a]

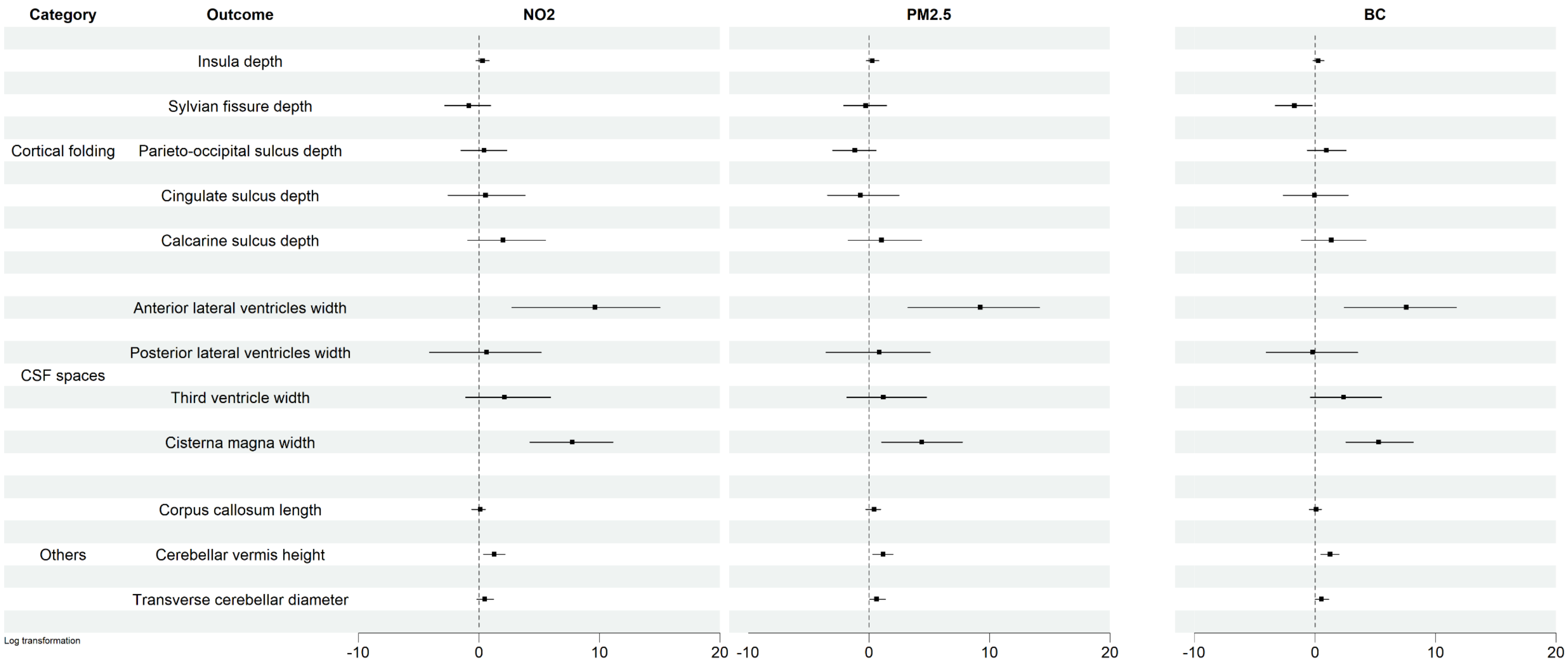

[a] Adjusted for foetal sex (boy vs girl), mother parity (multiparous vs nulliparous), maternal education (with university degree vs without university degree), ethnicity (European vs other), active smoking during pregnancy (no vs yes), passive smoking during pregnancy (no vs yes), alcohol consumption during pregnancy (no vs yes), and gestational age at ultrasound (days), and hospital and rater as random effects.

Abbreviations: N, number of samples; IQR, interquartile range; NO_2, nitrogen dioxide; $PM_{2·5}$, particulate matter with an aerodynamic diameter < 2·5 µm; BC, black carbon; CSF, cerebrospinal fluid.

Table S10: Association of per IQR increases exposure to NO_2, $PM_{2·5}$, and BC with the percent difference in brain morphological structures (%) further adjusted for the ambient temperature.[a]

Category	Outcome	N	NO_2 % (95% CI)	$PM_{2.5}$ % (95% CI)	BC % (95% CI)
**Cortical folding**	Insula depth	673	0·28 (-0·26, 0·85)	0·29 (-0·22, 0·83)	0·28 (-0·18, 0·76)
	Sylvian fissure depth	673	-0·85 (-2·85, 0·98)	-0·25 (-2·09, 1·47)	-1·71 (-3·30, -0·21)
	Parieto-occipital sulcus depth	549	0·43 (-1·52, 2·32)	-1·15 (-3·03, 0·59)	0·96 (-0·67, 2·58)
	Cingulate sulcus depth	570	0·54 (-2·58, 3·83)	-0·69 (-3·44, 2·51)	-0·02 (-2·63, 2·77)
	Calcarine sulcus depth	458	1·98 (-0·94, 5·50)	1·05 (-1·70, 4·36)	1·35 (-1·11, 4·22)
**CSF spaces**	Anterior lateral ventricles width	471	9·61 (2·71, 15·00)	9·25 (3·24, 14·15)	7·59 (2·42, 11·74)
	Posterior lateral ventricles width	657	0·63 (-4·13, 5·15)	0·86 (-3·56, 5·10)	-0·18 (-4·04, 3·55)
	Third ventricle width	681	2·11 (-1·14, 5·94)	1·21 (-1·85, 4·80)	2·38 (-0·41, 5·54)
	Cisterna magna width	613	7·75 (4·22, 11·13)	4·40 (1·05, 7·77)	5·29 (2·55, 8·17)
**Others**	Corpus callosum length	672	0·11 (-0·61, 0·53)	0·44 (-0·26, 0·98)	0·10 (-0·49, 0·54)
	Cerebellar vermis height	645	1·27 (0·37, 2·15)	1·18 (0·33, 2·00)	1·25 (0·51, 1·98)
	Transcerebellar diameter	627	0·46 (-0·18, 1·21)	0·65 (0·06, 1·38)	0·53 (-0·00, 1·15)

[a] Adjusted for foetal sex (boy vs girl), mother parity (multiparous vs nulliparous), maternal education (with university degree vs without university degree), ethnicity (European vs other), active smoking during pregnancy (no vs yes), passive smoking during pregnancy (no vs yes), alcohol consumption during pregnancy (no vs yes), and gestational age at ultrasound (days), and hospital and rater as random effects.

Abbreviations: N, number of samples; IQR, interquartile range; NO_2, nitrogen dioxide; $PM_{2·5}$, particulate matter with an aerodynamic diameter < 2·5 μm; BC, black carbon; CSF, cerebrospinal fluid.

Figure S19: Association of per IQR increases exposure to NO_2, $PM_{2·5}$, and BC with the percent difference in brain morphological structures (%) corrected by biparietal diameter. [a]

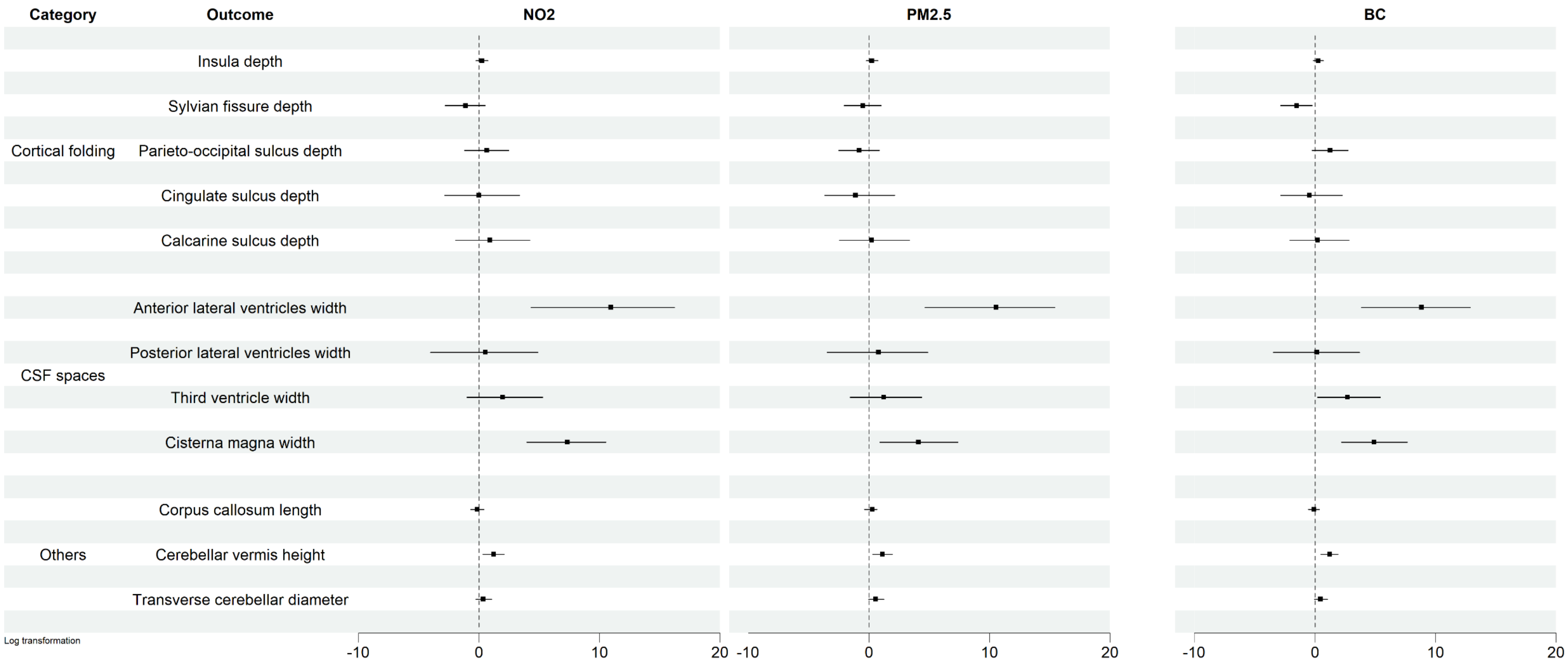

[a] Adjusted for foetal sex (boy vs girl), mother parity (multiparous vs nulliparous), maternal education (with university degree vs without university degree), ethnicity (European vs other), active smoking during pregnancy (no vs yes), passive smoking during pregnancy (no vs yes), alcohol consumption during pregnancy (no vs yes), and gestational age at ultrasound (days), and hospital and rater as random effects.

Abbreviations: N, number of samples; IQR, interquartile range; NO_2, nitrogen dioxide; $PM_{2·5}$, particulate matter with an aerodynamic diameter < 2·5 µm; BC, black carbon; CSF, cerebrospinal fluid.

Table S11: Association of per IQR increases exposure to NO_2, $PM_{2·5}$, and BC with the percent difference in brain morphological structures (%) corrected by biparietal diameter. [a]

Category	Outcome	N	NO_2 % (95% CI)	$PM_{2.5}$ % (95% CI)	BC % (95% CI)
Cortical folding	Insula depth	673	0·23 (-0·27, 0·75)	0·25 (-0·23, 0·75)	0·28 (-0·14, 0·71)
	Sylvian fissure depth	673	-1·12 (-2·84, 0·50)	-0·48 (-2·08, 1·05)	-1·52 (-2·87, -0·23)
	Parieto-occipital sulcus depth	549	0·65 (-1·21, 2·47)	-0·79 (-2·52, 0·88)	1·27 (-0·25, 2·77)
	Cingulate sulcus depth	570	-0·03 (-2·87, 3·35)	-1·12 (-3·66, 2·14)	-0·46 (-2·84, 2·26)
	Calcarine sulcus depth	458	0·90 (-1·92, 4·20)	0·24 (-2·42, 3·36)	0·22 (-2·07, 2·81)
CSF spaces	Anterior lateral ventricles width	471	10·92 (4·30, 16·22)	10·55 (4·67, 15·42)	8·83 (3·87, 12·90)
	Posterior lateral ventricles width	657	0·52 (-4·05, 4·88)	0·80 (-3·45, 4·89)	0·16 (-3·47, 3·72)
	Third ventricle width	681	1·97 (-1·01, 5·30)	1·24 (-1·56, 4·38)	2·70 (0·21, 5·43)
	Cisterna magna width	613	7·32 (3·96, 10·54)	4·12 (0·91, 7·39)	4·90 (2·20, 7·68)
Others	Corpus callosum length	672	-0·15 (-0·71, 0·40)	0·27 (-0·37, 0·68)	-0·07 (-0·56, 0·36)
	Cerebellar vermis height	645	1·22 (0·34, 2·08)	1·14 (0·32, 1·94)	1·22 (0·51, 1·91)
	Transcerebellar diameter	627	0·35 (-0·27, 1·05)	0·57 (-0·01, 1·24)	0·47 (-0·03, 1·03)

[a] Adjusted for foetal sex (boy vs girl), mother parity (multiparous vs nulliparous), maternal education (with university degree vs without university degree), ethnicity (European vs other), active smoking during pregnancy (no vs yes), passive smoking during pregnancy (no vs yes), alcohol consumption during pregnancy (no vs yes), and gestational age at ultrasound (days), and hospital and rater as random effects.

Abbreviations: N, number of samples; IQR, interquartile range; NO_2, nitrogen dioxide; $PM_{2·5}$, particulate matter with an aerodynamic diameter < 2·5 µm; BC, black carbon; CSF, cerebrospinal fluid.

Figure S20: Association of per IQR increases exposure to NO2, PM2·5, and BC in each microenvironment with the percent difference in brain morphological structures (%). a (A: NO2; B: PM2·5; C: BC)

(A) NO_2

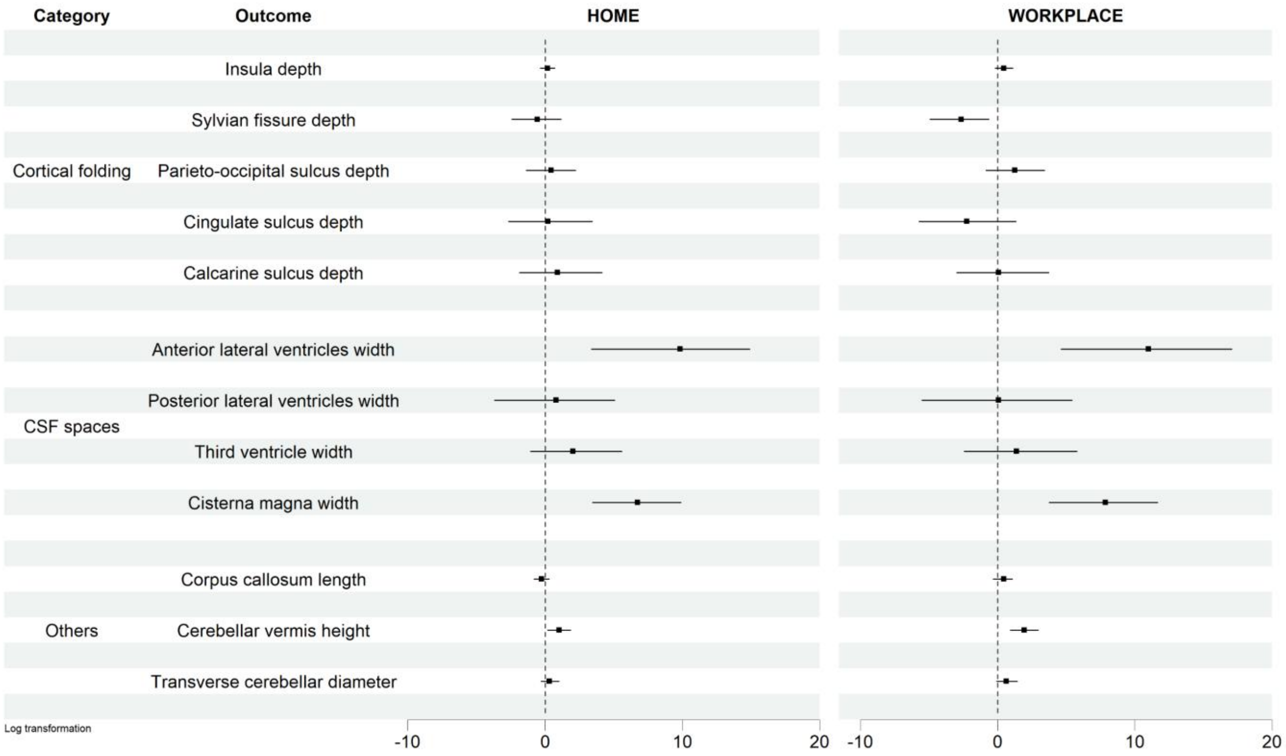

(B) $PM_{2·5}$

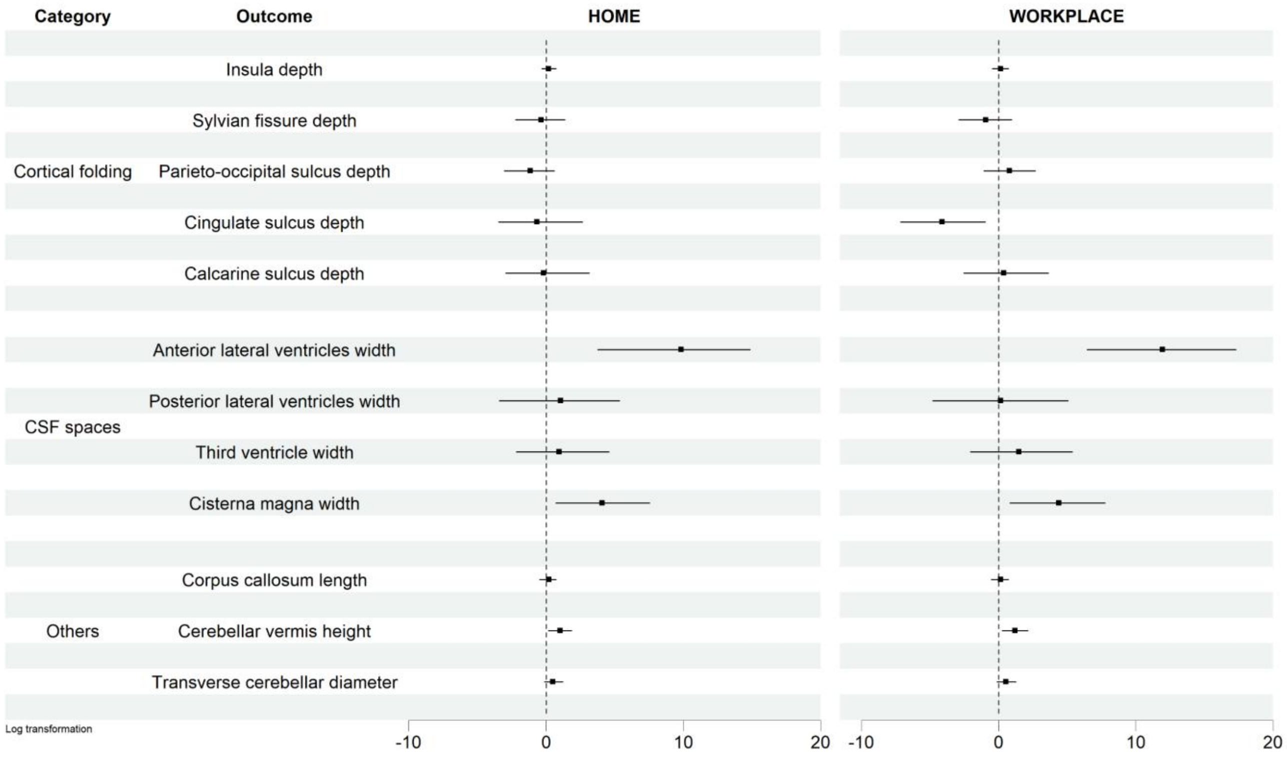
Category
Outcome
HOME
WORKPLACE
Insula depth
Sylvian fissure depth
Cortical folding
Parieto-occipital sulcus depth
Cingulate sulcus depth
Calcarine sulcus depth
Anterior lateral ventricles width
Posterior lateral ventricles width
CSF spaces
Third ventricle width
Cisterna magna width
Corpus callosum length
Others
Cerebellar vermis height
Transverse cerebellar diameter
Log transformation
-10
0
10
20
-10
0
10
20

(C) BC

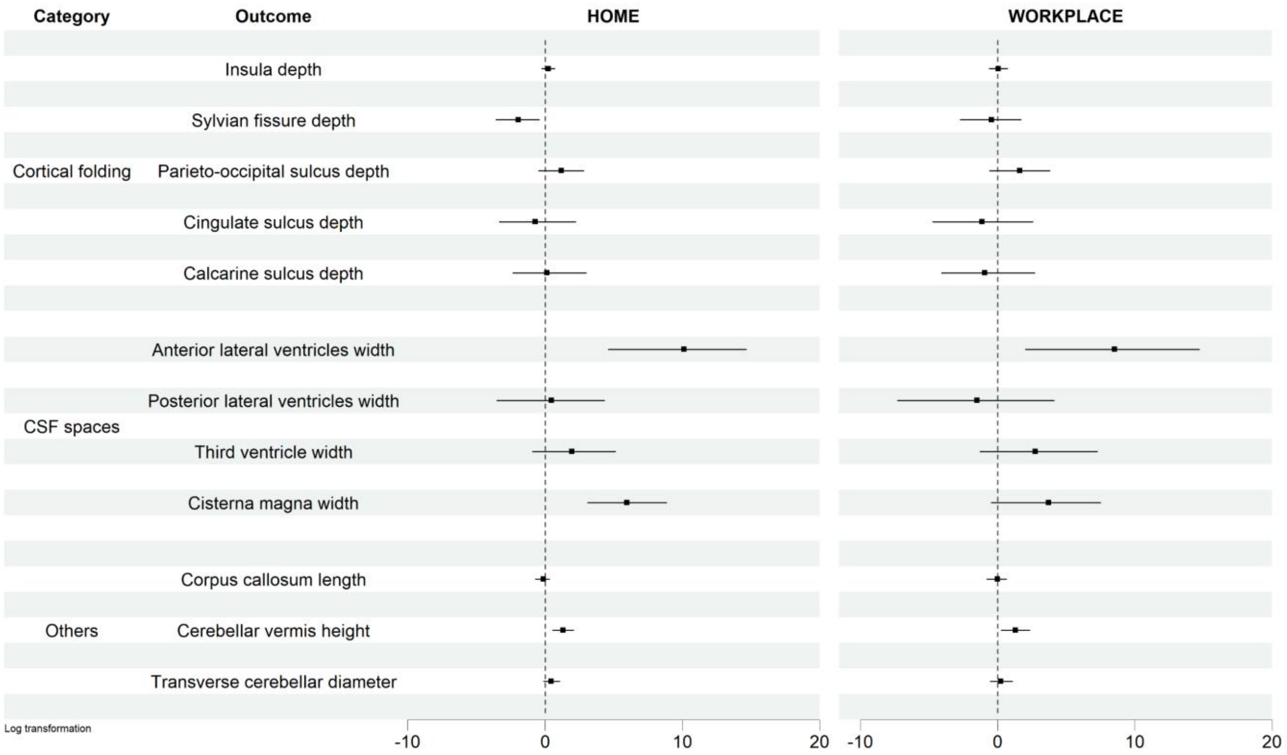

[a] Adjusted for foetal sex (boy vs girl), mother parity (multiparous vs nulliparous), maternal education (with university degree vs without university degree), ethnicity (European vs other), active smoking during pregnancy (no vs yes), passive smoking during pregnancy (no vs yes), alcohol consumption during pregnancy (no vs yes), and gestational age at ultrasound (days), and hospital and rater as random effects.

Abbreviations: N, number of samples; IQR, interquartile range; NO_2, nitrogen dioxide; $PM_{2·5}$, particulate matter with an aerodynamic diameter < 2·5 µm; BC, black carbon; CSF, cerebrospinal fluid; LUR, land use regression.

Table S12: Association of per IQR increases exposure to NO_2, $PM_{2·5}$, and BC in each microenvironment with the percent difference in brain morphological structures (%).[a] (A: NO_2; B: $PM_{2·5}$; C: BC)

(A) NO_2

Category	Outcome	N	Home % (95% CI)	N	Workplace % (95% CI)
Cortical folding	Insula depth	671	0·17 (-0·34, 0·71)	436	0·44 (-0·19, 1·11)
	Sylvian fissure depth	671	-0·56 (-2·44, 1·18)	436	-2·67 (-4·93, -0·64)
	Parieto-occipital sulcus depth	548	0·44 (-1·37, 2·22)	374	1·25 (-0·84, 3·41)
	Cingulate sulcus depth	569	0·20 (-2·67, 3·42)	360	-2·26 (-5·72, 1·33)
	Calcarine sulcus depth	456	0·89 (-1·86, 4·16)	301	0·07 (-2·99, 3·72)
CSF spaces	Anterior lateral ventricles width	469	9·83 (3·37, 14·91)	314	11·00 (4·62, 17·06)
	Posterior lateral ventricles width	655	0·79 (-3·68, 5·08)	420	0·06 (-5·52, 5·42)
	Third ventricle width	679	2·02 (-1·07, 5·60)	444	1·37 (-2·44, 5·78)
	Cisterna magna width	611	6·74 (3·45, 9·90)	402	7·86 (3·76, 11·67)
Others	Corpus callosum length	670	-0·25 (-0·80, 0·29)	433	0·44 (-0·32, 1·09)
	Cerebellar vermis height	643	1·03 (0·18, 1·87)	412	1·95 (0·93, 2·96)
	Transcerebellar diameter	625	0·30 (-0·30, 1·01)	410	0·64 (-0·08, 1·44)

(B) $PM_{2·5}$

Category	Outcome	N	Home<br>% (95% CI)	N	Workplace<br>% (95% CI)
**Cortical folding**	Insula depth	671	0·19 (-0·33, 0·74)	436	0·13 (-0·45, 0·72)
	Sylvian fissure depth	671	-0·36 (-2·23, 1·38)	436	-0·94 (-2·91, 0·94)
	Parieto-occipital sulcus depth	548	-1·16 (-3·04, 0·59)	374	0·79 (-1·09, 2·67)
	Cingulate sulcus depth	569	-0·67 (-3·47, 2·67)	360	-4·12 (-7·15, -0·98)
	Calcarine sulcus depth	456	-0·17 (-2·95, 3·15)	301	0·38 (-2·54, 3·63)
**CSF spaces**	Anterior lateral ventricles width	469	9·85 (3·76, 14·87)	314	11·95 (6·46, 17·30)
	Posterior lateral ventricles width	655	1·05 (-3·42, 5·36)	420	0·15 (-4·81, 5·06)
	Third ventricle width	679	0·95 (-2·17, 4·59)	444	1·48 (-2·06, 5·37)
	Cisterna magna width	611	4·09 (0·70, 7·53)	402	4·39 (0·82, 7·77)
**Others**	Corpus callosum length	670	0·21 (-0·48, 0·72)	433	0·15 (-0·53, 0·73)
	Cerebellar vermis height	643	1·02 (0·16, 1·87)	412	1·20 (0·25, 2·13)
	Transcerebellar diameter	625	0·49 (-0·12, 1·23)	410	0·53 (-0·13, 1·26)

(C) BC

Category	Outcome	N	Home<br>% (95% CI)	N	Workplace<br>% (95% CI)
**Cortical folding**	Insula depth	671	0·22 (-0·25, 0·71)	436	0·05 (-0·61, 0·73)
	Sylvian fissure depth	671	-1·95 (-3·58, -0·42)	436	-0·45 (-2·72, 1·71)
	Parieto-occipital sulcus depth	548	1·18 (-0·48, 2·81)	374	1·60 (-0·59, 3·80)
	Cingulate sulcus depth	569	-0·71 (-3·33, 2·23)	360	-1·15 (-4·73, 2·57)
	Calcarine sulcus depth	456	0·12 (-2·36, 2·99)	301	-0·94 (-4·09, 2·70)
**CSF spaces**	Anterior lateral ventricles width	469	10·13 (4·62, 14·66)	314	8·53 (2·03, 14·71)
	Posterior lateral ventricles width	655	0·45 (-3·50, 4·32)	420	-1·51 (-7·29, 4·12)
	Third ventricle width	679	1·94 (-0·92, 5·13)	444	2·73 (-1·29, 7·28)
	Cisterna magna width	611	5·95 (3·09, 8·85)	402	3·71 (-0·45, 7·51)
**Others**	Corpus callosum length	670	-0·14 (-0·70, 0·33)	433	-0·02 (-0·79, 0·64)
	Cerebellar vermis height	643	1·32 (0·55, 2·08)	412	1·31 (0·27, 2·35)
	Transcerebellar diameter	625	0·43 (-0·12, 1·06)	410	0·23 (-0·54, 1·09)

[a] Adjusted for foetal sex (boy vs girl), mother parity (multiparous vs nulliparous), maternal education (with university degree vs without university degree), ethnicity (European vs other), active smoking during pregnancy (no vs yes), passive smoking during pregnancy (no vs yes), alcohol consumption during pregnancy (no vs yes), and gestational age at ultrasound (days), and hospital and rater as random effects.

Abbreviation: N, number of samples; NO_2, nitrogen dioxide; $PM_{2·5}$, particulate matter with an aerodynamic diameter < 2·5 μm; BC, black carbon; CSF, cerebrospinal fluid; IQR, interquartile range; 95% CI, 95% confidence interval.

Figure S21: Association of per IQR increases exposure to NO_2, $PM_{2\cdot5}$, and BC with the percent difference in brain morphological structures (%).[a] (A: Land use regression model, B: Dispersion models)

(A) Land use regression model

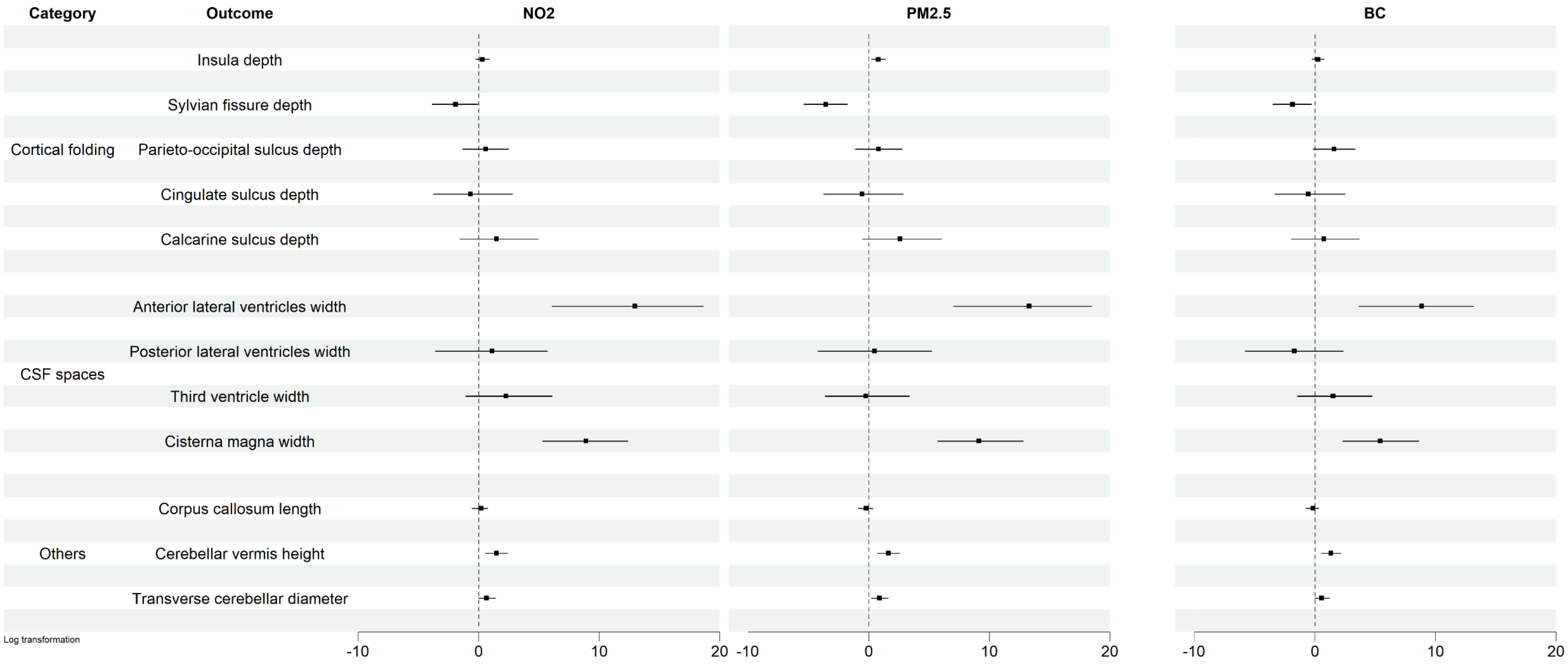

(B) Dispersion models

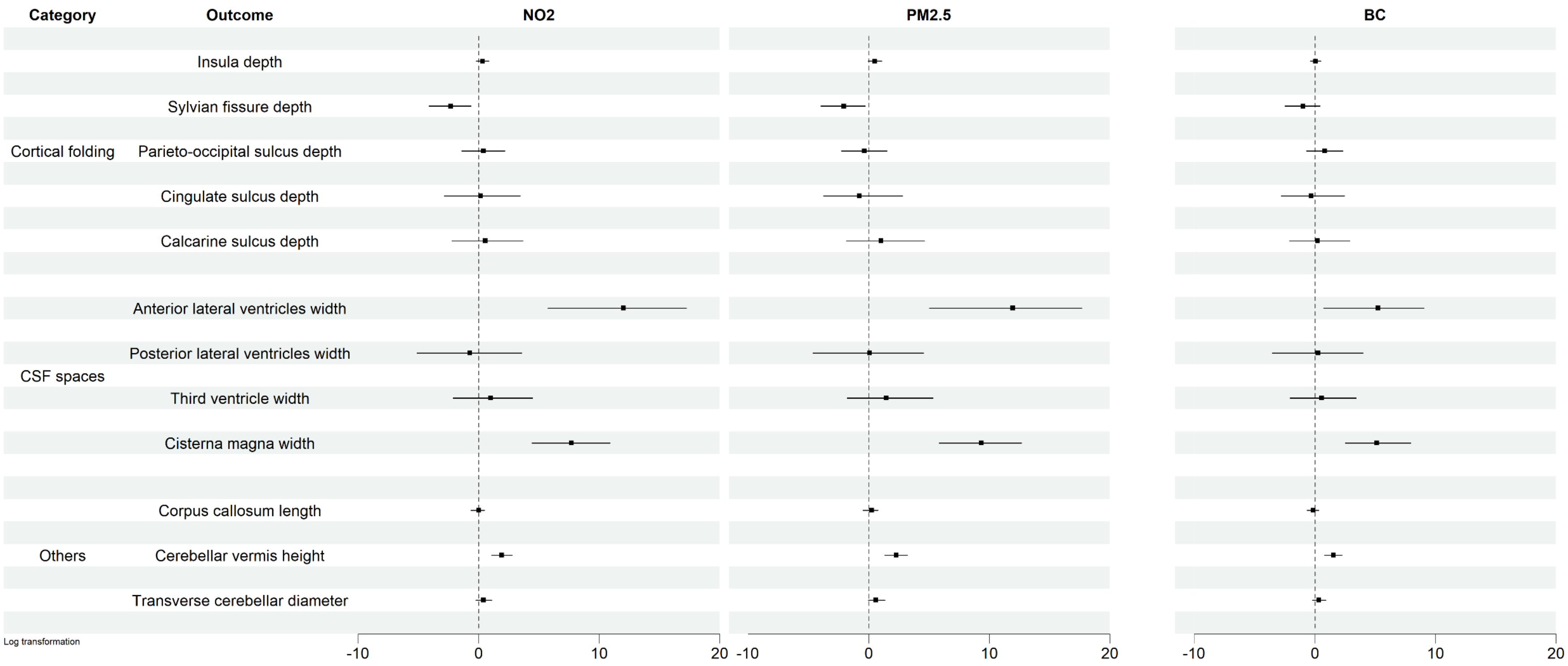

[a] Adjusted for foetal sex (boy vs girl), mother parity (multiparous vs nulliparous), maternal education (with university degree vs without university degree), ethnicity (European vs other), active smoking during pregnancy (no vs yes), passive smoking during pregnancy (no vs yes), alcohol consumption during pregnancy (no vs yes), and gestational age at ultrasound (days), and hospital and rater as random effects.

Abbreviations: N, number of samples; IQR, interquartile range; NO_2, nitrogen dioxide; $PM_{2·5}$, particulate matter with an aerodynamic diameter < 2·5 µm; BC, black carbon; CSF, cerebrospinal fluid.

Table S13: Association of per IQR increases exposure to NO_2, $PM_{2·5}$, and BC with the percent difference in brain morphological structures (%).[a] (A: Land use regression model, B: Dispersion models)

(A) Land use regression model

Category	Outcome	N	NO_2 % (95% CI)	$PM_{2·5}$ % (95% CI)	BC % (95% CI)
Cortical folding	Insula depth	675	0·30 (-0·25, 0·87)	0·80 (0·23, 1·38)	0·25 (-0·24, 0·75)
	Sylvian fissure depth	675	-1·91 (-3·85, -0·09)	-3·55 (-5·38, -1·76)	-1·84 (-3·49, -0·26)
	Parieto-occipital sulcus depth	551	0·58 (-1·35, 2·49)	0·83 (-1·12, 2·79)	1·59 (-0·14, 3·32)
	Cingulate sulcus depth	572	-0·68 (-3·77, 2·79)	-0·54 (-3·75, 2·86)	-0·54 (-3·30, 2·48)
	Calcarine sulcus depth	459	1·46 (-1·55, 4·92)	2·60 (-0·50, 6·02)	0·74 (-1·92, 3·67)
CSF spaces	Anterior lateral ventricles width	473	12·96 (6·08, 18·59)	13·32 (7·06, 18·48)	8·86 (3·65, 13·14)
	Posterior lateral ventricles width	659	1·10 (-3·61, 5·71)	0·48 (-4·21, 5·22)	-1·69 (-5·76, 2·34)
	Third ventricle width	683	2·27 (-1·08, 6·08)	-0·24 (-3·61, 3·39)	1·51 (-1·46, 4·75)
	Cisterna magna width	614	8·89 (5·30, 12·37)	9·16 (5·72, 12·82)	5·41 (2·33, 8·63)
Others	Corpus callosum length	674	0·19 (-0·57, 0·73)	-0·21 (-0·87, 0·35)	-0·16 (-0·73, 0·33)
	Cerebellar vermis height	647	1·48 (0·56, 2·38)	1·63 (0·72, 2·55)	1·34 (0·54, 2·14)
	Transcerebellar diameter	628	0·65 (-0·01, 1·39)	0·90 (0·23, 1·61)	0·56 (-0·02, 1·19)

(B) Dispersion models

Category	Outcome	N	NO_2 % (95% CI)	$PM_{2·5}$ % (95% CI)	BC % (95% CI)
**Cortical folding**	Insula depth	673	0·31 (-0·21, 0·85)	0·51 (-0·03, 1·09)	0·06 (-0·38, 0·51)
	Sylvian fissure depth	673	-2·33 (-4·11, -0·65)	-2·07 (-3·98, -0·28)	-0·98 (-2·47, 0·43)
	Parieto-occipital sulcus depth	549	0·39 (-1·43, 2·19)	-0·35 (-2·28, 1·54)	0·82 (-0·72, 2·33)
	Cingulate sulcus depth	570	0·15 (-2·85, 3·43)	-0·77 (-3·74, 2·81)	-0·30 (-2·79, 2·46)
	Calcarine sulcus depth	458	0·54 (-2·21, 3·64)	1·03 (-1·84, 4·60)	0·24 (-2·09, 2·88)
**CSF spaces**	Anterior lateral ventricles width	471	12·00 (5·74, 17·22)	11·96 (5·04, 17·66)	5·23 (0·72, 9·04)
	Posterior lateral ventricles width	657	-0·73 (-5·12, 3·58)	0·08 (-4·62, 4·56)	0·25 (-3·53, 3·99)
	Third ventricle width	681	0·99 (-2·15, 4·47)	1·47 (-1·78, 5·35)	0·56 (-2·08, 3·44)
	Cisterna magna width	613	7·69 (4·42, 10·90)	9·35 (5·85, 12·69)	5·13 (2·53, 7·94)
**Others**	Corpus callosum length	672	-0·00 (-0·65, 0·48)	0·25 (-0·49, 0·79)	-0·13 (-0·65, 0·31)
	Cerebellar vermis height	645	1·92 (1·07, 2·77)	2·28 (1·36, 3·20)	1·53 (0·80, 2·24)
	Transcerebellar diameter	627	0·39 (-0·23, 1·07)	0·60 (-0·02, 1·36)	0·32 (-0·20, 0·91)

[a] Adjusted for foetal sex (boy vs girl), mother parity (multiparous vs nulliparous), maternal education (with university degree vs without university degree), ethnicity (European vs other), active smoking during pregnancy (no vs yes), passive smoking during pregnancy (no vs yes), alcohol consumption during pregnancy (no vs yes), and gestational age at ultrasound (days), and hospital and rater as random effects.

Abbreviation: N, number of samples; NO_2, nitrogen dioxide; $PM_{2·5}$, particulate matter with an aerodynamic diameter < 2·5 μm; BC, black carbon; CSF, cerebrospinal fluid; IQR, interquartile range; 95% CI, 95% confidence interval.

Figure S22: Association of per IQR increases exposure to NO_2, $PM_{2\cdot5}$, and BC with the percent difference in anterior and posterior horn lateral ventricles for both left and right (%). [a] (A: Anterior horn lateral ventricles; B: Posterior horn lateral ventricles)

(A) (B)

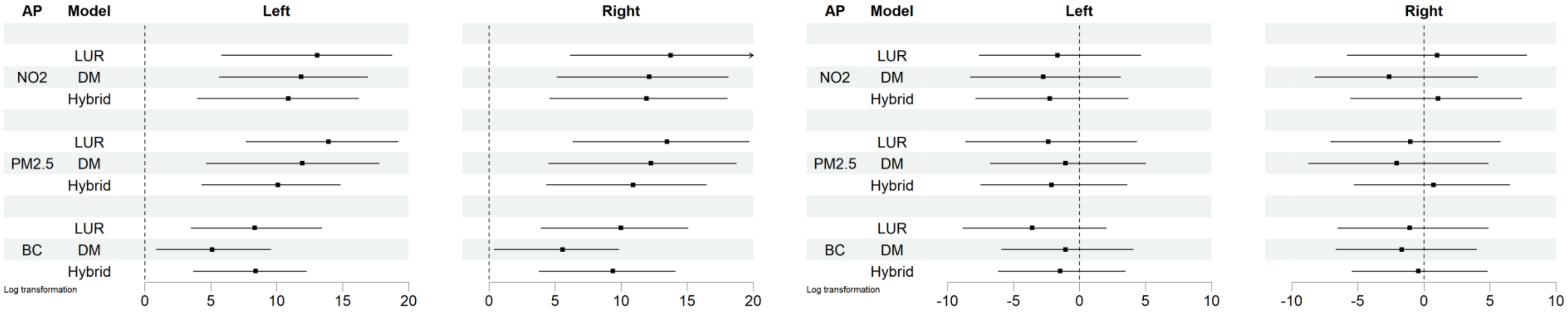

[a] Adjusted for foetal sex (boy vs girl), mother parity (multiparous vs nulliparous), maternal education (with university degree vs without university degree), ethnicity (European vs other), active smoking during pregnancy (no vs yes), passive smoking during pregnancy (no vs yes), alcohol consumption during pregnancy (no vs yes), and gestational age at ultrasound (days), and hospital and rater as random effects.

Abbreviation: N, number of samples; NO_2, nitrogen dioxide; $PM_{2\cdot5}$, particulate matter with an aerodynamic diameter < 2·5 µm; BC, black carbon; CSF, cerebrospinal fluid; IQR, interquartile range; 95% CI, 95% confidence interval; LUR, land use regression.

Table S14: Association of per IQR increases exposure to NO_2, $PM_{2·5}$, and BC with the percent difference in anterior and posterior horn lateral ventricles for both left and right (%). [a]

**Air pollutants**	**Model**	**Anterior horn lateral ventricles**				**Posterior horn lateral ventricles**			
		N	Left % (95% CI)	N	Right % (95% CI)	N	Left % (95% CI)	N	Right % (95% CI)
**NO_2**	LUR models	496	5·11 (0·87, 9·53)	491	5·57 (0·38, 9·82)	325	-1·05 (-5·91, 4·07)	339	-1·68 (-6·67, 3·99)
	Dispersion models	496	8·41 (3·68, 12·24)	491	9·38 (3·77, 14·11)	325	-1·47 (-6·17, 3·47)	339	-0·43 (-5·45, 4·79)
	Hybrid models	498	8·34 (3·50, 13·41)	493	9·98 (3·93, 15·08)	326	-3·57 (-8·84, 2·01)	340	-1·08 (-6·53, 4·86)
**$PM_{2·5}$**	LUR models	496	11·85 (5·64, 16·88)	491	12·13 (5·12, 18·12)	325	-2·74 (-8·27, 3·11)	339	-2·62 (-8·25, 4·07)
	Dispersion models	496	10·89 (3·97, 16·19)	491	11·92 (4·56, 18·05)	325	-2·26 (-7·87, 3·69)	339	1·07 (-5·58, 7·41)
	Hybrid models	498	13·07 (5·83, 18·72)	493	13·76 (6·14, 20·25)	326	-1·67 (-7·60, 4·64)	340	1·01 (-5·81, 7·77)
**BC**	LUR models	496	11·95 (4·65, 17·75)	491	12·26 (4·50, 18·73)	325	-1·05 (-6·77, 5·02)	339	-2·07 (-8·76, 4·87)
	Dispersion models	496	10·09 (4·30, 14·81)	491	10·91 (4·34, 16·45)	325	-2·11 (-7·50, 3·60)	339	0·72 (-5·31, 6·49)
	Hybrid models	498	13·93 (7·68, 19·20)	493	13·47 (6·34, 19·69)	326	-2·37 (-8·64, 4·32)	340	-1·02 (-7·08, 5·79)

[a] Adjusted for foetal sex (boy vs girl), mother parity (multiparous vs nulliparous), maternal education (with university degree vs without university degree), ethnicity (European vs other), active smoking during pregnancy (no vs yes), passive smoking during pregnancy (no vs yes), alcohol consumption during pregnancy (no vs yes), and gestational age at ultrasound (days), and hospital and rater as random effects.

Abbreviation: LUR models, land use regression models; N, number of samples; NO_2, nitrogen dioxide; $PM_{2·5}$, particulate matter with an aerodynamic diameter < 2·5 μm; BC, black carbon; CSF, cerebrospinal fluid; IQR, interquartile range; 95% CI, 95% confidence interval.

Figure S23: Association of per IQR increases exposure to NO_2, $PM_{2\cdot5}$, and BC with the percent difference in brain morphological structures after removing the participants with active and passive smoking exposure during pregnancy (%).[a] (A: Removing Active Smoking Exposure Participants; B: Removing Passive Smoking Exposure Participants)

(A) Active Smoking

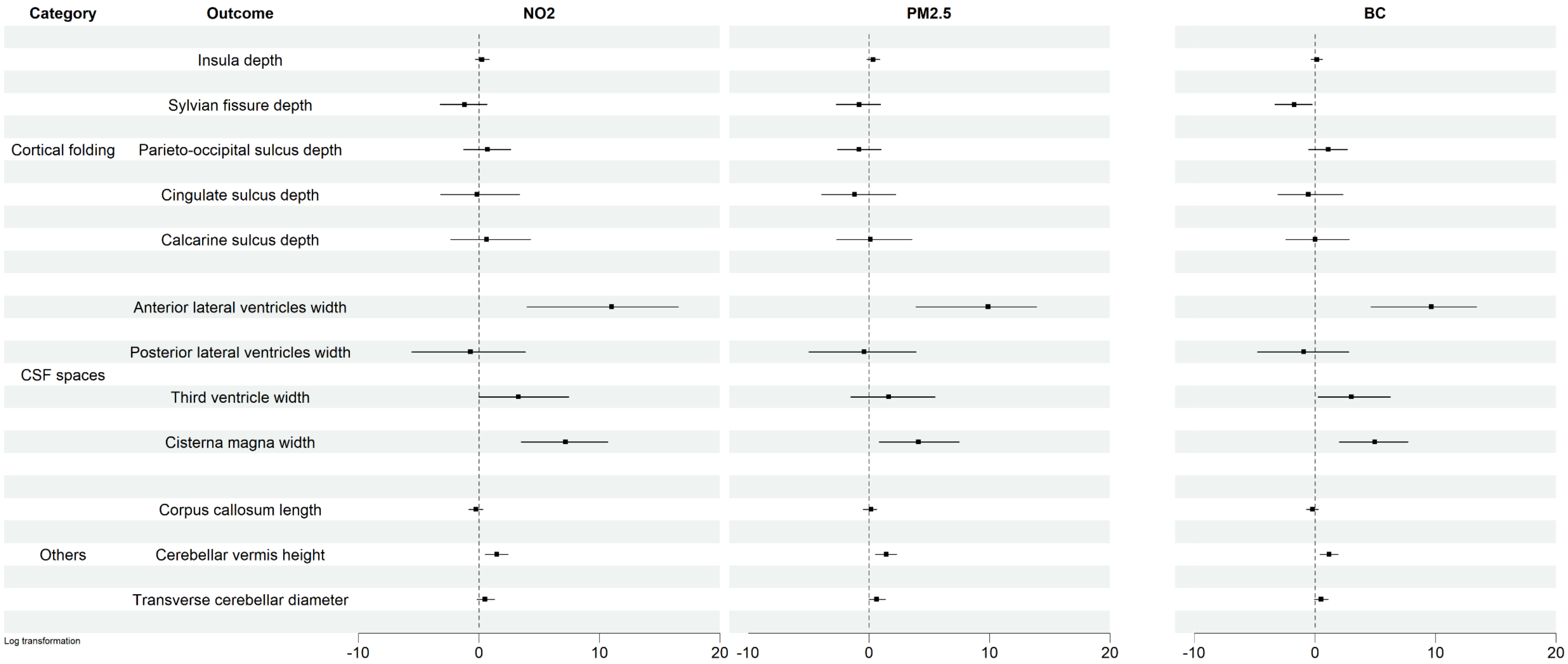

(B) Passive Smoking

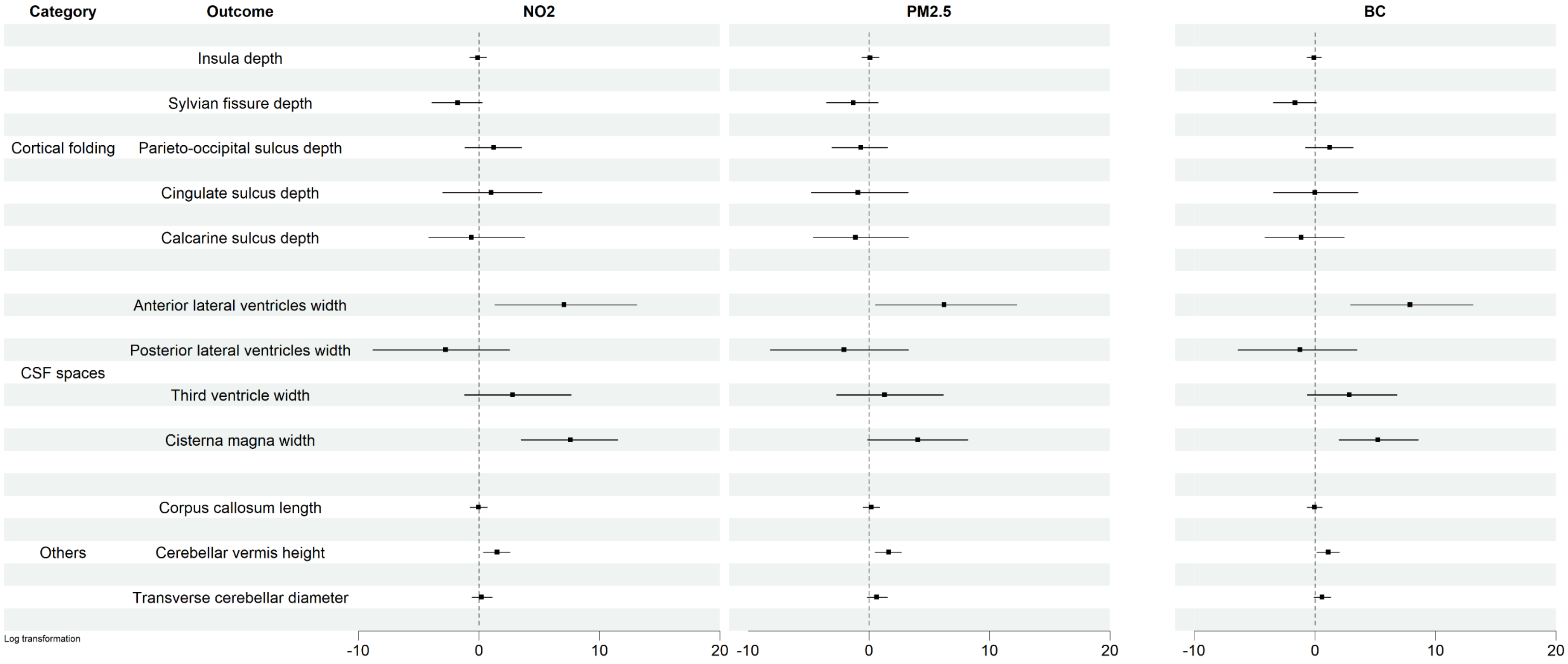

[a] Adjusted for foetal sex (boy vs girl), mother parity (multiparous vs nulliparous), maternal education (with university degree vs without university degree), ethnicity (European vs other), passive smoking during pregnancy (no vs yes) or active smoking during pregnancy (no vs yes), alcohol consumption during pregnancy (no vs yes), and gestational age at ultrasound (days), and hospital and rater as random effects.

Abbreviation: N, number of samples; NO_2, nitrogen dioxide; $PM_{2·5}$, particulate matter with an aerodynamic diameter < 2·5 µm; BC, black carbon; CSF, cerebrospinal fluid; IQR, interquartile range; 95% CI, 95% confidence interval; LUR, land use regression.

Table S15: Association of per IQR increases exposure to NO_2, $PM_{2·5}$, and BC with the percent difference in brain morphological structures after removing the participants with active and passive smoking exposure during pregnancy (%).[a] (A: Removing Active Smoking Exposure Participants; B: Removing Passive Smoking Exposure Participants)

(A) Active Smoking

Category	Outcome	N	NO_2 % (95% CI)	$PM_{2.5}$ % (95% CI)	BC % (95% CI)
Cortical folding	Insula depth	612	0·25 (-0·31, 0·84)	0·35 (-0·18, 0·91)	0·14 (-0·32, 0·62)
	Sylvian fissure depth	612	-1·20 (-3·25, 0·66)	-0·80 (-2·71, 0·96)	-1·73 (-3·32, -0·23)
	Parieto-occipital sulcus depth	501	0·70 (-1·29, 2·64)	-0·83 (-2·61, 1·02)	1·11 (-0·52, 2·71)
	Cingulate sulcus depth	521	-0·18 (-3·20, 3·36)	-1·19 (-3·92, 2·22)	-0·54 (-3·07, 2·32)
	Calcarine sulcus depth	416	0·63 (-2·34, 4·25)	0·13 (-2·65, 3·55)	0·02 (-2·41, 2·84)
CSF spaces	Anterior lateral ventricles width	439	11·02 (3·99, 16·51)	9·89 (3·93, 13·92)	9·66 (4·65, 13·41)
	Posterior lateral ventricles width	599	-0·72 (-5·58, 3·86)	-0·38 (-4·98, 3·92)	-0·94 (-4·77, 2·80)
	Third ventricle width	619	3·28 (-0·04, 7·44)	1·63 (-1·51, 5·48)	3·02 (0·23, 6·25)
	Cisterna magna width	559	7·19 (3·50, 10·69)	4·12 (0·85, 7·48)	4·95 (2·02, 7·73)
Others	Corpus callosum length	614	-0·25 (-0·85, 0·34)	0·19 (-0·48, 0·66)	-0·21 (-0·71, 0·29)
	Cerebellar vermis height	586	1·46 (0·51, 2·38)	1·43 (0·54, 2·29)	1·18 (0·42, 1·92)
	Transcerebellar diameter	572	0·50 (-0·17, 1·27)	0·63 (0·01, 1·38)	0·50 (-0·05, 1·10)

(B) Passive Smoking

Category	Outcome	N	NO_2<br>% (95% CI)	$PM_{2.5}$<br>% (95% CI)	BC<br>% (95% CI)
Cortical folding	Insula depth	387	-0·11 (-0·77, 0·60)	0·09 (-0·59, 0·84)	-0·08 (-0·65, 0·52)
	Sylvian fissure depth	387	-1·77 (-3·93, 0·27)	-1·30 (-3·50, 0·78)	-1·64 (-3·47, 0·12)
	Parieto-occipital sulcus depth	304	1·21 (-1·19, 3·52)	-0·67 (-3·08, 1·56)	1·22 (-0·79, 3·17)
	Cingulate sulcus depth	326	1·02 (-3·01, 5·22)	-0·90 (-4·78, 3·26)	0·00 (-3·43, 3·56)
	Calcarine sulcus depth	254	-0·64 (-4·18, 3·74)	-1·12 (-4·58, 3·28)	-1·13 (-4·12, 2·41)
CSF spaces	Anterior lateral ventricles width	272	7·04 (1·32, 13·08)	6·24 (0·54, 12·26)	7·90 (2·96, 13·09)
	Posterior lateral ventricles width	371	-2·78 (-8·81, 2·54)	-2·07 (-8·19, 3·27)	-1·24 (-6·39, 3·48)
	Third ventricle width	388	2·79 (-1·21, 7·65)	1·32 (-2·69, 6·17)	2·84 (-0·63, 6·81)
	Cisterna magna width	352	7·60 (3·49, 11·51)	4·06 (-0·12, 8·21)	5·21 (1·99, 8·56)
Others	Corpus callosum length	384	-0·04 (-0·75, 0·68)	0·21 (-0·49, 0·92)	-0·03 (-0·65, 0·59)
	Cerebellar vermis height	373	1·49 (0·38, 2·56)	1·63 (0·52, 2·68)	1·11 (0·17, 2·02)
	Transcerebellar diameter	359	0·20 (-0·56, 1·07)	0·63 (-0·13, 1·54)	0·58 (-0·07, 1·29)

[a] Adjusted for foetal sex (boy vs girl), mother parity (multiparous vs nulliparous), maternal education (with university degree vs without university degree), ethnicity (European vs other), active smoking during pregnancy (no vs yes) or passive smoking during pregnancy (no vs yes), alcohol consumption during pregnancy (no vs yes), and gestational age at ultrasound (days), and hospital and rater as random effects.

Abbreviation: LUR models, land use regression models; N, number of samples; NO_2, nitrogen dioxide; $PM_{2·5}$, particulate matter with an aerodynamic diameter < 2·5 µm; BC, black carbon; CSF, cerebrospinal fluid; IQR, interquartile range; 95% CI, 95% confidence interval.

Figure S24: Association of per IQR increases exposure to NO_2, $PM_{2\cdot5}$, and BC with the percent difference in brain morphological structures adjusting the p-value for multiple comparisons (%).[a] (A: NO_2; B: $PM_{2\cdot5}$; C: BC)

(A) NO_2

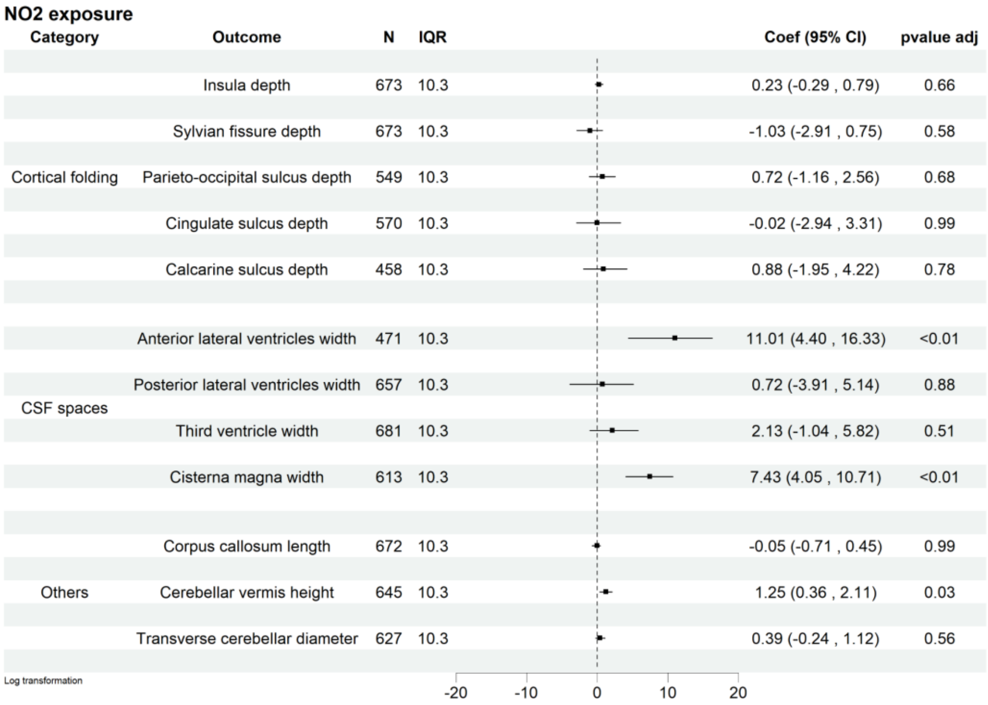

(B) $PM_{2\cdot5}$

PM2.5 exposure

Category	Outcome	N	IQR	Coef (95% CI)	pvalue adj
Cortical folding	Insula depth	673	1.4	0.25 (-0.25 , 0.78)	0.63
	Sylvian fissure depth	673	1.4	-0.43 (-2.19 , 1.24)	0.83
	Parieto-occipital sulcus depth	549	1.4	-0.77 (-2.55 , 0.93)	0.66
	Cingulate sulcus depth	570	1.4	-1.09 (-3.71 , 2.12)	0.68
	Calcarine sulcus depth	458	1.4	0.08 (-2.59 , 3.21)	0.99
CSF spaces	Anterior lateral ventricles width	471	1.4	10.49 (4.61 , 15.37)	<0.01
	Posterior lateral ventricles width	657	1.4	0.92 (-3.38 , 5.07)	0.83
	Third ventricle width	681	1.4	1.28 (-1.72 , 4.77)	0.67
	Cisterna magna width	613	1.4	4.27 (1.02 , 7.55)	0.03
Others	Corpus callosum length	672	1.4	0.29 (-0.38 , 0.68)	0.63
	Cerebellar vermis height	645	1.4	1.16 (0.33 , 1.97)	0.03
	Transverse cerebellar diameter	627	1.4	0.58 (0.00 , 1.29)	0.22

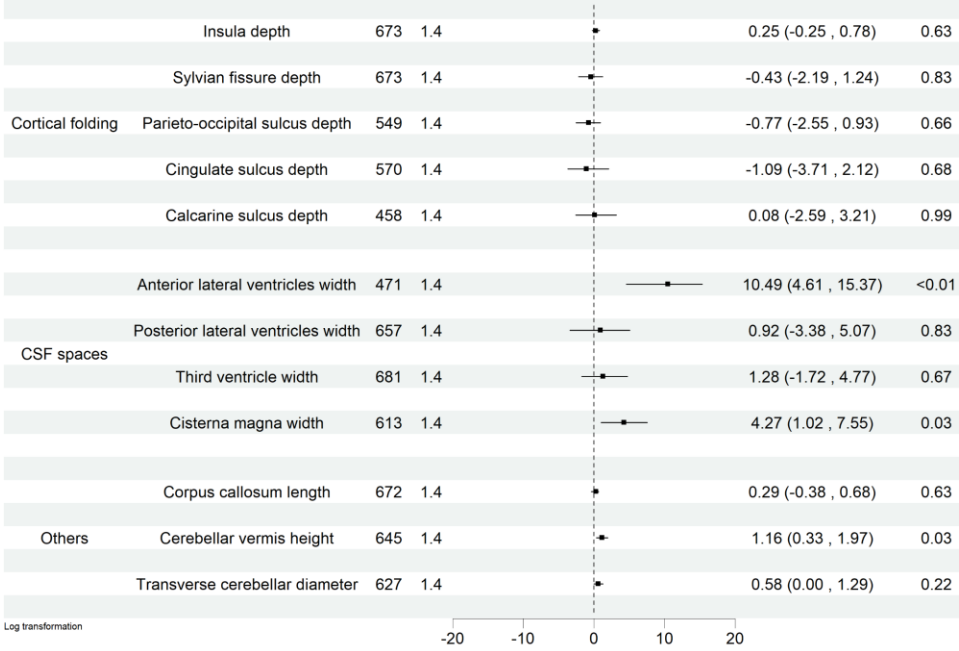

Log transformation

(C) BC

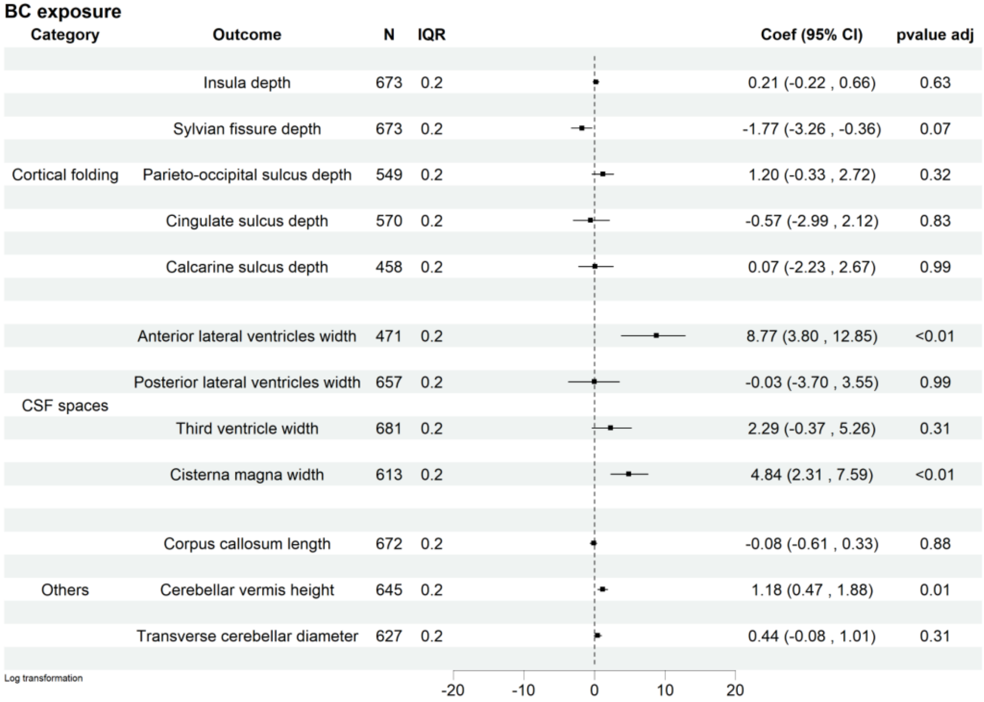

[a] Adjusted for foetal sex (girl *vs.* boy), parity (multiparous *vs.* nulliparous), maternal education (with university degree *vs.* without university degree), ethnicity (European *vs.* other), active smoking during pregnancy (no *vs.* yes), passive smoking during pregnancy (no *vs.* yes), alcohol consumption during pregnancy (no *vs.* yes), and gestational age at ultrasound (days), and the rater and hospital as random effects.

Abbreviations: N, number of samples; IQR, interquartile range; NO_2, nitrogen dioxide; $PM_{2·5}$, particulate matter with an aerodynamic diameter < 2·5 µm; BC, black carbon; CSF, cerebrospinal fluid.

Figure S25: Association of per IQR increases exposure to NO_2, $PM_{2\cdot5}$, and BC with the percent difference in brain morphological structures (%) stratified by foetal sex. [a] (A: NO_2; B: $PM_{2\cdot5}$; C: BC)

(A)NO_2

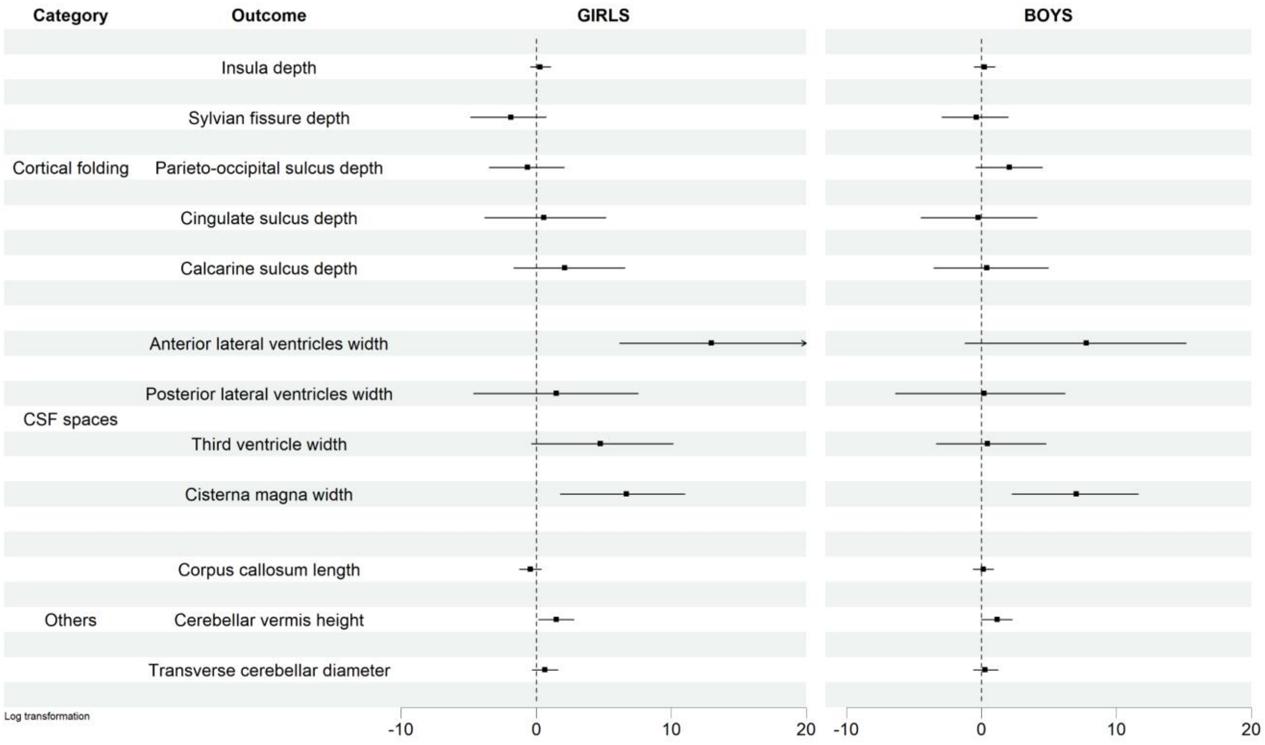

(B) $PM_{2\cdot5}$

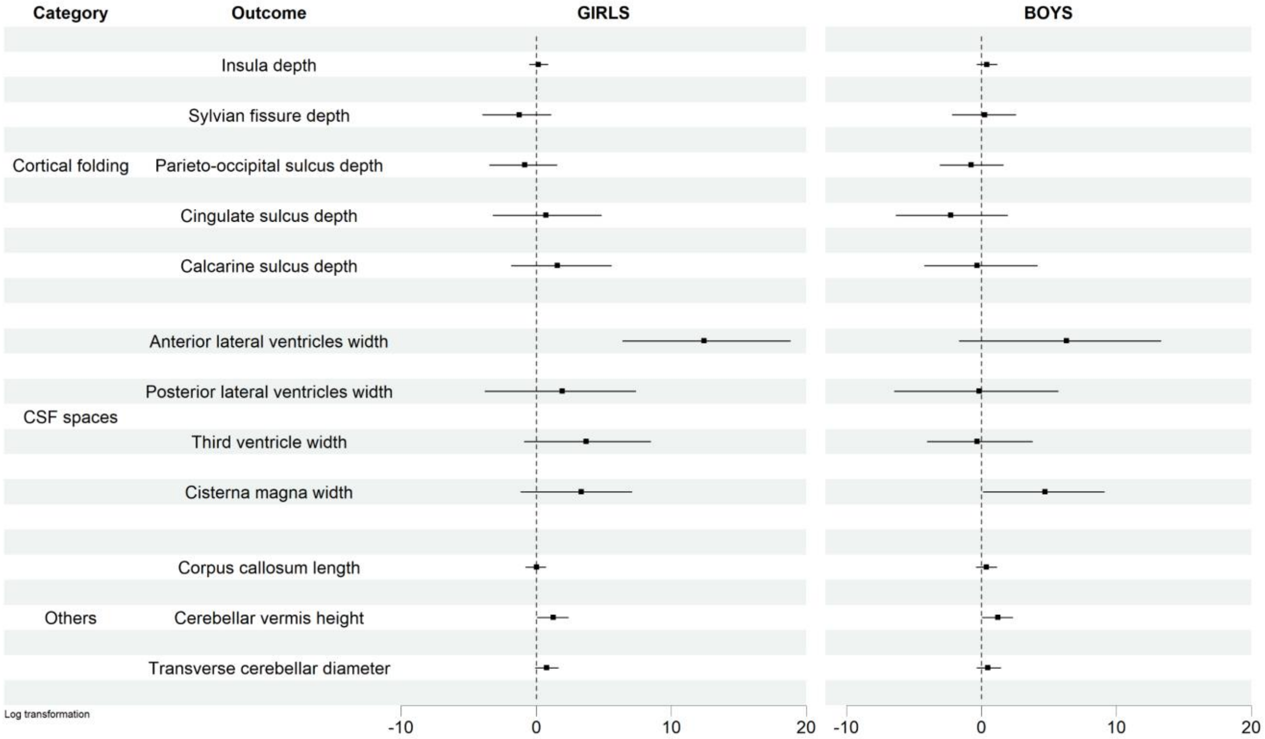
Category
Outcome
GIRLS
BOYS
Insula depth
Sylvian fissure depth
Cortical folding
Parieto-occipital sulcus depth
Cingulate sulcus depth
Calcarine sulcus depth
Anterior lateral ventricles width
Posterior lateral ventricles width
CSF spaces
Third ventricle width
Cisterna magna width
Corpus callosum length
Others
Cerebellar vermis height
Transverse cerebellar diameter
Log transformation
-10
0
10
20
-10
0
10
20

(C) BC

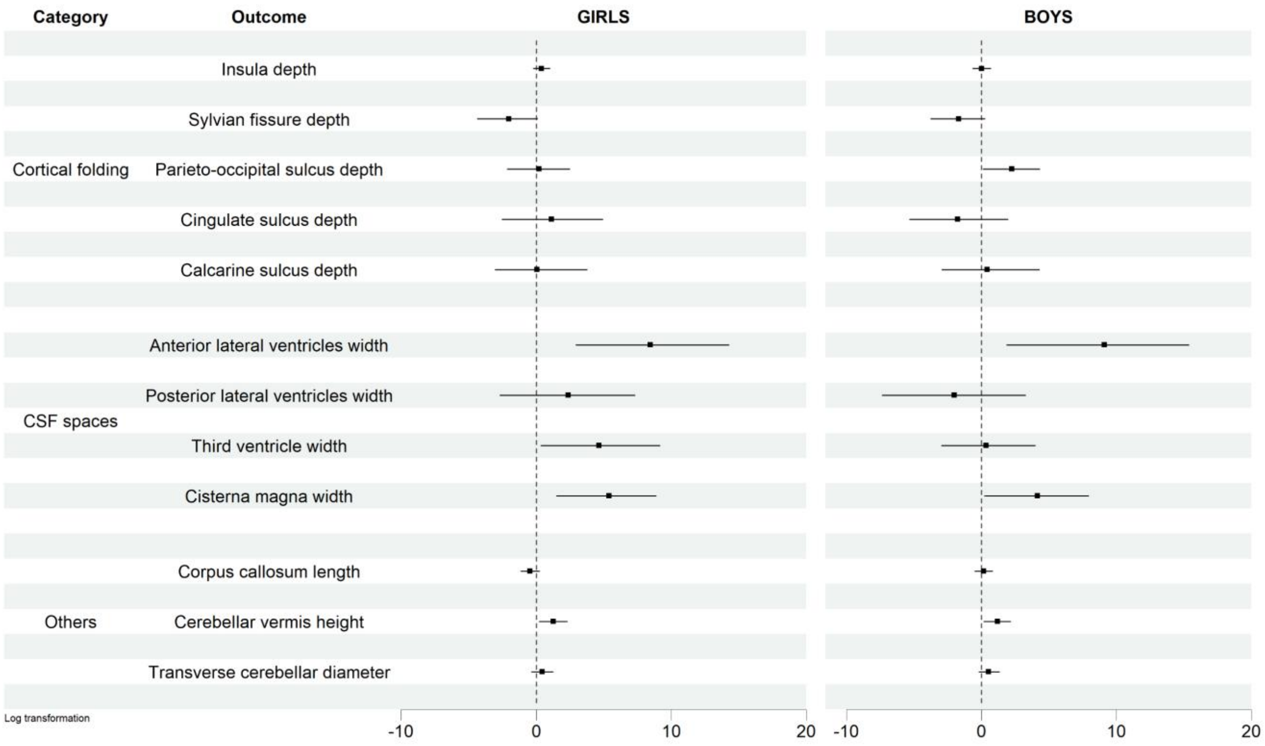

[a] Adjusted for foetal sex (boy vs girl), mother parity (multiparous vs nulliparous), maternal education (with university degree vs without university degree), ethnicity (European vs other), active smoking during pregnancy (no vs yes), passive smoking during pregnancy (no vs yes), alcohol consumption during pregnancy (no vs yes), and gestational age at ultrasound (days), and the rater and hospital as random effects.

Abbreviations: N, number of samples; IQR, interquartile range; NO_2, nitrogen dioxide; $PM_{2·5}$, particulate matter with an aerodynamic diameter < 2·5 µm; BC, black carbon; CSF, cerebrospinal fluid

Table S16: Association of per IQR increases exposure to NO_2, $PM_{2·5}$, and BC with the percent difference in brain morphological structures (%) stratified by foetal sex. [a] (A: NO_2; B: $PM_{2·5}$; C: BC)

(A) NO_2

Category	Outcome	N	Boys % (95% CI)	N	Girls % (95% CI)	Interaction p-value
Cortical folding	Insula depth	352	0·23 (-0·54, 1·01)	321	0·29 (-0·43, 1·07)	0.65
	Sylvian fissure depth	352	-0·37 (-2·94, 1·98)	321	-1·87 (-4·87, 0·74)	0.69
	Parieto-occipital sulcus depth	276	2·07 (-0·42, 4·53)	273	-0·65 (-3·49, 2·06)	0.39
	Cingulate sulcus depth	302	-0·23 (-4·48, 4·12)	268	0·56 (-3·82, 5·15)	0.84
	Calcarine sulcus depth	242	0·40 (-3·52, 4·96)	216	2·10 (-1·66, 6·57)	0.87
CSF spaces	Anterior lateral ventricles width	248	7·78 (-1·21, 15·16)	223	12·97 (6·17, 20·21)	0.06
	Posterior lateral ventricles width	348	0·19 (-6·36, 6·20)	309	1·50 (-4·64, 7·54)	0.91
	Third ventricle width	352	0·44 (-3·34, 4·78)	329	4·75 (-0·36, 10·13)	0.31
	Cisterna magna width	325	7·03 (2·27, 11·61)	288	6·69 (1·79, 11·01)	0.76
Others	Corpus callosum length	347	0·15 (-0·61, 0·91)	325	-0·44 (-1·26, 0·38)	0.24
	Cerebellar vermis height	336	1·16 (-0·00, 2·29)	309	1·50 (0·16, 2·79)	0.63
	Transcerebellar diameter	332	0·26 (-0·58, 1·23)	295	0·64 (-0·30, 1·60)	0.79

(B) $PM_{2·5}$

Category	Outcome	N	Boys<br>% (95% CI)	N	Girls<br>% (95% CI)	Interaction<br>p-value
Cortical folding	Insula depth	352	0·39 (-0·34, 1·16)	321	0·14 (-0·51, 0·87)	0.85
	Sylvian fissure depth	352	0·25 (-2·17, 2·54)	321	-1·27 (-3·97, 1·08)	0.57
	Parieto-occipital sulcus depth	276	-0·76 (-3·06, 1·63)	273	-0·85 (-3·46, 1·54)	0.75
	Cingulate sulcus depth	302	-2·28 (-6·33, 1·94)	268	0·72 (-3·21, 4·81)	0.32
	Calcarine sulcus depth	242	-0·33 (-4·21, 4·15)	216	1·56 (-1·84, 5·56)	0.87
CSF spaces	Anterior lateral ventricles width	248	6·31 (-1·65, 13·30)	223	12·45 (6·42, 18·82)	0.06
	Posterior lateral ventricles width	348	-0·16 (-6·45, 5·68)	309	1·93 (-3·78, 7·37)	0.86
	Third ventricle width	352	-0·32 (-4·01, 3·79)	329	3·69 (-0·88, 8·47)	0.38
	Cisterna magna width	325	4·72 (0·12, 9·11)	288	3·35 (-1·15, 7·07)	0.86
Others	Corpus callosum length	347	0·37 (-0·38, 1·13)	325	0·02 (-0·78, 0·71)	0.34
	Cerebellar vermis height	336	1·21 (0·06, 2·31)	309	1·25 (0·06, 2·38)	0.87
	Transcerebellar diameter	332	0·48 (-0·34, 1·44)	295	0·77 (-0·07, 1·62)	0.80

(C) BC

Category	Outcome	N	Boys<br>% (95% CI)	N	Girls<br>% (95% CI)	Interaction<br>p-value
**Cortical folding**	Insula depth	352	0·02 (-0·64, 0·69)	321	0·38 (-0·22, 1·02)	0.29
	Sylvian fissure depth	352	-1·68 (-3·76, 0·26)	321	-2·02 (-4·36, 0·10)	0.99
	Parieto-occipital sulcus depth	276	2·24 (0·13, 4·33)	273	0·20 (-2·14, 2·48)	0.39
	Cingulate sulcus depth	302	-1·75 (-5·31, 1·95)	268	1·12 (-2·54, 4·93)	0.37
	Calcarine sulcus depth	242	0·43 (-2·94, 4·29)	216	0·04 (-3·06, 3·76)	0.80
**CSF spaces**	Anterior lateral ventricles width	248	9·10 (1·89, 15·37)	223	8·45 (2·93, 14·27)	0.53
	Posterior lateral ventricles width	348	-2·01 (-7·35, 3·26)	309	2·36 (-2·68, 7·32)	0.28
	Third ventricle width	352	0·35 (-2·96, 3·98)	329	4·65 (0·34, 9·15)	0.17
	Cisterna magna width	325	4·14 (0·22, 7·94)	288	5·40 (1·51, 8·87)	0.45
**Others**	Corpus callosum length	347	0·16 (-0·49, 0·82)	325	-0·46 (-1·15, 0·24)	0.20
	Cerebellar vermis height	336	1·19 (0·19, 2·16)	309	1·27 (0·21, 2·31)	0.73
	Transcerebellar diameter	332	0·52 (-0·19, 1·33)	295	0·43 (-0·34, 1·24)	0.74

[a] Adjusted for foetal sex (boy vs girl), mother parity (multiparous vs nulliparous), maternal education (with university degree vs without university degree), ethnicity (European vs other), active smoking during pregnancy (no vs yes), passive smoking during pregnancy (no vs yes), alcohol consumption during pregnancy (no vs yes), and gestational age at ultrasound (days), and the rater and hospital as random effects.

Abbreviation: N, number of samples; NO_2, nitrogen dioxide; $PM_{2·5}$, particulate matter with an aerodynamic diameter < 2·5 μm; BC, black carbon; CSF, cerebrospinal fluid; IQR, interquartile range; 95% CI, 95% confidence interval.
